# Secreted breast tumor interstitial fluid microRNAs and their target genes are associated with triple-negative breast cancer, tumor grade, and immune infiltration

**DOI:** 10.1186/s13058-020-01295-6

**Published:** 2020-06-30

**Authors:** Thilde Terkelsen, Francesco Russo, Pavel Gromov, Vilde Drageset Haakensen, Søren Brunak, Irina Gromova, Anders Krogh, Elena Papaleo

**Affiliations:** 1 Computational Biology Laboratory, Danish Cancer Society Research Center, Strandboulevarden 49, 2100 Copenhagen, Denmark; 2 Novo Nordisk Foundation Center for Protein Research, Faculty of Health and Medical Sciences, University of Copenhagen, Copenhagen, Denmark; 3 Breast Cancer Biology Group, Genome Integrity Unit, Danish Cancer Society Research Center, Strandboulevarden 49, 2100 Copenhagen, Denmark; 4 Department of Cancer Genetics, Institute for Cancer Research, Oslo University Hospital, Oslo, Norway; 5 Unit of Computational and RNA Biology, Department of Biology, University of Copenhagen, Copenhagen, Denmark

**Keywords:** Tumor interstitial fluid, Breast cancer, Co-expression analysis, Biomarker, Tumor-infiltrating lymphocytes, Tumor grade, TNBC, miRNA families, Gene target, Interaction networks

## Abstract

**Background:**

Studies on tumor-secreted microRNAs point to a functional role of these in cellular communication and reprogramming of the tumor microenvironment. Uptake of tumor-secreted microRNAs by neighboring cells may result in the silencing of mRNA targets and, in turn, modulation of the transcriptome. Studying miRNAs externalized from tumors could improve cancer patient diagnosis and disease monitoring and help to pinpoint which miRNA-gene interactions are central for tumor properties such as invasiveness and metastasis.

**Methods:**

Using a bioinformatics approach, we analyzed the profiles of secreted tumor and normal interstitial fluid (IF) microRNAs, from women with breast cancer (BC). We carried out differential abundance analysis (DAA), to obtain miRNAs, which were enriched or depleted in IFs, from patients with different clinical traits. Subsequently, miRNA family enrichment analysis was performed to assess whether any families were over-represented in the specific sets. We identified dysregulated genes in tumor tissues from the same cohort of patients and constructed weighted gene co-expression networks, to extract sets of co-expressed genes and co-abundant miRNAs. Lastly, we integrated miRNAs and mRNAs to obtain interaction networks and supported our findings using prediction tools and cancer gene databases.

**Results:**

Network analysis showed co-expressed genes and miRNA regulators, associated with tumor lymphocyte infiltration. All of the genes were involved in immune system processes, and many had previously been associated with cancer immunity. A subset of these, *BTLA*, *CXCL13*, *IL7R*, *LAMP3*, and *LTB*, was linked to the presence of tertiary lymphoid structures and high endothelial venules within tumors. Co-abundant tumor interstitial fluid miRNAs within this network, including miR-146a and miR-494, were annotated as negative regulators of immune-stimulatory responses. One co-expression network encompassed differences between BC subtypes. Genes differentially co-expressed between luminal B and triple-negative breast cancer (TNBC) were connected with sphingolipid metabolism and predicted to be co-regulated by miR-23a. Co-expressed genes and TIF miRNAs associated with tumor grade were *BTRC*, *CHST1*, miR-10a/b, miR-107, miR-301a, and miR-454.

**Conclusion:**

Integration of IF miRNAs and mRNAs unveiled networks associated with patient clinicopathological traits, and underlined molecular mechanisms, specific to BC sub-groups. Our results highlight the benefits of an integrative approach to biomarker discovery, placing secreted miRNAs within a biological context.

## Background

Two of the most predictive measures of breast cancer (BC) patient mortality are tumor progression and immune infiltration [1–3]. By decoding and recognizing the underlying molecular patterns of invasive breast tumors, clinicians may provide high-grade tumor patients with appropriate prognosis and treatment, while monitoring the potential progression of lower-grade cancers [4, 5]. Breast tumor invasiveness and patient prognosis are related to molecular subtypes, which are currently classified through PAM50 mRNA expression or immunohistochemistry staining of hormone receptors [6, 7]. BC patients with luminal tumors, defined by the expression of the estrogen and/or progesterone receptor (ER^+|−^, PgR^+|−^), are known to have the best overall outcome [8, 9]. Luminal A type tumors are associated with a slightly better patient survival rate than luminal B tumors, which have high expression levels of Ki-67 (> 14%), and in some cases, *human epidermal growth factor receptor 2* (HER2) amplification [8, 9]. Patients with estrogen- and progesterone receptor-negative (ER^−^, PgR^−^), Her2-amplified tumors, have poorer outcomes than those with luminal subtypes, even though this group of patients has been shown to respond well to targeted therapy [10]. The basal-like and triple-negative breast cancer (TNBC) subtypes, which are largely overlapping and classified by the lack of hormone receptor expression (ER^−^, PgR^−^, Her2^−^) [11], have the poorest prognosis among the subtypes [8, 9].

A precise characterization of the degree of breast tumor invasiveness, alongside the biological relevant pathways and underlying molecular mechanisms, hinges on the identification of a set of specific and sensitive biomarkers.

Recent studies suggest that circulating microRNAs may have great potentials as cancer progression markers [12–14], partially due to their high stability in the plasma/blood [15, 16]. Not only does the level of externalized miRNAs reflects the molecular events underlying tumor progression but, importantly, some studies point to a functional role of tumor-secreted circulating miRNA in intracellular communication and tumor reprogramming [17–19]. Tumor cells may release micro-vesicles into the extracellular space, which may then be taken up by other cells (tumor, epithelial, or immune) via endocytosis [20]. Some micro-vesicles have been found to not only contain mature miRNAs, but pre-miRNAs with accompanying RNA-induced silencing complexes (RISCs) [21]. Uptake of the pre-miRNA exosomes by recipient cells resulted in an efficient silencing of target mRNAs and reprogramming of the cellular transcriptome [22]. In accordance, it has been reported [23] that the release of miRNAs within exosomes was not merely a reflection of the abundance of a given miRNA species, but a selective process facilitated by the tumor cells [23, 24]. For example, exosome-mediated transport of miR-10b from BC cell lines has been shown to promote tumor cell invasiveness in other BC cell lines, which were otherwise not invasive [24].

Circulating miRNAs may also be found free of exosomes, either in complexes with argonaute proteins [25] or bound by high-density lipoprotein (HDL) [26]. HDL-bound circulating miRNAs are delivered to recipient cells, via the scavenger receptor class B/type I-dependent and uptake of these results in targeting of mRNA reporters [26]. MicroRNA silencing of gene targets is facilitated through the interaction of the mRNA 3′ UTR, with the ~ 8 nucleotide seed sequence within the miRNA [27]. Mature miRNAs, which have identical seed sequences, are classified as belonging to the same miRNA family [28]. Because seed sequences of family members are complementary to the same binding motifs, these miRNAs are thought to regulate the same target genes [27, 28]. In a study from 2013, Hamilton et al. [29] identified a pan-cancer oncogenic microRNA family, which was responsible for the co-regulation of central tumor suppressors and, in general, genes functioning within the same pathways. As members of a miRNA family can act as gene co-regulators, it is possible that these are also co-secreted by tumor cells. Studying the relationship between tumor externalized miRNAs should identify which biological processes and pathways are specifically modulated within the tumor cell itself and potentially targeted in recipient cells. Indeed, one very interesting aspect of tumor-secreted miRNA reprogramming is a possible connection to disease progression through the modification of both neighboring and distal tissues. Le et al. [30] showed that exosomes containing miR-200 from metastatic human breast cancer cell lines were absorbed by non-metastatic cells, resulting in the promotion of mesenchymal-to-epithelial transition [30]. The study found that miR-200-expressing tumors used extracellular vesicles to drive metastasis of otherwise weakly metastatic cells at distant sites, providing these cells with the ability to colonize distant tissues in a miR-200-dependent manner [30]. Similarly, it has been reported that cancer cells can suppress glucose uptake by non-tumor cells in the pre-metastatic niche, by secreting micro-vesicles containing miR-122 [31]. Repression of miR-122 restored the glucose uptake in distant organs, while decreasing the incidence of metastasis and disease progression [31].

As such, identification of specifically secreted miRNAs may not only help to improve patient diagnosis/prognosis and disease monitoring, but could also relay information about which target genes are central for particular tumor properties, including invasiveness, and how these properties may be promoted by tumor cell communication.

Despite their potential usefulness, however, identification of robust circulating miRNA biomarkers is no trivial task, as a range of non-cancerous events may cause changes in the levels of biomolecules [32]. Blood-based biomarkers are especially dynamic and can be affected by the time of sampling, patient diet, level of physical activity, medication, and other biological variances, which are extremely difficult to take into account [33]. Furthermore, the serum/plasma may be considered a difficult starting material for marker discovery as cancer-related macro-molecules will be highly diluted and buried in a complex serum/plasma secretome [34, 35].

In recent years, the importance of the tumor microenvironment has become a central area of cancer research, as multiple studies have shown how cancer cells modulate the mechanisms of the surrounding stromal cells in ways that enable the tumor to induce angiogenesis, sustain proliferation, and evade immune destruction [36]. Cross-talk within the tumor stroma is facilitated by the tumor interstitial fluid (TIF), which forms the interface between circulating body fluids [37]. In the local tumor environment, stromal cells and tumor cells are surrounded by TIF, allowing for the secretion and uptake of ions, miRNAs, proteins, and other signaling molecules [38, 39]. As a result, TIF is thought to modulate the epigenetic program of non-malignant cells by tumor cells and vice versa, demonstrating the importance of local tumor milieu for cancer progression [40, 41]. In addition to molecules secreted from tumor and healthy stromal and epithelial cells, TIF encompasses externalized biomolecules from immune cells in the tumor microenvironment [42]. Tumor immune cell infiltration has been shown to be central for the prediction of patient response to treatment and overall survival [2]. The relationship between lymphocyte infiltration and tumor progression is multifaceted [43]. A number of studies have found that a higher degree of CD8+ T lymphocytes is associated with a better outcome for patients with BC [43], especially for the TNBC and Her2-enriched subtypes [44].

In contrast, tumor-infiltrating CD4+ T lymphocytes have been linked to a poorer overall survival. This may be related to the expression of PD-L1 (*programmed death-ligand 1*) by some populations of tumor-infiltrating lymphocytes (TILs), as PD-L1 is a major inhibitor of an anti-tumor immune response [45, 46]. In accordance, the degree of immune infiltration by PD-L1+ T lymphocytes was found to be correlated with large tumors, high-grade tumors, and positive lymph node status [45, 46]. It should be noted that the role of PD-L1 in tumor immune escape is complex, with tumor cells themselves as well as some populations of immune cells displaying this protein, and contributes to anti-immunity in a context-dependent manner [47]. Interstitial fluids provide a snapshot of circulating tumor molecules, as well as immune cell-secreted biomolecules associated with tumor properties such as growth and response to therapy [2, 48]. As the concentration of cancer-specific biomolecules within the local tumor milieu is estimated to be 1000–1500 times that of blood, TIF is a unique resource for BC biomarker identification and a promising alternative to a highly diluted serum secretome [37, 38].

In this study, we analyzed a set of secreted miRNAs from tumor and normal interstitial fluids acquired from 60 women with breast cancer [49]. The availability of clinicopathological information, including tumor grade, receptor status, and BC subtypes classification as well as the characterization of immune infiltration of every biopsy, allowed us to investigate the relationship between interstitial fluid miRNA levels and patient clinical features. We subsequently identified the deregulated gene targets of IF miRNAs in tumor tissues from the same cohort of patients [50]. Integration of miRNA and mRNA expression data helps to pinpoint the perturbed pathways responsible for breast cancer progression while strengthening biomarker selection by utilizing the combinatorial power of a bi-molecular expression profile.

## Materials and methods

### Datasets for analyses

The miRNA and mRNA data analyzed in this study were retrieved from previously published works. Briefly, interstitial fluids had been extracted from surgically resected pieces of breast tumor and normal tissue, collected after mastectomy [49]. The interstitial fluid microRNA dataset from interstitial fluids had been profiled using TaqMan Arrays (TLDA, cat# 4444913; Applied Biosystems, Foster City, CA, USA), as described in [49]. The transcriptome profiles of corresponding breast tumor biopsies were profiled using SurePrint G3 Human GE 8x60K one-color microarrays from Agilent (Agilent Technologies, Cat. No. G4851A); this dataset is published in [50]. The total number of interstitial fluid samples was 60 from tumors and 51 from paired normal fluids, while the tissue mRNA dataset encompassed 96 tumor samples. The two datasets were partially paired, i.e., they were from the same cohort of women with breast cancer; however, not all sample types were available for all patients. For specifics on sample collection, storage, preparation, array types, and protocol, please refer to the primary publications [49, 50].

### Normalization and filtering of tumor interstitial fluid microRNAs

Data were normalized per sample using global normalization, and the abundance of each microRNA was mean-centered. Before analysis, the three samples with technical replicates were averaged. Next, filtering was performed to remove samples with tumor percentages ≤ 40%. In addition, samples with low tumor percentages and one apocrine tumor were excluded. Filtering resulted in the removal of 8 samples (IDs 74, 78, 79, 102, 104, 200, 237, 279). After filtering, the dataset consisted of 51 normal interstitial fluid samples and 52 tumor interstitial fluid samples stratifying into 23 luminal A types, 10 luminal B, 11 Her2-enriched, and 8 triple-negative breast cancer (TNBC) based at the St. Gallen criteria [51].

miRNAs presented in only a small subset of fluids (normal and tumor) were removed. A minimum of 8 TIF samples had to contain a given miRNA, at a level above zero, in order for the miRNA to be retained. The reasoning behind this filtering approach is that some miRNAs may be subtype-specific. Often, a threshold is set so that the minimum of samples containing a given feature corresponds to the size of the smallest group used for comparison (TNBC subtype). Filtering reduced the number of miRNAs from 754 to 561. After filtering miRNA, missing values were substituted with the lowest value observed for a given miRNA over all samples. Abundance values were log2 transformed to deal with extreme values. Log2 transformation resulted in the majority of miRNAs approaching a normal distribution of abundance values. After log2 transformation, the data were corrected for batch effects using the *ComBat* function from the *sva* R package [52]. The batch-corrected data were used only for plotting purposes.

### Normalization and filtering solid breast tumor mRNAs

Before analysis, the two samples with technical replicates were averaged. Next, filtering was performed to remove samples with tumor percentages ≤ 40%. Filtering resulted in the removal of 16 samples. After filtering, the dataset consisted of 80 breast tumor samples stratifying into 35 luminal A types, 11 luminal B, 12 luminal B - Her2-enriched, 9 Her2, 9 TNBC, and 4 unknown/ambiguous subtypes.

Data were normalized and filtered in accordance with the limma guidelines for Single-Channel Agilent Intensity Data (Limma user guide, 15 April 2018, page 112) [53]. Background correction was performed using the “normexp” transformation method [53], followed by between-array normalization of intensities. Only transcripts where nine samples (number of samples in the Her2 group) expressed values above the background level (gIsWellAboveBG) were retained. Filtering reduced the dataset from 62,976 transcripts to a total of 32,767 genes.

### Multidimensional scaling

Classical multidimensional scaling (MDS) (R version 3.3.1) was used for dimensionality reduction of the two datasets: (I) miRNA from interstitial fluids and (II) mRNAs from tumor tissue. MDS was performed with the function *cmdscale*, using Euclidean distance as the distance metric. The plotting was done with R-package *ggplot2 2.2.1* [54]. As the mRNA samples displayed strong array-related batch effects, these data were corrected with *Combat* [52] before clustering.

### *K*-means and hierarchical clustering

Prior to clustering, the R package *Clusgap* [55] was used to estimate the optimal number of clusters (*k*) for *k*-means. *Clusgap* implements the gap statistic, which is a measure of the intra-cluster sum of squares (log (Wk)), or “compactness” of a given clustering [56]. By comparing the pooled within-cluster sum of squares to a null reference distribution, with no obvious clustering, *Clusgap* predicts an optimal *k*—the value for which log (Wk) is minimized compared to the reference distribution. Reference datasets are generated through bootstrapping by sampling randomly with replacement from the original dataset. For the present analysis, a default of 500 bootstraps was used for sampling [55, 56].

Agglomerative hierarchical clustering was performed using the squared Ward distance metric [57].

### Differential abundance analysis of interstitial fluid microRNAs and tumor transcriptome

Differential expression analysis (DEA) was performed using the statistical software *limma* (linear models for microarray data) [53] implemented in R. limma has few underlying statistical assumptions and is known to be powerful for small sample sizes as a result of shrinkage of feature-specific variances [53]. An interstitial fluid miRNA or solid tissue mRNA was considered differentially expressed if the log2 fold change (LogFC) ≥ 1 or ≤ − 1 and the corrected *p* value (FDR) ≤ 0.05.

Differential expression was carried out using the following group comparisons for both datasets: (I) all pairwise subtype combinations, (II) hormone receptor status (ER, PgR, Her2), (III) high TIL status (+ 2|+ 3) vs low TIL status (0|+ 1) and (IV) high-grade tumors (gr 3) vs low/medium-grade tumors (gr 1/2), and (V) *K*-means clusters.

Additionally, a contrast of (VI) tumor interstitial fluids vs normal interstitial fluids was performed for the miRNA set—this was not possible for the solid tissue mRNA, as no normal tissue counterparts had been profiled for mRNA.

As clustering analysis had revealed confounding of tumor immune infiltration scores, tumor grades, and hormone receptor statuses, we tried de-convoluting differentially expressed miRNAs/mRNAs from each contrast by including the other covariates in the design matrix.

In the comparison of TIF vs NIF miRNAs abundance, we added information on sample ID to account for patient tumor heterogeneity. For tumor tissue mRNA contrasts, information on sample array was incorporated into the design matrix to account for this technical variance (batch effect).

### MicroRNA families

Information on miRNA families were obtained from TargetScan v7.2 (http://www.targetscan.org/cgi-bin/targetscan/data_download.vert72.cgi) [58]. miRNA sets from differential abundance analysis (DAA) were integrated with this information in order to identify overrepresented miRNA families in each set. As very few miRNAs belonged to each miRNA family, regardless of set, we had very low power. As a result of this, we did not perform an enrichment test; instead, the number of miRNAs from each set belonging to a specific family were scaled according to the set size and visualized in a tile plot for visual inspection.

### Weighted gene co-expression network analysis (WGCNA)

The *WGCNA* package in R was used to define co-expression modules [59], consisting of genes, or miRNAs, with similar expression patterns. The input of this analysis was the normalized mRNA expression matrix. In the first step, correlations were calculated using the biweight midcorrelation, and a signed weighted correlation network was used to identify co-expression modules with high topological overlap (TO). Modules were defined as branches of a hierarchical cluster tree using the top-down dynamic tree cut method [59]. The expression patterns of each module were summarized by the module eigengene (ME), defined as the first principal component of a given module. Pairs of modules with high module eigengene correlations (*r* > 0.9) were merged. A weighted signed network was computed based on a fit to the scale-free topology. A thresholding power of 9 was chosen (the lowest threshold resulting in a scale-free *R*^2^ fit of 0.85), and the pairwise TO between genes was calculated, which converted pairwise correlation values [− 1,1] to TO co-expression values [0,1] where values close to 1 represented highly shared co-expression neighborhoods. The TO dendrogram was used to define modules using the dynamic tree cut method function in WGCNA [59] with a minimum module size set to 40 genes, deepSplit parameter set to 2 and cutHeight set to 0.99. We used the intramodularConnectivity function from WGCNA to identify module hub genes of interest. This function takes as input the adjacency matrix and the module assignment (i.e., color assignment), giving as the output a measure of intramodular degree. The intramodularConnectivity function computes the whole network connectivity kTotal, the within-module connectivity kWithin, kOut = kTotal-kWithin, and kDiff = kWithin-kOut.

### Paired differentially expressed miRNA and mRNA gene target networks

miRNA target prediction was performed with TargetScan v7.2 [58], using the predicted (conserved) targets of miRNA families (http://www.targetscan.org/vert_72/docs/help.html). Differentially expressed miRNA-mRNA interaction pairs, with opposite expression directionality, were extracted for network construction. In addition to miRNA-mRNA pairs, known direct protein-protein interactions were included from the InBio Map database (https://www.intomics.com/inbio/map.html#downloads) [60], we included mRNA-mRNA (protein-protein) interaction pairs if both mRNAs were differentially expressed in the same comparison.

### Comparison with cancer miRNA databases

Three databases of cancer-related miRNAs were downloaded and curated for comparison in spring 2019: (I) CMEP (Circulating MicroRNA Expression Profiling), http://syslab5.nchu.edu.tw/CMEP/ [61], (II) dbDEMC database of Differentially Expressed miRNAs in human Cancers, http://www.picb.ac.cn/dbDEMC/ [62]; and (III) miRCancer (microRNA Cancer Association Database), http://mircancer.ecu.edu/download.jsp [63]. Information obtained from each of these databases was as follows:

(I). CMEP: the database contains expression levels of miRNAs identified in either blood, serum, or plasma. At the time of download, this database contained 66 cancer studies on circulating miRNAs.
(II). dbDEMC: the database contains miRNAs known to be associated with cancer, based on high-throughput analysis of 209 datasets, from 36 different cancer types and 73 subtypes. miRNAs from this database were quantified from solid tissues, blood, plasma, and serum. At the time of download, 2224 differentially expressed miRNAs were annotated in dbDEMC.
(III). miRCancer: the database contains intracellular miRNA expression profiles from various types of cancers based on PubMed text mining (5700 published studies). At the time of download, miRCancer encompassed 57,984 miRNAs from 196 cancer types, out of which 7325 were identified as differentially expressed.

All miRNAs associated with *breast cance*r were extracted from the three databases, along with the information on the study design, tissue type, and directionality in sample group comparison. MicroRNAs from databases were overlapped with the consensus sets from the TIF miRNA comparisons listed in the “Materials and methods” section. Only miRNAs which were denoted as having the same directionality in a study design comparable to that of the contrast performed our study were kept in the final table. As no miRNAs from the databases were assigned to cancer immune profile, we performed a literature search to obtain a set of comprehensive reviews on immune-related miRNAs, subsequently concatenating these into a list for comparison [64–66].

### Support for miRNA-mRNA pairs

MiRTarBase [67] was used to support TargetScan-predicted miRNA-mRNA pairs. MiRTarBase release 7.0 was downloaded from http://mirtarbase.mbc.nctu.edu.tw/php/download.php. In addition, we supported pairs using a consensus approach, in which we overlapped TargetScan predictions with those from other tools, including DIANA-microT (thermodynamics) [68], miRBridge (complementary, conservation, thermodynamics) [69], PicTar (thermodynamics) [70], PITA (conservation, thermodynamics) [71], rna22 (complementary, conservation) [72], and mirDB (support vector machine) [73]. At least three tools in addition to TargetScan had to return a miRNA-mRNA pair in order for this interaction to be considered “supported.” Methods were implemented though the meta-tool miRsystem [74], except for the results from mirDB, which were added subsequently.

### COSMIC and CancerMine—oncogenes, tumor suppressors, mutational burden, and copy number variations

We employed the COSMIC database [75] and the text mining tool CancerMine [76] to obtain information about differentially co-expressed genes from networks (downloaded: 05-01-2019). Information from COSMIC included (i) single nucleotide polymorphisms (SNPs) within the coding region of genes, annotated as pathogenic by fathmm (Functional Analysis through Hidden Markov Models, v2.3) [77]; (ii) the COSMIC set of quantified copy number variations encompassing whole genes (loss or gain) in breast cancer; and (iii) the COSMIC census set of oncogenes, tumor suppressors genes (TSGs), and dual role genes (DRGs). Additionally, we downloaded genes annotated as oncogenes, TSGs, or driver genes from CancerMine (text mining), both those related to BC, as well as other types of cancer. For the CancerMine dataset, we imposed a cutoff of minimum five citations for any given BC-related gene (75% quantile), while we required at least 12 citations for a non-BC-associated gene to remain in the dataset (90% quantile). Genes from networks were ranked number of copy number variations (loss, gain, and total CNVs were ranked separately) and on mutational burden, here denoted as the number of predicted pathogenic SNPs within the coding region of a gene. All ranks were combined into one final rank for each gene.

To assess a potential enrichment of oncogenes, TSGs, etc. within gene co-expression modules, we performed module-wise Fisher’s exact tests (R-base), with correction for multiple testing using the Benjamini-Hochberg method.

### PubMed search

General terms; [breast cancer], [miRNA/microRNA], [circulating/blood/serum/plasma].

- (V1) terms; [aggressive, aggressiveness, metastasis, metastatic, prognosis, prognostic, invasive, invasiveness, survival].
- (V2) terms; [subtype, subtypes, luminal, Her2, normal-like, ER, PgR, estrogen, progesterone, pam50, immunohistochemistry].

## Results

### *K*-means clustering and dimensionality reduction captured a TNBC profile, tumor grade, and immune infiltration

To investigate whether miRNA abundance patterns could partition NIF and TIF samples, as well as TIF samples from BC patients with different clinical and histological traits, we performed clustering analysis and dimensionality reduction for visualization purposes. Table 1 shows a summary of the sample metadata.

**Table 1:** Sample summary table

	Levels	Number	Percent	Sum %
miRNA	No	37	26.4	26.4
	Yes	103	73.6	100.0
mRNA	No	64	45.7	45.7
	Yes	76	54.3	100.0
Tumor percentage	40	2	1.4	1.4
	60	29	20.7	22.1
	80	58	41.4	63.6
	N	51	36.4	100.0
Grade	1	5	3.6	3.6
	2	46	33.3	36.9
	3	36	26.1	63.0
	N	51	37.0	100.0
Her2	0	23	16.4	16.4
	1+	18	12.9	29.3
	2+	28	20.0	49.3
	3+	20	14.3	63.6
	N	51	36.4	100.0
ER	ER^−^	20	14.3	14.3
	ER^+^	69	49.3	63.6
	N	51	36.4	100.0
PgR	PgR^−^	40	28.6	65.0
	PgR^+^	49	35.0	36.4
	N	51	36.4	100.0
AR	AR^−^	28	20.0	20.0
	AR^+^	57	40.7	60.7
	N	51	39.3	100.0
Sample type	Normal	51	36.4	36.4
	Tumor	89	63.6	100.0
Subtype	Her2-enriched (Her2)	11	7.9	7.9
	Luminal A (LumA)	42	30.0	37.9
	Luminal B (LumB)	13	9.3	47.1
	Luminal B Her2-enriched (LumB-Her2)	13	9.3	56.4
	Triple-negative breast cancer (TNBC)	10	7.1	92.9
	Normal	51	36.4	100.0
TIL score	0	17	12.1	12.1
	1+	25	17.9	30.0
	2+	30	21.4	51.4
	3+	17	12.1	63.6
	N	51	36.4	100.0
Metastasis	0	71	50.7	50.7
	1	18	12.9	63.6
	N	51	36.4	100.0
	**All**	**140**	**100.0**	**100.0**

The letter N denotes normal samples. *miRNA* = TIF miRNA data (yes, no); *mRNA* = tumor tissue mRNA expression data (yes, no); *Tumor percentage* = percentage of tumor tissue in sample, tumor grade (1, 2, or 3); *Her2 =* Her2 receptor status (0, 1, 2, or 3); *ER* = estrogen receptor status (+, −); *PgR =* progesterone receptor status (+, −); *AR =* androgen receptor status (+, −). Sample type (tumor, normal); subtype –luminal A, luminal B, luminal B, Her2-enriched, triple-negative breast cancer, or normal. *TIL score =* tumor-infiltrating lymphocyte score (0, 1, 2, or 3); metastasis—0 is no metastasis and 1 is metastasis; outcome—patient outcome, where 0 is alive and 1 is deceased. The biological characteristics of all samples used were retrieved from [49, 50]

Multidimensional scaling of tumor and normal interstitial miRNA abundance revealed a distinct clustering of the 51 NIF and 52 TIF samples. The first component (M1) highlighted the difference between normal and tumor, while the second component (M2) captured two clusters containing a mix of TIF and NIF samples, Additional file 1: Fig. S1A.

A look into sample IDs revealed that the clustering was most likely driven by patient heterogeneity, a result of a large fraction of samples being paired. As a control for this, we treated “patient” as a covariate and removed this effect, see Additional file 1: Fig. S1B. This correction unified the NIF samples; however, the tumor samples continuously formed two clusters, indicating that TIF sample sub-grouping was driven by similarities between these patients and not merely by overall patient heterogeneity.

**Fig. 1:**
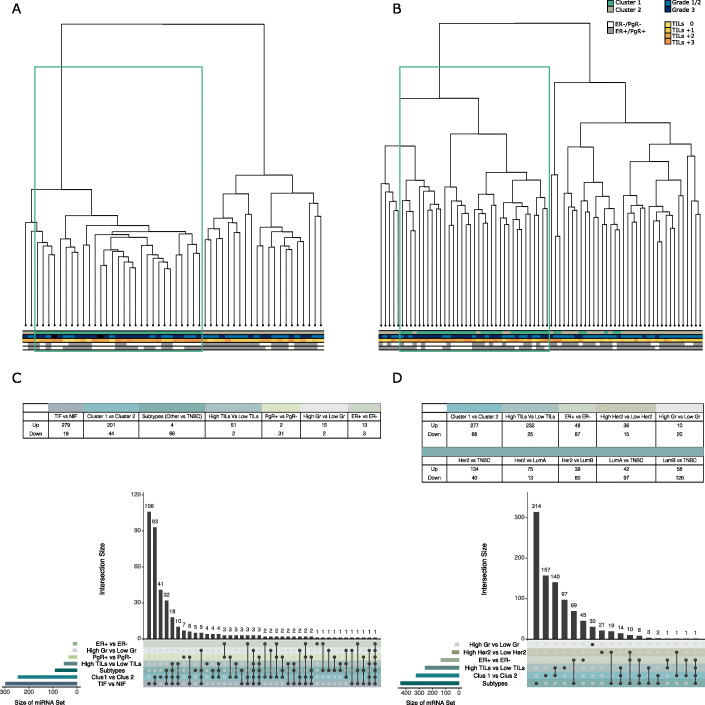
Sample clustering and results of differential abundance/expression analysis. **a**, **b***K*-means clustering of tumor interstitial fluid miRNAs (**a**) and tissue mRNAs (**b**). Both sets display two clusters: cluster 1—hormone receptor-negative samples (ER^−^ and PgR^−^), high grade (grade 3), and high TILs (T2, T3); cluster 2—hormone receptor-positive samples (ER^+^, PgR^+^), lower grade (grades 1 and 2), and low TILs (T0, T1). **c**, **d** Differentially abundant miRNA from normal and tumor interstitial fluids (**c**) and mRNA from tumor biopsies (**d**). Upset plots show the set size for each comparison and redundancy of these miRNAs: up, FDR < 0.05 and logFC > 1; down, FDR < 0.05 and logFC < − 1. Sets: (1) TIF vs NIF, (2) subtypes (luminal A vs TNBC, luminal B vs TNBC, and Her2 vs TNBC), (3) estrogen positive vs negative tumors, (4) progesterone positive vs negative tumors, (5) tumor grade 3 vs tumor grade 1/2, and (6) *K*-means cluster 1 vs cluster 2

As seen in Fig. 1a, miRNA profiles of TIF samples belonging to cluster 1 were predominantly from patients with high-grade (grade 3), high-TIL (tumor-infiltrating lymphocytes, + 2|+ 3) tumors, the majority of which were estrogen- and/or progesterone-receptor negative. In quantitative terms, 75% of samples in cluster 1 were annotated as high grade/TILs. In comparison, TIF cluster 2 was mainly populated by hormone receptor-positive samples with lower grade (grade 1|2) and low TIL scores (TILs 0|+ 1), even though this pattern was less clear than that observed for cluster 1 (62% of samples were low grade/TILs). The correlation of tumor grade and TIL score, albeit not perfect, indicated that tumor interstitial miRNA profiles might be associated with breast cancer progression.

Subsequently, we examined whether mRNA expression patterns from paired tumor tissues yielded a similar clustering and partitioning of samples, as the one observed for interstitial fluid miRNAs. *K*-means (*k* = 2) clustering of tumor mRNA data revealed a pattern comparable to that of the interstitial fluid miRNAs. Tumor mRNA cluster 1 encompassed high-tumor grade, high-TIL samples (89% of high TILs samples and 79% of high-grade samples), while cluster 2 contained samples with lower tumor grade and low-TIL statuses (81% of low TILs samples and 64% of low-grade samples), Fig. 1b. We observed a significantly better partitioning of samples into assigned BC subtype at the mRNA level, as compared to that of TIF miRNA. This was somewhat expected as subtyping is based on the intracellular level of specific mRNA transcripts and/or hormone receptors, Additional file 1: Fig. S1C and S1D. Additional file 2: Table S1 contains information on which samples were assigned to which cluster based on TIF miRNA abundances or intra-tumor mRNA expression levels.

### Expression profiles associated with estrogen receptor status, TNBC subtype, and *K*-means clusters were clear across TIF miRNA and tissue mRNA datasets

We performed differential abundance|expression analysis to identify interstitial fluid miRNAs and solid tissue mRNAs, which were dysregulated in BC samples vs normal samples, and between different BC subgroups. Based on the clustering analysis (Fig. 1a, b), we focused on the following comparisons: (I) TIF vs NIF, (II) BC subtypes, (III) cluster 1 vs cluster 2, (IV) high TILs vs low TILs, (V) ER^+^ vs ER^−^, (VI) PgR^+^ vs PgR^−^, and (VII) high grade vs low/medium grade. Figure 1c and d depict the results of differential expression analysis (DEA) for both interstitial fluid miRNAs (Fig. 1c) and solid tissue mRNAs (Fig. 1d).

#### miRNAs

Approximately 1/3 of miRNAs from the TIF vs NIF set were unique to this comparison, i.e., these interstitial fluid miRNAs may have potentials as BC biomarkers. Another ~ 2/3 of miRNAs differentially abundant between TIF and NIF overlapped both with miRNAs from the TIF cluster 1 vs cluster 2 contrast and the BC subtype contrasts. It should be noted all of the miRNAs from the subtype contrasts were identified between TNBC and the other three subtypes (luminal A, luminal B, and Her2-enriched). The 181 miRNAs identified in the TIF vs NIF comparison, in the subtype comparison and in the cluster 1 vs cluster 2 comparison, likely reflect both a general tumor-specific miRNA pattern, but also the aggressiveness of particular breast cancer subtypes. This observation came from the fact that samples in cluster 1 originated from more advanced tumors (high-grade, high immune score, hormone receptor-negative).

Though there was a large overlap between the aforementioned sets, 41 miRNAs were specific to miRNA cluster 1 vs cluster 2 contrast. There was a large redundancy of miRNAs identified in the contrasts: high TILs vs low TILs, high grade vs lower grade, ER^+^ vs ER^−^, and PgR^+^ vs PgR^−^. MiRNAs, identified as differentially abundant in our analysis, were compared to those obtained in the study by Halvorsen et al. [49] in Additional file 3: Fig. S2 A-C.

#### mRNAs

The comparison of intra-tumor mRNA expression patterns from BC subtypes yielded ~ 450 differentially expressed (DE) genes, out of which 3/4 were unique to the contrast. This was expected, as subtype classification was based on the staining of hormone receptor expression at protein level (immunohistochemistry). The mRNAs not unique to the subtypes contrast nicely overlapped with those identified as DE in the comparisons of estrogen and Her2 receptor amplification (+|−) statuses. No mRNAs were identified as DE between PgR^+^ and PgR^−^, perhaps due to an unbalanced number of samples in each group, in combination with the correction for multiple covariates (i.e., loss of power). A total of 297 mRNAs were identified as differentially expressed between tumor cluster 1 vs cluster 2. Out of these, about half were unique to the cluster comparison, while the other half overlapped with DE mRNAs from the high TILs vs low TILs comparison, indicating that these mRNAs may drive the immune-related profile observed in Fig. 1b. Surprisingly, the set of mRNAs which were DE between high- and medium/lower-grade tumors did not overlap the tumor cluster set, and TILs set but appeared to be unique to this contrast.

Collectively, the analysis resulted in sets of differentially abundant TIF miRNA, and comparison matched mRNA sets for (I) high TILs vs low TILs, (II) high grade vs medium/low grade, (III) cluster 1 vs cluster 2, (IV) ER^+^ vs ER^−^, and (V) luminal A|B and Her2 vs TNBC.

### Expression sets from clusters (tumor grade, immune infiltration) were enriched for microRNA families: miR-15, miR-17, and miR-130

miRNAs with identical seed regions could potentially bind and silence the same gene targets [29, 78]. Also, genes with shared functions may have the same miRNA binding sites, so these can be conjointly regulated by specific mRNA families [79]. As such, the identification of miRNA families enriched or depleted in tumor samples may increase our understanding of which gene functions and pathways are dysregulated in cancer.

**Fig. 2:**
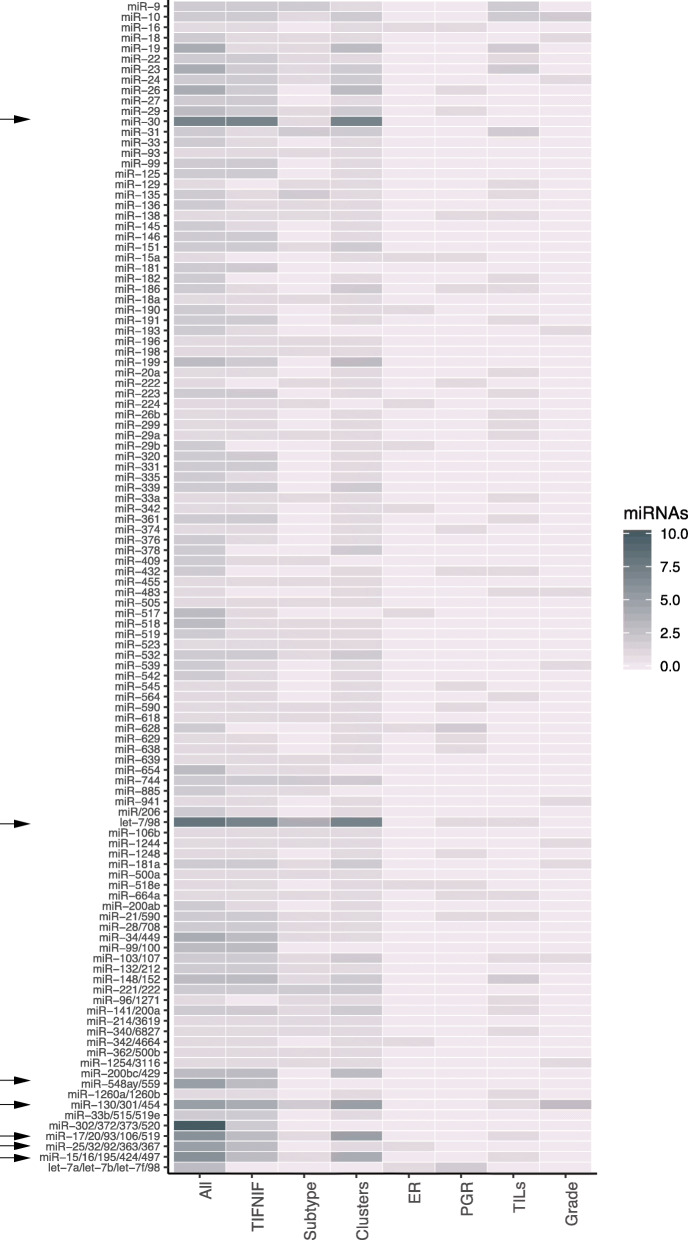
MicroRNA family enrichment. Sets of differentially abundant miRNA assigned to miRNA families. *x*-axis = sets of DA interstitial fluid miRNAs. *y*-axis = miRNA family. Color = number of miRNAs belonging to a given family in set. Darker color = more miRNAs belonging to this family. Arrows = miRNAs of interest

We mapped miRNAs from each DA set to their respective families, highlighting any potential differential abundance of related miRNAs, see Fig. 2.

Common to the TIF-NIF, subtype, and cluster sets were miRNAs belonging to the let-7/miR-98 microRNA family, known to be aberrantly expressed in a range of cancer types [80] including breast cancer [81, 82]. While a few miRNA families were generally abundant in all sets, some were more set-specific. For TIF cluster 1 vs cluster 2, miRNA members from the miR-15 family (miR-15, miR-16, miR-195, miR-424, miR-497), miR-17 family (miR-17, miR-20, miR-93, miR-106, miR-519), and miR-130 family (miR-130, miR-301, miR-454) were predominant. Collectively, the enrichment of the miRNA families supports the partitioning of TIF samples into two clusters, representing differential tumor progression.

While families miR-15, miR-17, and miR-130 were most prevalent in TIF cluster sets, miR-30 and miR-200bc were shared between this set and the TIF vs NIF set. More specific to the TIF vs NIF comparison were two miR families: miR-25/miR-92 and miR-548ay/559. The miR-25/miR-92 family members are situated in the *mir106a-363* and *mir106b-25* clusters (Chr X and Chr 7, respectively), paralogs to the polycistronic miRNA cluster *mir-17-92*, also designated *oncomir-1* [83]. miR-25 and miR-92a have been proposed to be negative regulators of tumor cell apoptosis by directly targeting Bim (Bcl-2-interacting mediator of cell death) [84].

### Dataset integration revealed BC-related miRNA-mRNA interaction pairs related to clinicopathological information

To further explore the interplay between circulating miRNAs and their potential intracellular gene targets, we constructed custom networks from the differential abundance|expression sets (see the “Materials and methods” section). Table 2 shows the total number of identified DA TIF miRNAs and DE intracellular mRNAs before and after “pairing” into the interaction networks (direct miRNA target genes + gene partners).

**Table 2:** Summary Table showing number of DE tumor interstitial fluid miRNAs and mRNAs, before and after pairing

	HER2 vs TNBC	LumA vs TNBC	LumB vs TNBC	Cluster 1 vs Cluster 2	ER+ vs ER-	High TILs vs Low TILs	High Grade vs Low/Medium Grade
**DE miRNAs**	90	90	90	245	16	53	17
**DE mRNAs**	174	139	184	343	135	257	30
**Retained DE miRNAs**	31	29	31	89	4	17	7
**Retained DE mRNAs**	27	23	34	113	19	65	5
**Pairs**	88	74	96	412	19	156	9

For the genes included in each interaction network, we extracted information about the frequency of mutation (SNPs predicted to be pathogenic) and copy number variations (CNVs) from the COSMIC database [75], as well as information about the gene role in cancer from COSMIC (census set) and CancerMine [76]. Genes were subsequently ranked on these parameters, allowing us to evaluate the potential known (well-supported) role, or novelty, of a gene candidate within a (breast) cancer setting—see Additional file 4: Table S2 for all gene-wise information.

#### Tumor immune infiltration

**Fig. 3:**
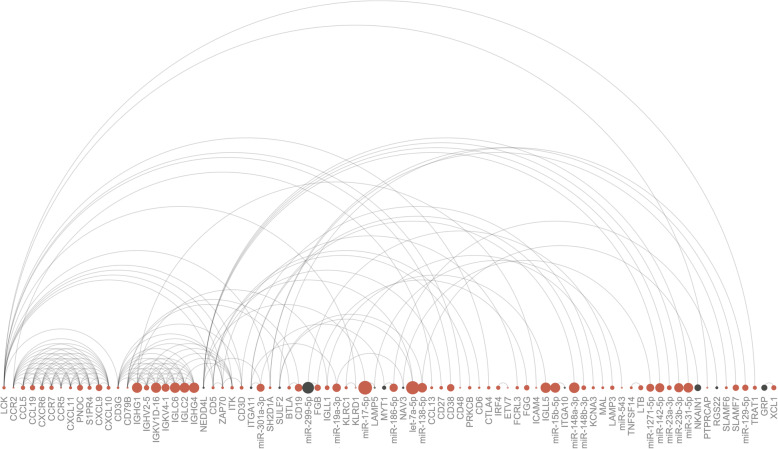
miRNA-mRNA interaction plot. Interaction network for DE miRNA-mRNA targets from the comparison of high TILs vs low TILs. The size of a dot denotes the absolute log2 fold change. Colors refer to the expression directionality, red = upregulated and black = downregulated. Networks for all comparison may be found in Additional file: Fig. S3

Figure 3 shows the interaction network generated from miRNAs and gene targets differentially expressed in the comparison of high TILs vs low TILs—network plots for all other comparisons may be seen in Additional file 5: Fig. S3. Figure 3 shows that the differentially expressed mRNAs from high TIL vs low TIL samples were mainly chemokines, immunoglobulins, and T cell differentiation antigens, along with other genes related to immune processes (*BTLA*, *ITK*, *ZAP70*, *SLAMF6/7*) [85]. This observation was confirmed by pathway enrichment analysis, which returned as top pathways: *cytokine-cytokine receptor interaction*, *chemokine signaling pathway*, and *NF-kappa B signaling pathway*, both with and without genes which were DE in cluster 1 vs cluster 2 (e.g., redundancy removed). The most interconnected gene was LCK, which is a well-known oncogene in T cell acute lymphoblastic leukemia supported by COSMIC and CancerMine, Additional file 4: Table S2. The interconnectivity of this gene underlined that this miRNA-mRNA network was associated with tumor immune cell infiltration and gene expression. While LCK had the highest number of interactions, the gene with the most miRNA partners was NEDD4L, which was downregulated in the high TIL vs low TIL comparison. Interestingly, the NEDD4L gene had a high rank (nr. 3) based on mutational burden and CNVs, and this gene has been proposed to be a TSG in studies on breast and liver cancer [86, 87]. miR-301-3p was the miRNA from this network with most gene targets, including NEDD4L, MYT1 (ranked nr. 2 based on CNVs and SNPs), and ITGA11.

#### *K*-means clusters

There was a significant overlap of DE miRNA-mRNA pairs retained in the high TILs vs low TILs and the cluster 1 vs cluster 2 comparisons. This was not unexpected as cluster 1 contained high TILs tumors/TIFs, while cluster 2 contained low TILs tumors/TIFs. In accordance with this, the overlap mainly encompassed immune-related genes, including chemokines, immunoglobulins, and lymphocyte antigens [88–90], 45 in total. Additionally, 15 miRNAs were shared by these two contrasts, here among miR-301—see Additional file 6: Table S3.

Despite the overlap, some miRNAs and mRNAs were specific to each comparison, especially for the cluster set, which was large with many pairs. For the TILs set, only miR-543 was unique. miR-543 interacts with *LAMP5* (*lysosome-associated membrane protein*), very recently shown to be an autophagy suppressor that protects leukemia fusion oncoproteins, helping these to evade degradation [91]. A total of 51 mRNAs and 65 genes were unique to the cluster 1 vs cluster 2 comparison—Additional file 6: Table S3. Just as for the analysis with TIL scores, pathway enrichment analysis with miRNA-mRNA gene pairs DE from the contrast with clusters were enriched within the *cytokine-cytokine receptor interaction pathway*, *chemokine signaling pathway*, and *NF-kappa B signaling pathway*. In addition to this, however, the genes from the cluster 1 vs cluster 2 comparison were also enriched within *pathways in cancer*, indicating that cluster comparison captured more than the tumor immune signature. The most interconnected gene in the cluster comparison was PARD6B, which had a high ranking based on the number of copy number gains; however, this gene was not annotated as an oncogene or TSG, neither the COSMIC census nor from text mining—Additional file 5: Fig. S3 and Additional file 4: Table S2. PARD6B was downregulated in higher-grade, high-TIL, hormone receptor-negative BC samples. The miRNAs with most gene targets were miR-494-3p and miR-103a-3p both of which were downregulated in the higher-grade, high-TIL, hormone receptor-negative samples. miR-494-3p was mainly paired with immune genes (CD3G, CXCL13, CXCR5, KLRC4), some of which had been annotated as oncogenes in leukemia including IGF1 (DRG; pan-cancer), IKZF3 (driver; leukemia), NABP1 (oncogene; leukemia), and PDGFRA (oncogene; pan-cancer). miR-103a-3p interacted with genes SEL1L3, BTLA, LDLRAD2, PDE3B, and PKIA. The network is in Additional file 5: Fig. S3 A.

#### Tumor grade

The network of IF miRNAs and mRNAs from the high-grade (grade 3) vs the lower-grade (grade 1|2) biopsies was small with only five genes and six miRNAs. Two genes *BTRC* and *CHST1* were downregulated in high-grade tumors and interacted with miRNAs miR-10(a/b)-3p, miR-107, and miR-301(a/b)-3p, miR-454-3p, respectively. miR-18a-5p was paired with genes: *AMIGO1*, *KCND3*, and *SIK3* and was upregulated in grade 3 vs grade 1|2 tumors. The network is in Additional file 5: Fig. S3 c.

#### BC subtypes and estrogen receptor

Networks of differentially abundant IF miRNAs and mRNAs from the contrasts with Her2-enriched vs TNBC, luminal A vs TNBC, and luminal B vs TNBC shared eight gene transcripts. These were: *AR* (oncogene; prostate cancer), *CERS6*, *FOXA1* (oncogene; breast and prostate cancer), *GPR160*, *KIAA1244*, *KLK5*, *SPDEF* (DRG; breast, prostate, lung, and colon cancer), and *XBP1* (oncogene; blood, esophageal, and brain cancer), all of which have been shown to be BC-related and differentially expressed between subtypes [92–96]. A total of 27 miRNAs were shared between the three networks, not surprising as the set of DE TIF miRNAs identified in each comparison was almost completely redundant. The most interconnected miRNAs of these were miR-9-5p, miR-15b-5p, miR-17-5p, miR-19a-3p, and miR-30d-5p, downregulated in all three subtypes compared to TNBC.

While some miRNAs and mRNAs were shared by all three DE networks, some were specific, or partially so, to each comparison. As expected, the most interconnected gene in both luminal types compared to TNBC was *ESR1* [97] (DRG; breast, liver, nasopharynx, kidney, lung, bone, endometrial, and prostate cancer), and in addition, genes KIF3B, KRT4, and NFIB (DRG; breast, lung, glandular, leukemia, bone, skin, brain cancer) were shared between these two networks. KIF3B was upregulated in the luminal types, while KRT4 and NFIB (gene ranked nr. 1, based on mutations and CNVs) were downregulated. Specific to the luminal A comparison was the gene CCND1 (oncogene; breast and pan-cancer), the second most interconnected after ESR1 (oncogene; breast and pan-cancer), while ELOVL6 (oncogene; liver cancer) was the most interconnected unique gene within the luminal B vs TNBC network. Networks are in Additional file 5: Fig. S3 E,F.

For the contrast of Her2-enriched vs TNBC subtypes, the gene with the most interactions was CPD, closely followed by genes ERBB2 (oncogene, breast, and pan-cancer), GRB7 (oncogene; breast cancer), and LASP1 (DRG; liver, esophageal, leukemia, brain, thyroid gland, lung, and stomach cancer), all of which belong to the Her2 amplicon (chromosome region 17q-12-21) [98]. These DE genes were ranked highest based on the number of predicted pathogenic SNPs and CNVs from COSMIC datasets. The ER+ vs ER^−^ network had miRNA-mRNA pairs that overlapped with those from the luminal vs TNBC networks. The most interconnected genes were ESR1, GATA3 (DRG; breast, stomach, prostate, colorectal, lung cancer), and GREB1, all upregulated in ER+ samples, while ERBB2 which was downregulated. Unique to this comparison was miR-32-5p, which was over-expressed in ER+ vs ER^−^ tumors and the most interconnected miRNA in the network.

Networks may be seen in Additional file 5: Fig. S3 D,G. Lists of DE mRNAs and interstitial fluid miRNAs from pairs may be found in Additional file 6: Table S3.2 (common across sets) and Additional file 6: Table S3.1 (unique to sets).

Network analysis resulted in multiple miRNA-mRNA pairs, where both TIF miRNA and intracellular mRNA profiles displayed meaningful directionality in accordance with previously published studies and in the context of a given comparison.

### Cancer miRNA databases—support for TIF miRNAs as potential BC biomarkers

Three databases of cancer-related miRNAs were downloaded and curated in order to compare and support the results obtained from the analysis of interstitial fluid miRNAs. These databases included (I) CMEP [61] (Circulating MicroRNA Expression Profiling), (II) dbDEMC database of (Differentially Expressed miRNAs in human Cancers) [62], and (III) miRCancer (microRNA Cancer Association Database) [63]. Briefly, the CMEP database contains circulating miRNA from the blood, plasma, and serum, while the dbDEMC database and miRCancer contain both circulating and intracellular miRNAs. MiRCancer is based on PubMed text mining (e.g., miRNAs only have assigned directionality), while CMEP contains raw data and dbDEMC contains log fold changes of DE miRNAs. As none of the miRNAs from databases was assigned to cancer immune profile, we performed a literature search to obtain a set of comprehensive reviews on immune-related miRNAs, subsequently concatenating these into a list for comparison [64–66].

**Fig. 4:**
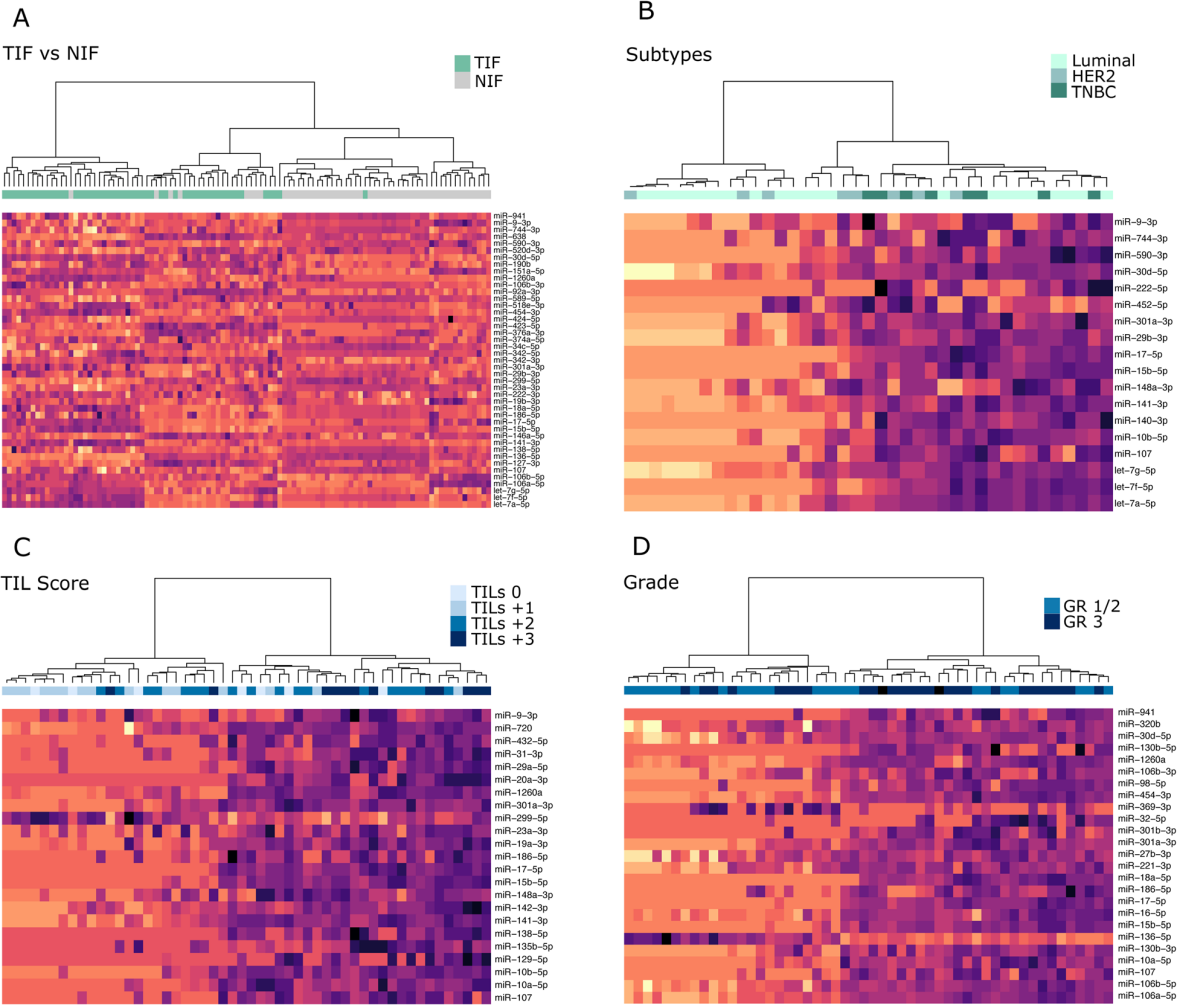
Heatmaps of DA miRNA levels in TIF. Heatmaps showing the separation of TIF samples based on the best DE miRNA candidates from comparison with databases. **a** TIF vs NIF. **b** Luminal (**a**, **b**), Her2-enriched vs TNBC. **c** TIL scores. **d** Tumor grade. Color scale denotes the expression levels, purple = high expression and yellow = low expression

Table 3 shows the best supported differentially abundant interstitial fluid miRNAs from the different comparisons. Each miRNA was included in at least one of the three databases, with a consensus of expression directionality, and were among the most interconnected miRNAs from the custom miRNA-mRNA networks. As no networks could be constructed for the TIF vs NIF and PgR^+^ vs PgR^−^ contrast (see the section above), miRNAs from these sets were only supported by overlap with databases. Figure 4a–d shows the partitioning of samples based on the top best-supported miRNAs from comparisons: TIF vs NIF, luminal/Her2-enriched vs TNBC, high TILs vs low TILs, and high-grade vs low/medium grade. miRNA candidates from the ER^+^ vs ER^−^ comparison were encompassed by the luminal/Her2-enriched vs TNBC. As seen from the heatmaps in Fig. 4, there was a good concordance between the expression of IF miRNAs in the TIF vs NIF, TILs ,and grade comparisons (Fig. 4a, c, d). For the luminal/Her2-enriched vs TNBC, this pattern was poorer with come separation of luminal from TNBC and Her2-enriched samples, but not between these two subtypes.

**Table 3:** Best supported circulating differentially expressed miRNAs based on overlap with databases (CMEP, dbDEMC and miRCancer) and miRNA-mRNA interactions networks

***miRNA***	***BC vs Normal***	***Luminal/HER2 vs TNBC***	***ER+ vs ER-***	***PgR+ vs PgR-***	***High TILs vs Low TILs***	***High TILs vs Low TILs & High Gr vs Low/Medium Gr***	***High Gr vs Low/Medium Gr***	***Metastasis***	***Poor Outcome***
hsa-let-7a-5p	Up	Down	.	.	.	.	.	.	.
hsa-let-7f-5p	Up	Down	.	.	.	.	.	.	.
hsa-let-7g-5p	Up	Down	.	.	.	.	.	.	.
hsa-miR-103a-3p	Down	.	.	.	.	Down	.	.	.
hsa-miR-106a-5p	Up	.	.	.	.	Up	.	Up	.
hsa-miR-106b-3p	Up	.	.	.	.	Up	.	Up	.
hsa-miR-106b-5p	Up	.	.	.	.	Up	.	Up	.
hsa-miR-107	Up	Down	.	.	Up	Up	Up	Up	.
hsa-miR-1260a	Up	.	.	.	Up	Up	.	Up	.
hsa-miR-127-3p	Up	.	.	.	.	.	.	.	.
hsa-miR-136-5p	Down	.	.	.	.	Down	.	Down	.
hsa-miR-138-5p	Up	.	.	.	Up	Up	.	.	.
hsa-miR-141-3p	Up	Down	.	.	Up	Up	.	Up	.
hsa-miR-146a-5p	Up	.	.	.	.	Down	.	.	.
hsa-miR-151a-5p	Up	.	.	.	.	.	.	.	.
hsa-miR-15b-5p	Up	Down	.	.	Up	Up	.	Up	.
hsa-miR-17-5p	Up	Down	.	.	Up	Up	.	Up	.
hsa-miR-186-5p	Up	.	.	.	Up	Up	.	Up	.
hsa-miR-18a-5p	Up	.	.	.	.	Up	Up	.	.
hsa-miR-190b	Up	.	Up	.	.	Up	.	.	.
hsa-miR-19b-3p	Up	.	.	.	.	Up	.	.	.
hsa-miR-222-3p	Up	.	.	.	.	Up	.	.	.
hsa-miR-23a-3p	Up	.	.	.	Up	Up	.	.	.
hsa-miR-299-5p	Down	.	.	.	Down	Down	.	Down	.
hsa-miR-29b-3p	Up	Down	.	.	.	.	.	.	.
hsa-miR-301a-3p	Up	Down	.	.	Up	Up	Up	Up	Up
hsa-miR-30d-5p	Up	Down	.	.	.	Up	.	Up	.
hsa-miR-342-3p	Up	.	Up	.	.	.	.	.	.
hsa-miR-342-5p	Up	.	Up	.	.	.	.	.	.
hsa-miR-34c-5p	Up	.	.	.	.	.	.	.	.
hsa-miR-374a-5p	Up	.	.	Down	.	.	.	.	.
hsa-miR-376a-3p	Up	.	.	.	.	Down	.	.	.
hsa-miR-423-5p	Up	.	.	.	.	.	.	.	.
hsa-miR-424-5p	Up	.	.	.	.	Up	.	.	.
hsa-miR-454-3p	Up	.	.	.	.	Up	Up	Up	.
hsa-miR-494-3p	Down	Up	.	.	.	Down	.	Down	.
hsa-miR-518e-3p	Up	.	Up	Down	.	.	.	.	.
hsa-miR-520d-3p	Up	.	.	.	.	.	.	.	.
hsa-miR-589-5p	Up	.	.	.	.	.	.	.	.
hsa-miR-590-3p	Up	Down	.	Down	.	.	.	.	.
hsa-miR-638	Up	.	.	Up	.	.	.	.	.
hsa-miR-744-3p	Up	Down	.	.	.	.	.	.	.
hsa-miR-9-3p	Up	Down	.	.	Up	.	.	.	.
hsa-miR-92a-3p	Up	.	.	.	.	.	.	.	.
hsa-miR-941	Up	.	.	.	.	Up	Up	Up	Up
hsa-miR-10b-5p	.	Down	.	.	Up	Up	.	.	.
hsa-miR-140-3p	.	Down	.	Down	.	.	.	.	.
hsa-miR-148a-3p	.	Down	.	.	Up	Up	.	.	Up
hsa-miR-222-5p	.	Down	.	Down	.	.	.	.	.
hsa-miR-452-5p	.	Down	Down	.	.	.	.	.	.
hsa-miR-29b-2-5p	.	.	Up	.	.	.	.	.	.
hsa-miR-32-5p	.	.	Up	.	.	Up	.	Up	Up
hsa-miR-922	.	.	Up	Down	.	.	.	.	.
hsa-miR-126-3p	.	.	.	Down	.	.	.	.	.
hsa-miR-29c-3p	.	.	.	Down	.	.	.	.	.
hsa-miR-432-3p	.	.	.	Down	.	.	.	.	.
hsa-miR-450b-5p	.	.	.	Down	.	.	.	.	.
hsa-miR-629-5p	.	.	.	Down	.	.	.	.	.
hsa-miR-10a-5p	.	.	.	.	Up	Up	Up	.	.
hsa-miR-129-5p	.	.	.	.	Up	.	.	.	.
hsa-miR-149-3p	.	.	.	.	Up	.	.	.	.
hsa-miR-182-3p	.	.	.	.	Up	.	.	.	.
hsa-miR-19a-3p	.	.	.	.	Up	Up	.	.	.
hsa-miR-432-5p	.	.	.	.	Up	Up	.	.	.
hsa-miR-720	.	.	.	.	Up	.	.	.	.
hsa-miR-130b-3p	.	.	.	.	.	Up	.	Up	Up
hsa-miR-130b-5p	.	.	.	.	.	Up	.	Up	.
hsa-miR-135a-5p	.	.	.	.	.	Up	.	.	.
hsa-miR-16-5p	.	.	.	.	.	Up	.	Up	.
hsa-miR-20a-5p	.	.	.	.	.	Up	.	.	.
hsa-miR-20b-5p	.	.	.	.	.	Up	.	.	.
hsa-miR-301b-3p	.	.	.	.	.	Up	Up	Up	.
hsa-miR-30b-5p	.	.	.	.	.	Up	.	.	.
hsa-miR-369-3p	.	.	.	.	.	Down	.	Down	.
hsa-miR-874	.	.	.	.	.	Down	.	.	.

Collectively, the best supported interstitial fluid miRNAs, from differential expression analysis, miRNA-mRNA interaction networks, and miRNA databases (circulating and solid tissue), could partition normal fluids and tumor fluids from BC patients with different TIL scores and tumor grade.

### Gene co-expression analysis—highly interconnected genes from modules were lncRNAs

We applied WGCNA to our set of mRNA in order to uncover genes with similar expression patterns. Sets of co-expressed genes could have similar functions and may belong to the same pathways and cascades involved in the development of breast cancer.

WGCNA analysis of mRNA expression data revealed 31 modules encompassing a different number of genes. We explored the higher-level organization and the relationship between these modules by applying hierarchical clustering, using the correlation of the module eigengenes as input (distance metric). The resulting group of modules is sets of positively correlated eigengenes, which may serve as the input for future exploratory analyses, generating new interesting hypotheses (Additional file 7: Fig. S4).

Additionally, we performed intramodular connectivity analysis for each module, which measures how connected, or co-expressed, a given gene is with respect to the other genes within a particular module (Additional file 8: Table S4). Briefly, this analysis showed that the turquoise and brown modules encompassed genes with the highest kWithin, which was partially a consequence of the high density of these two networks. Interestingly, several of these genes belonged to a class of long non-coding RNAs (lncRNAs) with unknown functions. LncRNAs could be important hubs within these networks, regulating crucial mechanisms (e.g., interactions with proteins or miRNAs), and may be involved in the pathogenesis of breast cancer. To date, the inference of the biological roles of lncRNAs in cancer development remains a challenge, but increasing attention has been given to these molecules considered as potential key players in [99]. Novel computational approaches and resources are now available to help researchers in the interpretation of the functions of lncRNAs [100–102]. However, many lncRNAs in our results are still poorly annotated. Future developments in this field will allow us to understand the pathogenesis of breast cancer involving complex regulatory networks consisting of lncRNAs, mRNAs, miRNAs, and proteins. In this context, the knock-down of the lncRNA hubs could potentially have significant effects on the stability of the modules, resulting in the partial or complete rewiring of the networks.

### Co-expression modules were correlated with subtype, immune infiltration, and grade

Modules were correlated with clinical features including hormone receptor status (ER and PgR), BC subtype, immune infiltration scores, tumor grade, and metastasis information, connecting co-expressed genes with the clinical metadata, see Additional file 9: Fig. S5.

Out of the 31 modules identified, six were correlated with the clinical variables. The green and red modules showed a positive correlation with estrogen receptor status/luminal subtypes and a negative correlation with TIF clusters, respectively, characterized by tumor grades and TIL scores. Inversely, the yellow, green, and grey60 modules were positively correlated with TIF clusters, TIL status, and tumor grade, respectively, and negatively correlated with estrogen receptor status/luminal subtypes. The blue module was positively correlated with tumor grade (and metastasis) only. Based on these observations, we intersected differentially expressed mRNAs from each comparison with the respective modules of interest to see which of these, and how many, were retained in each module. Additional file 10: Fig. S6 shows the Venn diagrams of each set overlap. We extracted DE co-expressed genes from the six modules of interest and visualized these in heatmaps with the relevant clinical information—Additional file 11: Fig. S7. As seen from the heatmaps in Fig. S7, co-expressed genes from modules, which were also differentially expressed, yielded adequate segregation of BC subtypes, estrogen receptor status, degree of TILs, and, to a lesser extent, tumor grade.

**Fig. 5:**
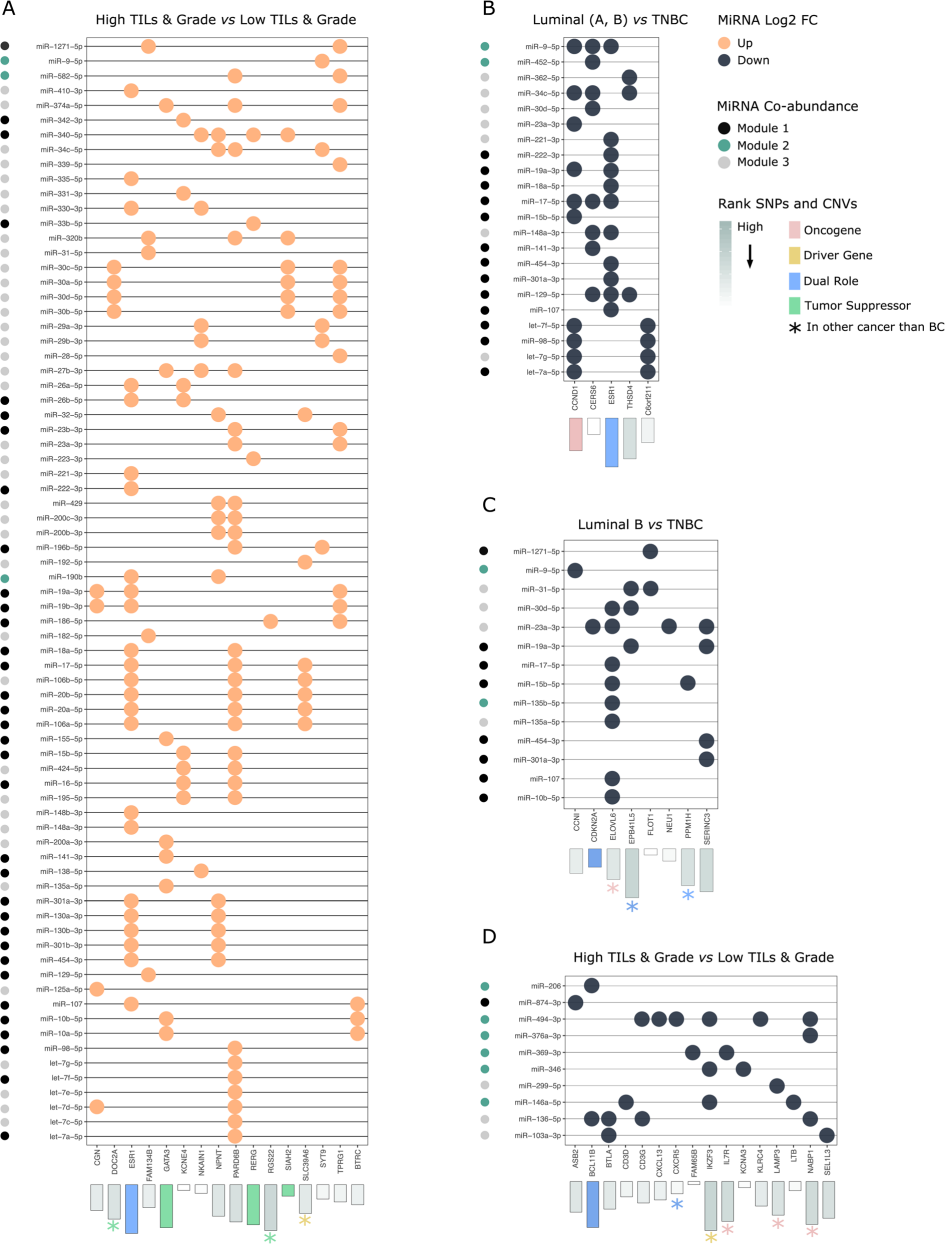
Co-abundant miRNA-mRNA pairs. Dot plots showing the differentially co-expressed genes and their predicted co-abundant miRNA regulators, for the green, yellow, and red modules. Colors: light orange = TIF miRNA upregulated in given comparison, dark blue = TIF miRNA downregulated in given comparison; N. B genes have the inverse direction of expression as miRNAs. Bar plots relay information about genes extracted from the COSMIC database [75]. Height/shade of the bar indicates gene rank, based on the mutational burden (SNPs classified as pathogenic) and copy number variations (CNVs, grain + loss) of a gene from breast tumors. Colors of the bars denote if the gene was classified as a BC oncogene, a BC driver gene, a BC tumor suppressor gene, or a BC dual role gene within the set of COSMIC gene census set [75] and/or from CancerMine [76]. A star means that a gene was annotated in COSMIC or CancerMine for another cancer than BC, and the color indicates the gene role. Smaller dots adjacent to miRNA name denote miRNA co-abundance modules (modules 1–3). Shades: black = module 1, green = module 2, and gray = module 3

As we were specifically interested in TIF miRNAs, which could potentially regulate the expression of cancer-related genes through intracellular cross-talk, we extracted the DE miRNA-gene target interactions of those genes, both DE and co-expressed in the modules of interest. These miRNA-gene pairs were filtered to only retain pairs where the miRNA was supported by one or more databases (see the section above). Overall, this data “curation” resulted in a small handful of genes interacting with multiple miRNAs, many of which were redundant between comparisons. The comparisons with miRNA-gene pairs from *K*-means clusters and subtypes retained most pairs. Genes in these pairs belonged either to the green module, red module, or yellow module—see Fig. 5.

#### Yellow module

The analysis revealed that the yellow module (Fig. 5d) consisted of a cluster of co-expressed immune system genes [85, 88–90], regulated by a small set of miRNAs. Gene targets were upregulated, while all miRNAs were downregulated in high TILs vs low TILs. Gene transcripts included, *ASB2*, *BCL11B*, *BTLA*, *CD3D*, *CD3G*, *CXCL13*, *CXCR5*, *FAM65B*, *IKZF3*, *IL7R*, *KCNA3*, *KLRC4*, *LAMP3*, and *LTB*, mainly interacting with miR-146a-5p and miR-494-3p. We observed miR-146a and miR-494 to be downregulated in TIFs from high-grade tumors with high TIL scores. All miRNAs from the module, except miR-346, were annotated in the CMEP database as differentially expressed in the blood from BC patients. miR-103a, miR-494, and miR-369 were downregulated in TNBC compared to other subtypes, while miR-206, miR-299, and miR-874 were downregulated in relapse, metastasis, and stage 3 vs stage 2 cancers, respectively. As no serum set from CMEP was specifically related to immune status, we could not perform this check; however, the directionality of these miRNAs was somewhat consistent with our results, as high immune score tumors were mainly high-grade TNBC. A search through the STRING database [103] with the set of upregulated genes, revealed these to form a network, which was enriched in immune processes and pathways. Out of the genes within this network, only BCL11B was annotated in the COSMIC census set of genes with known roles in BC. However, some of the other genes in the network had been denoted as oncogenes/driver genes in relation to different blood cancers (leukemia, lymphoma, or myeloma). This is in line with the fact that these genes were differentially co-expressed between breast tumors with different levels of immune infiltration.

#### Red module

The red module (Fig. 5c) contained co-expressed genes *CCNI*, *CDKN2AIPNL*, *ELOVL6*, *EPB41L5*, *FLOT1*, *NEU1*, *PPM1H*, and *SERINC3*, with miRNA partners almost equally distributed across these. miR-23a had the highest number of assigned gene partners, four in total: *CDKN2AIPNL*, *ELOVL6*, *NEU1*, and *SERINC3*. A look into these genes reveals that with the exception of *CCNI*, these genes were upregulated in luminal B vs TNBC comparison exclusively. Cross-reference of miR-23a with the CMEP database of circulating miRNAs showed this miRNA to be differentially expressed in the blood from patients with Her2-enriched cancers compared to TNBC. STRING enrichment analysis of the red co-expression module revealed genes *ELOVL6*, *NEU1*, and *SERINC3* to belong to the sphingolipid metabolic process.

The genes in this network were not well-annotated in terms of their role in breast cancer. However, EPB41L5 and SERINC3 had a high rank based on mutational burden and CNVs, ELOVL6 was annotated as an oncogene in prostate cancer, and PPM1H has been proposed to be an oncogene, due to its relation to PPM1D (a well-studied oncogene in breast, ovarian, and brain cancers).

#### Green module

Co-expressed genes from this module and the miRNAs that regulate them were confounded, on the one hand originating from the high-grade vs high-TIL set and from the comparison of TNBC vs other subtypes. This is supported by Fig. 5, as many miRNAs that were upregulated in the comparison of high TILs|grade vs low TILs|grade (Fig. 5a) were, inversely, downregulated in the luminal/Her2 vs TNBC comparison (Fig. 5b). Co-expressed gene transcripts in this module, associated with the luminal vs TNBC set, were *C6orf211*, *CCND1*, *CERS6*, *ESR1*, and *THSD4*. Enrichment and pathway analysis of the full set of genes related to BC subtypes revealed that four of these *CERS6*, *ELOVL6*, *NEU1*, *and SERINC3* were involved in sphingolipid metabolism, whereas *C6orf211 (ARMT1)*, *CCND1*, and *ESR1* were annotated in the breast cancer KEGG pathway.

Co-expressed DE gene transcripts from the green module associated with TIF miRNAs from the TILs/tumor-grade comparison were *BTRC CGN*, *DOC2A*, *ESR1*, *FAM134B*, *GATA3*, *KCNE4*, *NEDD4L*, *NKAIN1*, *NPNT*, *PARD6B*, *RERG*, *RGS22*, *SIAH2*, *SLC39A6*, *SYT9*, and *TPRG1*. This set of co-expressed genes were regulated by a range of TIF miRNAs, most of which paired with between 2 and 3 genes each, with miR-340-5p as the only miRNA interacting with four transcripts: *NKAIN1*, *NPNT*, *RERG*, and *SIAH2*. Other miRNAs of interest included members of the miR-30 family, regulating thee mRNAs: *DOC2A*, *SIAH2*, and *TPRG1*. Enrichment analysis and literature search using the STRING database [103] highlighted the fact that this set of genes captured both differences between luminal subtypes and TNBC as well as between high and low tumor grade and tumor invasiveness. Genes *C6orf211*, *ESR1*, *GATA3*, *NPNT*, *RERG*, *SLC39A6*, and *TPRG1* have all been linked with estrogen-positive breast cancers and have been proposed to be part of a prognostic luminal signature [104]. Some genes in this module were connected by processes proposed to be involved in tumor progression, such as *DOC2A*, *CGN* (*Cingulin*), *PARD6B*, *NEDD4L*, and *SYT9* which belonged to the KEGG pathway, tight junction (TJ) (hsa04530), and GEO term, cell junctions (GO:0030054). These genes were downregulated in high TILs|grade tumors (cluster 1). Another gene of interest was *BTRC*, encoding β-transducin repeat-containing E3 ubiquitin protein ligase (β-TrCP).

Noteworthy was that a handful of genes from this network had been annotated as TSGs either specifically in relation to breast cancer (GATA3, RERG, and SIAH2) or in other types of cancer (DOC2A and RGS22). In accordance, genes from this network were all downregulated in high-grade, high-TIL, TNBC samples.

To identify the best differentially co-abundant miRNA-mRNA pairs, we also applied weighted co-expression network analysis to the TIF miRNA dataset. The analysis returned three co-expression modules (blue, turquoise, and red), each encompassing around 1/3 of the miRNAs—Additional file 12: Fig. S8.

The blue and red modules contained miRNAs upregulated in TIFs from high-grade, high-TIL, TNBC tumors (e.g., up in cluster 1 vs cluster 2). In accordance with this, miRNAs predicted to be downregulated in fluids from PgR^+^ vs PgR^−^ tumor were almost exclusively found within this module. The main difference between these two modules was that the red module seemed to capture the miRNAs, which were only found to be DA in *K*-means clusters, not in direct comparisons of TIL status or tumor grade. The slightly smaller turquoise module contained miRNAs downregulated in high-grade, high-immune score tumors—Additional file 12: Fig. S8. miRNAs, which were co-abundant and found to interact with co-expressed genes, may be seen in Fig. 5.

DA miRNAs with predicted mRNA targets from interaction networks and WCGNA, supported by cancer miRNA databases, may be found in Additional file 13: Table S5. WGCNA resulted in subsets of co-expressed genes with accompanying miRNA regulators.

### Known tumor suppressors and oncogenes were predominant within modules correlated with clinicopathological information

As only a few genes from the best miRNA-mRNA interaction pairs were annotated as oncogenes or TSGs, we explored which known (breast) cancer-related genes were co-expressed alongside these within modules. The intersection of co-expression modules with COSMIC census genes and CancerMine genes (with lower cutoff, see the “Materials and methods” section) revealed that while oncogenes and TSGs were equally prevalent within models, e.g., modules were not enriched in one vs another type of gene, there was a difference in the distribution of these genes across modules.

**Fig. 6:**
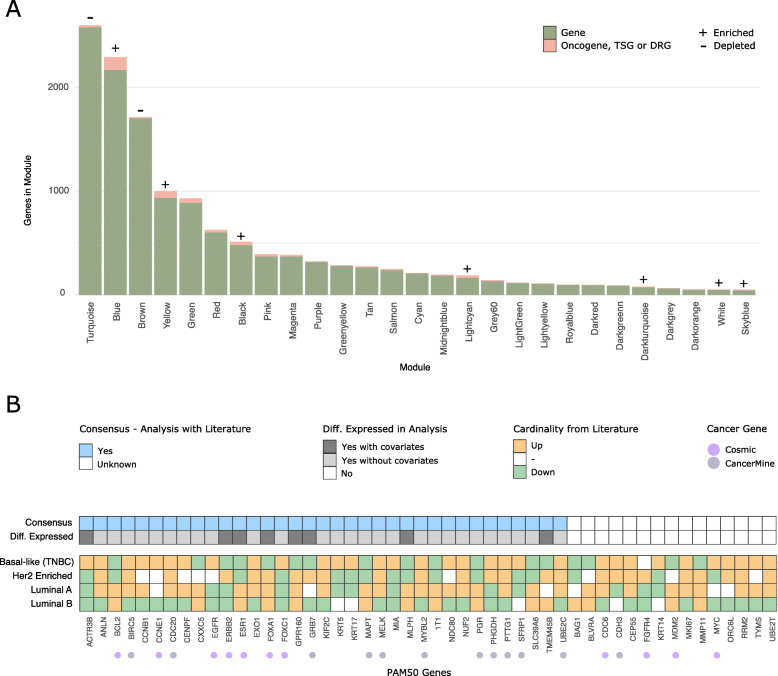
Enrichment of oncogenes, TSGs, and DRGs in modules and PAM50 gene cardinality. **a** Barplot showing the fraction (pink color) of oncogenes, tumor suppressor genes, or dual role genes encompassed by co-expression modules. Modules without any annotated cancer genes are not included, neither is the gray module of genes (genes not found to be co-expressed). Plus signs indicate that a module was significantly enriched for oncogenes, TSGs, or DRGs, while minus signs indicate that the module was significantly depleted of these. **b** Tile plot showing the expression cardinality of PAM50 genes from the analysis compared with the literature. Orange (up) and green (down) colors denote the directionality of genes from the literature for each subtype. Colors dark gray, light gray, and white indicate if a gene was found to be differentially expressed in the current analysis. Light blue and white show the consensus of expression cardinality for a given PAM50 gene between the literature and current analysis. Dots below the tiles highlight which of the PAM50 genes are annotated in the COSMIC Cancer Gene Census set and in the CancerMine database (filtered, see the “Materials and methods” section)

We performed enrichment analysis to examine the depletion or enrichment of annotated genes for all modules. The analysis revealed that seven modules (black, blue, dark turquoise, sky blue, light cyan, white, and yellow) were enriched for genes annotated as oncogenes, TSGs, or DRGs, while two modules (turquoise and brown) were depleted of these types of genes, Fig. 6a. Interestingly, the turquoise and brown modules were also not correlated with any clinicopathological information (Additional file 9: Fig. S5), and in fact, the intramodular connectivity analysis had revealed that these two modules were enriched in highly interconnected LncRNAs (Section 3.6). The blue module, which was highly correlated with breast cancer grade and metastasis (Additional file 9: Fig. S5), encompassed most key breast cancer oncogenes and TSGs, including APOBEC3B, BARD1, BRCA1, BRCA2, BRIP1, CHEK2, DNMT1, EZH2, MAP 3 K13, NF2, POLQ, PPM1D, and TRIM24. The yellow and light cyan modules also contained some well-annotated BC-related genes, such as CASP8, FBLN2, FOXO1, PPARG, and SOCS1. We intersected lists of cancer-related genes from modules with information about whether genes were found to be differentially expressed in any comparison and whether they were included in a miRNA-mRNA network. As was already known from the analysis performed in the “Co-expression modules were correlated with subtype, immune infiltration, and grade” section, mainly the yellow module, out of those enriched for oncogenes and TSGs, encompassed DE genes. The green, red, and grey60 modules, which were correlated with patient information and contained DE genes, were also enriched in cancer-related genes, although this enrichment was not significant after correction for multiple testing (odds ratios, 1.41, 1.22, and 1.91). Among highly annotated BC oncogenes and TSGs in these modules were CCND1, ESR1, GATA3, SLC9A3R1, SMAD4, STAT3, and KLF5.

**Fig. 7:**
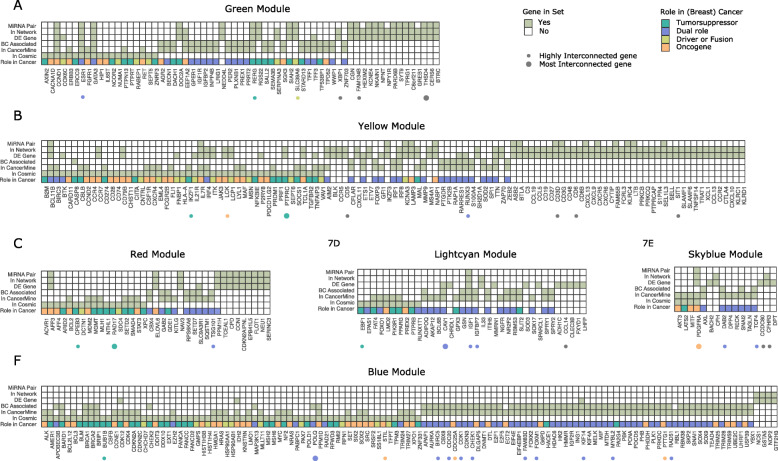
Oncogenes and tumor suppressors in co-expression modules. Tile plots depicting how many genes within five selected modules had been annotated as either an oncogene (orange), a tumor suppressor (green), a dual role/mixed role (purple), or a driver/fusion gene (yellow). The plots contain all genes from modules which were annotated in COSMIC [75] or which met the cutoff for a CancerMine hit [76], as well as all differentially expressed genes and all genes from miRNA-mRNA interaction networks. **a** Green module. **b** Yellow module. **c** Red module. **d** Light cyan module. **e** Sky blue module. **f** Blue module. The large number of dual role genes from CancerMine observed in the plots, arise from the fact that these genes are not curated, and as such have a variety of roles annotated within and between cancers. Additional file 14, Table S6 contains the number of cancer-related genes within each of the 28 modules (3 modules had no genes with annotation)

Interestingly, some genes known to be key players in cancer, here among MAP 3K1|MAP 2K4, NOTCH1|NOTCH3, PIK3CA, SYK, FOXP1, and TP53, were not retained in any co-expression module, e.g., they were encompassed by the gray portion in Additional file 9: Fig. S5. Additional file 8: Table S4 contains for each module information about which genes have been annotated as oncogenes, TSGs, DRGs, or driver/fusion genes in (breast) cancer, and whether the gene was differentially expressed and included in a miRNA-mRNA network. Figure 7 shows which oncogenes and tumor suppressors were co-expressed in the green, yellow, red, light cyan, sky blue, and blue modules.

Next, we looked at which genes from each module were most interconnected, and whether these had been annotated as oncogenes or TSGs (Additional file 8: Table S4 and Fig. 7). Only two modules, the light cyan and sky blue, had an annotated gene, as the topmost interconnected (CAV1 and PDGFRA, respectively), both of which were differentially expressed. Some modules did have (breast) cancer oncogenes and TSGs which were among the top 1% most interconnected, including the blue module (POLQ, rank nr. 3, not DE), green module (ESR1, rank nr. 4), and yellow module (PTPRC, rank nr. 2). Collectively, combining information from COSMIC and CancerMine, with results of DE analysis, network analysis, and WGCNA, confirmed that genes from the green module were specifically related to breast cancer subtype (ER, PGR, and Her2 status), which genes in the yellow module represented tumor immune infiltration (hub gene PTPRC is a tumor suppressor in T cell acute lymphoblastic leukemia), while genes in the blue module were related to progression and metastasis.

Due to the lack of gene expression data from paired normal samples, we were unable to directly compare the expression directionality of known oncogenes and TSGs with expression profiles observed in our analysis. Instead, we looked at the cardinality of the PAM50 genes (some annotated as oncogenes, TSGs, or DRGs), between subtypes in our analysis, with their known cardinality from literature. Only eight PAM50 genes were significantly DE between subtypes in our analysis. However, if we performed DEA without correction for covariates, such as the level of immune infiltration, this number rose to 33 PAM50 genes, indicating that the lack of PAM50 genes in the corrected DE sets, was mainly a result of low power, perhaps in combination with differences between the TNBC subtypes and basal-like cancers. Figure 6b shows that the expression directionality of PAM50 genes was in consensus with that of the literature and their potential role in (breast) cancer [105–107]. Eleven PAM50 genes had been annotated in the COSMIC cancer gene census, out of which seven (BCL2, CCNE1, EGFR, ERBB2, ESR1, FOXA1, and FOXC1) were found to be DE in our analysis, all with a consensus of cardinality between subtypes—Fig. 6b.

### Computational support for miRNA-gene pairs

Finally, we looked into whether miRNA-gene pairs were supported, either by miRNA gene target prediction tools and databases, other than TargetScan [58], or by experimental data. We only considered interactions included in Fig. 5, e.g., the best pairs from combined analyses. Experimentally, validated pairs were obtained from the miRTarBase [67]; however, unfortunately, the miRTarBase database only supported interactions of very well-studied genes and miRNAs such as members of the OncomiR-1 cluster, miR-let-7 family, and genes CCND1, ESR1, and PARD6B. We, therefore, used alternative databases and prediction tools to support TargetScan interactions, requiring that at least three additional methods agreed on a miRNA-mRNA pair. Prediction tools included DIANA-microT (Thermodynamics) [68], miRBridge (complementary, conservation, thermodynamics) [69], PicTar (thermodynamics) [70], PITA (thermodynamics, conservation) [71], rna22 (complementary, conservation) [72], and mirDB (support vector machine) [73]. Results may be found in Table 4.

**Table 4:** Best miRNA and mRNA target candidates. Differentially co-expressed tumor mRNAs from modules and differentially abundant interstitial fluid miRNAs predicted to interact by at least four of the following tools/databases: TargetScan [58], DIANA-microT [68], miRBridge [69], PicTar [70], PITA [71], rna22 [72], and mirDB [73]. miRNAs and mRNAs in this table are shown to interact in Fig. 7

	miRNA name	Direction miRNA	Gene symbol	Direction gene
Luminal vs TNBC	let-7a-5p let-7f-5p let-7 g-5p miR-9-5p miR-15b-5p miR-17-5p miR-18a-5p miR-19a-3p miR-30d-5p miR-23a-3p miR-34c-5p miR-98-5p miR-107 miR-129-5p miR-141-3p miR-148a-3p miR-221-3p miR-222-3p miR-301a-3p miR-454-3p	Down	CCND1 CERS6 ESR1 KIF3B THSD4	Up
Luminal B vs TNBC	miR-10b-5p miR-19a-3p miR-23a-3p miR-30d-5p miR-31-5p miR-135a-5p miR-135b-5p miR-301a-3p miR-454-3p	Down	CDKN2AIPNL ELOVL6 EPB41L5 FLOT1 SERINC3	Up
High-grade tumors	miR-10a-5p miR-10b-5p miR-107 miR-301a-3p miR-301b-3p miR-454-3p	Up	BTRC CHST1*	Down
High levels of TILs	miR-103a-3p miR-136-5p miR-206 miR-146a-5p miR-299-5p miR-494-3p	Down	BTLA BCL11B CD3G LTB LAMP3 KLRC4	Up
TNBC and high-grade and high levels of TILs	let-7a-5p let-7c-5p let-7d-5p let-7e-5p let-7f-5p let-7 g-5p miR-9-5p miR-10a-5p miR-10b-5p miR-17-5p miR-18a-5p miR-19a-3p miR-19b-3p miR-20a-5p miR-20b-5p miR-23a-3p miR-23b-3p miR-27b-3p miR-29a-3p miR-29b-3p miR-30a-5p miR-30b-5p miR-30c-5p miR-30d-5p miR-31-5p miR-32-5p miR-33b-5p miR-34c-5p miR-98-5p miR-125a-5p miR-106a-5p miR-106b-5p miR-130a-3p miR-130b-3p miR-135a-5p miR-138-5p miR-141-3p miR-182-5p miR-186-5p miR-192-5p miR-196b-5p miR-200a-3p miR-223-3p miR-301a-3p miR-301b-3p miR-330-3p miR-340-5p miR-374a-5p miR-454-3p	Up	CGN DOC2A FAM134B GATA3 NKAIN1 NPNT PARD6B RERG RGS22 SLC39A6 SYT9 TPRG1	Down

Summary table showing the most interesting miRNAs and gene targets for BC classification, based on all analysis. All miRNAs and genes in this table are differentially abudant and co-abundant in sets, and their interactions are predicted by at least four different tools for miRNA—gene target predictions tools and/or experimental validation. A single gene, CHST1, denoted by an asterisk was not co-expressed but still included due to interest from the literature search

## Discussion

In this study, we integrated interstitial fluid miRNA abundances with expression levels of mRNA from paired tumor tissues. Our analysis allowed us to explore whether miRNAs secreted into the interstitium could be associated with differentially expressed gene targets and whether these targets were co-expressed and/or co-regulated. We partitioned the data based on sample molecular and clinical information to obtain sets of differentially expressed IF miRNA and their intracellular gene targets, hereby elucidating potential pathways and mechanisms underlying breast cancer.

As expected, we observed a good separation of BC subtypes based on intracellular mRNA expression; however, this was not the case for the interstitial fluid miRNAs. Although we did see some clustering of TIF from TNBC samples, this could just as well be related to the common higher immune status and higher tumor grades of these samples [50]. Other studies on circulating miRNA expression in BC patients have found similar trends, with a poor distinction of different subtypes, except for TNBC (or basal-like) tumors [108–110]. The majority of differentially abundant interstitial fluid miRNAs identified in our study were DE in the contrast of normal vs cancer or associated with immune infiltration and tumor grade. These results are in accordance with previously published literature on circulating miRNA BC from the serum/plasma, as these most often highlight miRNA profiles related to cancer progression, invasiveness, metastasis, and relapse [111–114]. In more quantitative measures, this is supported by a PubMed search on titles and abstracts (the “Materials and methods” section). A search on terms related to circulating miRNAs + cancer progression yielded 757 results (18 titles), whereas the search with terms circulating miRNAs + subtype only returned 106 results (four titles). A further look into the four articles with subtype terms in the title revealed none of them to find differences between PAM50 or immunohistochemistry subtypes.

When comparing the results of our miRNA analysis to those obtained in the original study by Halvorsen et al. [49], results were highly variable. We hypothesize that discrepancies mainly arise from the following:

- *Choice of statistical framework*. limma [53], which was employed in our analysis, is likely to return a larger number of significant DA miRNAs, compared to the Kruskal-Wallis test used in the original analysis [49]. This is due to limma’s underlying Bayesian properties, which help overcome issues relating to small sample sizes and miRNA-specific variances.
- *Correction for batch effects and confounders*. Clustering of datasets revealed significant confounding of covariates; as such, we incorporated information on confounders into the design matrix for generalized linear modeling with limma.
- *Integrative analysis*. As we performed an integrated analysis, including co-abundance analysis and collective analysis of both TIF miRNA data and paired intra-tumor mRNA data, we naturally curated our results based on miRNA abundances, as well as the relationship between miRNAs and predicted differentially expressed mRNA targets. As such, we obtained a very different set of miRNA top candidates for further analysis and validation.

In addition to the aforementioned, other differences may have contributed to varying results, (i) how comparisons were defined, (ii) cutoff for retaining a miRNA in the dataset, (iii) cutoff for significance (log fold change was added as a criterion in our analysis), and (iv) missing value imputation.

We believe that the solid bioinformatic framework and data integration implemented in our study have resulted in new and valuable biological insights while highlighting the major impact of data correction and choice of statistical set-up has for down-stream results.

Overall, our analysis revealed a set of miRNAs which were upregulated in tumor interstitial fluids from mainly TNBC patients with high-grade and high immune infiltration score tumors. Subsets of these miRNAs were predicted to have target genes, which were also differentially expressed in tumors from the same cohort of patients. Table 4 shows the overall best candidate interstitial fluid miRNAs and predicted gene targets, based on all analyses and database support.

In the following sections, we will discuss some of these most interesting miRNAs and genes, in greater detail.

### Breast cancer subtypes

#### Common to subtypes

The most interconnected DE miRNAs from the comparisons of BC subtypes were miR-9, miR-15b, miR-17, miR-19a, and miR-30d. We found these to be depleted in tumor interstitial fluids from patients with luminal and Her2-enriched breast cancers, compared to samples from TNBC patients. Interestingly, all of these miRNAs have been shown to be highly abundant in the basal-like BC subtype, which is largely similar to TNBC [115, 116]. Patients with basal-like/TNBC tumors are known to have the poorest prognosis, and this subtype is associated with high-grade and rate of metastasis [8]. In accordance with this, miR-17 and miR-19a belong to the miR-17-92 cluster, also denoted OncomiR-1 (13q31.3) [117]. We found these two miRNAs, along with other members of OncomiR-1 (miR-19b, miR-18a, and miR-20a), to be differentially co-expressed. The miR-17-92 cluster of miRNAs has been shown to target the well-studied tumor-suppressor PTEN (*phosphatase and tensin homolog*), as well as key players involved in TGF-*β* (*transforming growth factor beta*) signaling [118].

Multiple studies on miR-17 have found an association between the over-expression of this family of miRNAs with poor patient prognosis (poor disease-free survival and overall survival) [119, 120] and, in connection with this, cancer cell migration and invasion in breast cancer [121, 122].

Over-expression of miR-30d and miR-9 has been associated with an aggressive phenotype, shorter time to recurrence, and a poor prognosis in patients with breast cancer [123, 124]. More specifically, miR-30d is proposed to be an inhibitory regulator of autophagy [125], and the miR-30 family of miRNAs is thought to promote non-attachment growth of breast cancer cells [126].

MiR-9, miR-15b, miR-17, miR-19a, and miR-30d were predicted to interact with a set of differentially expressed genes, some of which were common to the three subtype comparisons. Common genes were *AR* (*androgen receptor*), *CERS6* (*ceramide synthase 6*), *FOXA1* (*forkhead box A1*), *GPR160* (*G protein-coupled receptor 160*), *KIAA1244* (*ARFGEF family member 3*), KLK5 (kallikrein-related peptidase 5), SPDEF (*SAM pointed domain-containing ETS transcription factor*), and *XBP1* (*X-box binding protein 1*), all of which were upregulated in luminal types and Her2-enriched TIF samples vs TNBC. Three of these genes belonged to the PAM50 set: *AR*, *FOXA1*, and *GPR160* [7], while the remaining genes had all been individually associated with breast cancer subtypes [92–96].

#### Luminal subtypes

While some genes were common to the three contrasts, others were subtype-specific, such as *ESR1* (*estrogen receptor 1*), *KIF3B* (*kinesin family member 3B*), KRT4 (*keratin 4*), and *NFIB* (*nuclear factor I B*), which were associated with luminal types only. *KIF3B* was upregulated in the luminal samples, and in accordance with this, *KIF3B* has been shown to be over-expressed in ER-positive tumors, with estrogen directly inducing the expression of KIF3B [127]. KRT4 and NFIB were downregulated in luminal subtypes compared to TNBC. *KRT4* and *NFIB* have both been shown to be over-expressed in basal/TNBC tumors [128, 129], supporting our findings. The expression levels of keratins change during metastatic progression of breast cancer, and over-expression of some keratins have been associated with poor patient survival [130]. Of particular interest was NFIB, which has directly been proposed as a potential gene target for ER-negative breast tumors. NFIB was found to be over-expressed in TNBC compared to ER-positive tumors, and over-expression of this gene was associated with a high nuclear grade [129]. *ESR1* and *CERS6* (see the section above) were co-expressed in the green module, along with *C6orf211* (*ARMT1*, *acidic residue methyltransferase 1*), *CCND1* (*cyclin D1*), and *THSD4* (*thrombospondin type 1 domain containing 4*). This set of genes has been suggested as markers for a prognostic luminal signature [104] and has more recently been highlighted as the key players in a novel, *FOXA1/ESR1-interacting pathway* [131], highlighting their association with estrogen receptor status.

#### Luminal A subtype

The gene *CCND1* was upregulated and highly interconnected in the luminal A vs TNBC comparison. *CCND1* is a well-studied breast cancer driver gene [132], the amplification of which is more prevalent in luminal subtypes compared to Her2 and basal-like [133]. Amplification of this gene has been found to be more prevalent in luminal B tumors compared to luminal A [134]. However, as *CCND1* amplification is also associated with a more aggressive phenotype within both luminal subtypes, as well as in familial and sporadic tumors [134], this might explain the slight discrepancy we observe here, e.g., different compositions and sizes of luminal sets, resulting in this gene not reaching significance in the luminal B vs TNBC comparison. A comparison of miRNA-gene pairs with MiRTarBase resulted in support for the *CCND1* gene and its predicted miRNA regulators.

#### Luminal B subtype

For the contrast of luminal B vs TNBC, the *ELOVL6* (*ELOVL fatty acid elongase 6*) gene was found to be upregulated and interact with both a larger number of genes and miRNAs. A high level of *ELOVL6* (oncogene in prostate cancer) has been proposed to be a marker of poor prognosis in BC [135], which is of great interest, as patients with luminal B type tumors generally have poorer outcomes than those with luminal A types [8]. Dysregulated expression of genes involved in mammary gland fatty acid and phospholipid metabolism, such as the *ELOVL6* gene, have been proposed to characterize cell proliferation and differentiation state, and many of these have been linked to BC patient survival [136]. STRING network analysis with the set of eight co-expressed from the red module (including *ELOVL6*), returned the gene ontology term sphingolipid metabolism. Genes assigned to this term were *ELOVL6*, *NEU1* (*neuraminidase 1*), and *SERINC3* (*serine incorporator 3*). A literature search revealed that another gene from this module, *FLOT1* (*flotillin*), had recently been linked to the sphingolipid pathway, proposed to be a regulator of cellular sphingolipid distribution and signaling [137]. All genes from the red module, except *CCN1*, were specifically upregulated in luminal B vs TNBC, but not in luminal A type, indicating that over-expression of sphingolipid-related genes might be specific to luminal B tumors. Interestingly, *ELOVL6*, *NEU1*, and *SERINC3* were the predicted targets of miR-23a, which was also highly interconnected and downregulated in the luminal B vs TNBC comparison. A literature search for miR-23a revealed this miRNA to be a well-known oncogenic miRNA, and a recent study by Ma et al. [138] found that over-expression of miR-23a induced EMT, migration, invasion, and metastasis of breast cancer both in vitro and in vivo [138]. miR-23b has been proposed to be a circulating biomarker for BC diagnosis, subtyping, and disease recurrence [139], many times over, highlighted by a novel review on this miRNA [140].

#### Estrogen-positive tumors

The DE expression network generated for ER^+^ vs ER^−^ tumors and interstitial fluids showed *ESR1*, *GATA3* (*GATA binding protein 3*), and *GREB1* (*growth-regulating estrogen receptor binding 1*) to all be upregulated, while *ERBB2* (*Erb-B2 receptor tyrosine kinase 2*) was downregulated. Both *GATA3* and *GREB1* have been linked to estrogen receptor-positive breast tumors and have been proposed as markers for patient response to hormone treatment [141–143].

The most interesting miRNA from this network was miR-32-5p, which was over-expressed in ER^+^ tumors vs ER^−^ tumors, and the most interconnected miRNA in the network. Not much is known about this miRNA in connection with breast cancer; interestingly, however, miR-32-5p interacts with genes *NFIB*, *SOX11* (*SRY-box 11*), and *DSC2* (*desmocollin 2*) (downregulated in ER^+^ vs ER^−^), all three of which are known to be over-expressed in basal-like/TNBC/ER^−^ tumors and associated with poor survival [129, 144, 145].

#### Her2-enriched subtype

Specific to the contrast Her2-enriched vs TNBC, were genes ERBB2, GRB7 (*growth factor receptor bound protein 7*) and LASP1 (*LIM and SH3 protein 1*), all of which were upregulated. These genes are well-supported central players in Her2-enriched cancers and belong to the Her2 amplicon (chromosome region 17q-12-21) [98]. ERBB2 and GRB7 are both Pam50 genes [7]. Another gene specific to the Her2 set was CPD, which overall had the most interactions in the miRNA-mRNA network. CPD (*carboxypeptidase D*) is another gene known to be amplified in patients with Her2-enriched tumors on chromosome 17, right upstream of ERBB2 (chromosome region 17q-11-2) [146].

### Tumor-infiltrating lymphocyte scores and tumor grade

Analysis of the miRNA-mRNA pairs differentially expressed in high TILs (2, 3) vs low TILs (0,1) revealed the *NEDD4L* (*NEDD4 like E3 ubiquitin protein ligase*) gene, to be paired with the highest number of miRNAs. *NEDD4L*, which was downregulated in samples with high tumor-infiltrating lymphocytes scores, has been shown to be a negative regulator of Wnt-signaling [147]—a pathway often perturbed in cancer [148]. Wnt signaling is central in the regulation of immunity and has been reported to facilitate immune evasion via dendritic cells and T regulatory cells [149]. In a study by Ding et al. [147] on *NEDD4L* inhibitory effects on the Wnt signaling, it was noted that *NEDD4L* is often found to be downregulated in cancers, while its Wnt-target Dvl (*disheveled*), which is modified by *NEDD4L* for proteasomal degradation, is often upregulated in the same cancers [147]. This could indicate that the accumulation of Dvl contributes to an oncogenic type of Wnt signaling. Furthermore, the downregulation of *NEDD4L* has been implicated in the initiation of breast tumor development, and this gene has been proposed as a prognostic lung cancer marker linked to histological grade, tumor stage, and lymph node metastasis [86, 150]. These findings support the results of our analysis, as samples with high immune scores were also those with a high histological grade (grade 3 tumors). Additionally, our analysis revealed that NEDD4L interacts with PARD6B (*Par-6 family cell polarity regulator beta*) and CGN (cingulin). These two genes were co-expressed in the green module and were downregulated in cluster 1 (high TILs and high-grade, mainly TNBC) vs cluster 2 (low TILs and lower grade, mainly luminal). *PARD6B*, *CGN* (*cingulin*), and *NEDD4L* belong to the KEGG pathway, tight junction (TJ) (hsa04530). Aberrant levels of tight junction proteins result in incorrect formation and maintenance of cellular polarity, contact inhibition, and proliferation, contributing to epithelial-mesenchymal-transition (EMT) [151]. *PARD6B* expression is critical for TJ assembly, and decreased expression of this gene has been proposed to result in epithelial cell changes and tumor metastatic behavior [152]. *PARD6B* has been shown to be amplified in breast cancer [153]; however, in a comparison of BC subtypes, the expression of this protein was specifically proposed to be upregulated in the luminal type compared to basal-like and Her2-enriched tumors [154]. This observation is in accordance with our findings, as it was had a higher expression level in cluster 2 than in cluster 1. Although *PARD6B* is generally considered to be an oncogene, it has also been linked to suppression of cell proliferation in breast cancer, indicating that the role of this gene may be complex [155]. *PARD6B*, *CGN* (*cingulin*), *a*nd *NEDD4L* were all predicted targets of the OncomiR-1 (13q31.3) cluster, or one of its paralogues 106a/363 (Xq26.2) and 106b/25 (7q22/1), miRNA included miR-17, miR-19a/b, miR-20a/a, and miR-106a/b. In accordance with this, *NEDD4L* has experimentally been shown to be the gene target of the miR-106-25 cluster miRNAs [86].

#### Tumor grade

Network analysis of miRNA-mRNA DE pairs high-grade tumors (grade 3) vs medium/low-grade tumors (grades 1, 2) revealed two genes of interest. One of these genes, *BTRC* (*beta-transducin repeat containing E3 ubiquitin protein ligase*) predicted to interact with miR-10a/b and miR-107. Interestingly, we found these three miRNAs to be co-abundant (module 1, Fig. 5). One study on miRNA-10b found that this miRNA was secreted via exosomes and that the uptake of these exosomes by recipient cells resulted in a decrease of target gene levels and induced invasiveness in otherwise non-malignant cells [24]. Whereas miRNA-10b is generally considered to promote tumor progression and metastasis [156], the role of miR-107 in breast cancer seems less straightforward. Some studies suggest that miR-107 has a tumor-suppressive role [157], while others have found that over-expression of this miRNA promotes tumor progression, is associated with lymph node metastasis and poor patient prognosis [113, 158]. Just as for miR-107, the role of β-TrCP (encode by *BTRC*) in cancer development and progression is convoluted. *BTRC* has been proposed to be a DRG, having oncogenic properties in one context and anti-tumor functions in another [159]. More recent literature on β-TrCP, however, suggests that this protein indeed suppressed tumor progression, as one study showed that β-TrCP regulates the degradation of CDK1, high levels of which promote certain aspects of tumor malignancy [160]. Another study on β-TrCP in glioma found that a low level of this protein was associated with a poor prognosis [161]. Our results agree with these studies; we see a downregulation of BTRC in high-grade tumor tissues and an upregulation of miR-10a, miR-10b, and miR-107 in matched interstitial fluids of these tumors. Importantly, the interaction between miR-10a and the BTRC transcript has been experimentally validated (luciferase reporter experiment) [162]. Although we could not find any experimental validation for the BTRC-miR-107 interaction, a study by Yang et al. [163] found that a combination of miR-107-BTRC-UBR3-miR-16 expression could distinguish between different BC subtypes, specifically between basal-like tumors and luminal types [163].

Another gene of interest in relation to tumor grade was *CHST1* (*carbohydrate sulfotransferase 1*), which was paired with miRNAs miR-301a/b and miR-454. Analysis revealed miR-301a/b and miRNA-454 to be co-abundant in the same module as miR-10a/b and miR-107 (module 1, *5*), supporting the notion that these miRNAs might be associated with tumor grade and progression. The literature on *CHST1* and cancer is very limited; however, studies on other members of the carbohydrate sulfotransferase (CS) family show that while some CS members may be oncogenic, others could have tumor suppressor functions. Overexpression of *CHST3* and *CHST11* have been linked to BC aggressiveness, relapse, and development of metastasis [164]; in contrast, downregulation of *CHST10* and *CHST14* has been linked to invasive melanoma and to late stages of colon cancer progression, respectively [165, 166]. In the current study, we found *CHST1* to be downregulated in grade 3 vs grade 1|2 tumors. The miRNAs predicted to interact with *CHST1* are more well-studied then their target. MiR-301 is thought to be a breast cancer oncomiR, which promotes tumor invasion and nodal or distant relapses via direct interaction with *FOXF2*, *PTEN*, *BBC3iso-2*, and *COL2A1* [167]. This microRNA has also been shown to help regulate cancer-related immunity in solid tumors [64]. In accordance with this, we found miR-301a and miR-301b to be upregulated both in the contrast of IF from high grade to medium/low grade and between high TILs and low TILs. High expression of miR-454 has been associated with a poor overall and disease-free survival in patients with TNBC [168]. These findings were supported by a meta-study by Lu et al. [169], although this review also highlighted the fact that miR-454 might have a dual role, exerting oncogenic effects in some cancer types, such as breast cancer, and tumor-suppressor functions in other types of cancer [169].

#### Tumor-infiltrating lymphocytes

Analyses revealed a set of TIF miRNAs and co-expression gene targets, which were associated with tumor-infiltrating lymphocyte scores. This network included miRNAs; miR-103a, miR-136, miR-146a, miR-299, miR-301, miR-346, miR-369, and miR-494 predicted to interact with gene transcripts: *ASB2*, *BCL11B*, *BTLA*, *CD3D*, *CD3G*, *CXCL13*, *CXCR5*, *FAM65B*, *IKZF3*, *IL7R*, *KCNA3*, *KLRC4*, *LAMP3*, and *LTB*. This set of genes, which were all upregulated in high-TIL vs low-TIL samples, has all been linked to immune system processes [170–173]. Genes such as *CD3D* and *CD3G* encode T cell surface glycoproteins and are well-known players in anti-tumor immunity [174]. High levels of these two antigens have been linked to an overall better prognosis of patients with breast cancer [175, 176]. The same is true for *BCL11B*, *IKZF3*, and *KLRC4*, which have very recently been linked to a prognostic immunogenic signature of triple-negative breast cancers [173, 177]. Liu et al. [177] found that almost all populations of immune cells, immune system pathways, and their genes were enriched in TNBC compared to both normal samples and other breast cancer subtypes. This is in accordance with our findings; we see TNBC having not only overall higher grade but also infiltrating lymphocyte scores.

Of particular interest was the co-expression of genes: *BTLA*, *CXCR5*, *CXCL13*, *IL7R*, *LAMP3*, and *LTB*. The protein products encoded by genes have been linked to the presence or absence of high endothelial venules and tertiary lymphoid structures in multiple cancer types [178–181]. TLS, which are lymphoid formations, have been found within tumors where they are thought to participate in anti-tumor responses. A high number of TLS is generally associated with an overall better patient’s survival in a range of different types of cancer [182], and their presence correlates with the level of both TILs and HEV [179, 180]. These observations are supported by the fact that high endothelial venules, which are specialized vessels normally found in the lymph nodes, are proposed to act as gateways for the infiltration of lymphocytes within tumors [183]. The abundance of lymphoid chemokines such as CXCR5 and *CXCL13* has been linked to both the presence of TLS and HEV in breast cancer stroma [181]. Tertiary lymphoid structures are modulated by a network of cytokines, and the central players in this network are lymphotoxin LT-β-related cytokines [179]. One study [178] found that lymphotoxin LT-β was overexpressed in breast tumors and that overexpression of LT-β was correlated with a high density of HEVs and dendritic cells. Dendritic cells are thought responsible for the production of LT-β in tumor tissues in general and in tertiary lymphoid structures. These findings might indicate that a high level of LT-β should be predictive of a better patient outcome. However, another study on the LT-β network in mice has shown that high levels of lymphotoxin LT-β promote a tumor-permissive microenvironment resulting in tumor progression [184]. The results of our analysis support those from the aforementioned studies, with this set of genes found to be upregulated in samples with high levels of lymphocyte infiltration. For a more in-depth description of the relationship between TLS, TILs, and HEV, as well as the roles of *BTLA*, *IL7R*, and *LAMP3* in relation to these, we refer to the original publications [178–180, 184].

The set of co-expressed immune genes discussed above was mainly predicted to be the targets of miR-146a and miR-494. We found these miRNAs, along with miR-206, miR-369, and miR-376a, to be co-abundant (module 2, Fig. 5). Both miR-146a and miR-494 have been linked to immune system response in connection with tumor development [185–188]. miR-146a is a central player within the innate immune system, where it functions as a fine-tuning mechanism, modulating the scale of immunity vs tolerance [189]. Generally, this miRNA is considered a negative regulator of immune response. This is supported by mouse knock-down experiments, in which loss of miR-146a was shown to result in autoimmunity and development of myeloid malignancies [189, 190]. Re-establishing miR-146a expression within breast cancer has been shown to decrease the levels of immunostimulatory genes and to antagonize NF-kB signaling, reducing cancer cell migration and metastatic mechanisms [185, 187]. The role of miR-494 in cancer immunity is not straightforward. One study found that this miRNA might help prevent anti-tumor immunity through the accumulation of myeloid-derived cells in the microenvironment, promoting tumor growth [186], while another study showed that miR-494 suppresses the progression of breast cancer, through downregulation of CXCR4-mediated oncogenic communication [188]. Although miR-146a and miR-494 had the most gene targets within the co-expressed immune gene cluster, other miRNAs were also of interest here among miR-103a, miR-301, and miR-369 all of which have been linked to tumor immunity [64, 191]. A search thought the CMEP database revealed all of these to be DE in the blood of BC patients. miR-103a, miR-301a, miR-494, and miR-369 were all downregulated in TNBC compared to other subtypes. This is in full accordance with our results, as high immune score tumors were mainly TNBCs.

## Conclusion

We identified genes that were differentially co-expressed between tumors with high and low infiltrating lymphocyte scores—most of these had already been associated with cancer immunity through other studies [170–173]. Of particular interest were *CXCL13*, *BTLA*, *IL7R*, *LAMP3*, and *LTB* as these genes have been linked to the presence of tertiary lymphoid structures (TLS) and high endothelial venules (HEV) within tumors. TIF miR-146a and miR-494, the most interconnected and co-abundant miRNAs in this cluster, were both previously annotated as negative regulators of immune-stimulatory genes and were DE in the plasma from patients with BC [158, 192]. As tumor immune cell infiltration is highly related to patient prognosis [2], we propose genes and miRNA from this module to be candidate markers of tumor immune status, prognosis, and potentially patient response to immunotherapy.

Another co-expression module encompassed genes, which were DE between luminal B tumors and TNBC. A subset of these was related to sphingolipid metabolism and predicted to be co-regulated by miR-23a. miR-23a has been found to be differentially abundant in the serum of healthy individuals and breast cancer, as well as between BC patients with different subtypes [140]. As such, this miRNA is a candidate marker for BC subtype and potentially a new therapeutic target. TIF miRNAs DE between subtypes were all identified in contrasts of TNBC vs another subtype. Many miRNAs identified in these contrasts were generally related to BC progression and metastasis, such as members of the OncomiR clusters and miRNA families miR-30 and let-7. This observation is supported by other studies on secreted miRNAs, and we therefore propose that levels of secreted miRNAs do not reflect gene-based subtyping, but rather tumor aggressiveness, i.e., TNBC patients often have higher-grade tumors and a poor prognosis.

A small set of genes and TIF miRNAs were more specifically associated with tumor grade, here among miR-10a/b and gene target *BTRC*. The interaction of miR-10-*BTRC* has been experimentally validated [162], and miR-10b was found to be delivered via exosomes to recipient cells, resulting in the downregulation of target genes [24]. *BTRC* is proposed to have tumor-suppressive functions [160, 161], while miR-10b is oncogenic; as such, it should be of interest to study this pair in relation to tumor invasiveness and metastasis.

Collectively, integration of expression data from interstitial fluid miRNAs and paired solid tissue mRNAs resulted in sets of miRNA-mRNA pairs, associated with underlying molecular mechanisms and clinical features of breast cancer.

Whether TIF miRNAs highlighted in our study are indeed transferred between cells in the tumor microenvironment, or whether these merely reflect that level of miRNAs within the tumor donor cells themselves, is unknown. However, as the uptake of miRNAs from the extracellular space is a well-known phenomenon, communication and transcriptome regulation via interstitial fluid miRNAs are an attractive therapeutic angle for cancer treatment.

## Supplementary information

**Additional file 1: Figure S1.** Multidimensional Scaling Plot. Plot depicts the relationship (squared euclidian distances) between breast cancer samples based on the abundance of interstitial fluid miRNA or expression of intra-tumor mRNA. S1A = clustering based on miRNA abundances in IFs before correction for patient-specific effects. Colors: grey = normal interstitial fluids, red = tumor interstitial fluids. S1B = clustering based on miRNA abundances in IFs after correction for patient specific effects (heterogeneity). Colors: grey = normal interstitial fluids, red = tumor interstitial fluids. S1C = clustering based on miRNA abundances in TIFs. Colors denote BC subtypes: dark green= luminal A, light green = luminal B, pink = luminal B Her2-enriched, orange = Her2-enriched and deep red = TNBC. S1D = clustering based on intra-tumor mRNA expression. Colors denote BC subtypes: dark green= luminal A, light green = luminal B, pink = luminal B Her2-enriched, orange = Her2-enriched and deep red = TNBC.

**Additional file 2: Table S1.** K-means Clusters. Partitioning of samples into two K-means clusters (C1, C2), based on interstitial fluid miRNA abundances or intra-tumor mRNA expression levels. Dot represents an un-matched sample. The column “consensus” denotes whether the sample was assigned to the same cluster based both on miRNA and mRNA levels.

**Additional file 3: Figure S2.** Comparison of Differentially Abundant miRNAs. Comparison of differentially abundant miRNAs from current analysis with original publication (Halvorsen, et al. 2017). S2A = Comparison of miRNAs DA in TIF vs NIF, and expressed in paired serum, set include (i) miRNAs DA between TIF vs NIF, from Halvorsen, et al. 2017, (ii) miRNAs DA between TIF vs NIF, also in serum, from Halvorsen, et al. 2017, and (iii) miRNAs DA between TIF vs NIF from current analysis. S2B = Comparison of miRNAs DA between BC subtypes. Sets include (i) miRNAs DA between subtypes, from Halvorsen, et al. 2017, (ii) miRNAs DA between subtypes significant after correction for multiple testing, from Halvorsen, et al. 2017, (iii) miRNAs DA between subtypes significant from current analysis and (iv) miRNAs DA between ER^+^ and ER^-^ from current analysis. S2C = Comparison of miRNAs associated with the degree of tumor infiltrating lymphocytes. Sets include (i) miRNAs associated with TILs and tumor percentage, from Halvorsen, et al. 2017, (ii) miRNAs DA between high (+2|+3) vs low TILs (0/1) from current analysis. (iii) miRNAs DA between high (gr 3) vs low/medium tumor grade (gr 1|2) from current analysis, and (iv) miRNAs DA between TIF Cluster 1 vs Cluster 2 from current analysis.

**Additional file 4: Table S2.** This table contains sets of genes (mRNAs) from miRNA-mRNA networks, with accompanying information on logFC, adjusted *p*-values, information from the ***COSMIC database*** about frequency of mutations (predicted to be pathogenic), copy number variations (loss, gain) and information about known role in cancer. Additionally the table(s) also contains information about genes from ***CancerMine text-mining tool***, e.g. if a given gene has been referred to as an oncogene, driver gene or tumour suppressor in literature. Genes are ranked based on mutational burden and CNVs.

**Additional file 5: Figure S3.** miRNA-Gene Interaction Networks. Networks of differentially expressed miRNAs and gene targets predicted by TargetScan. Colors refer to expression directionality, red = up-regulated, black = down-regulated. S3A = TIF Cluster 1 vs Cluster 2, S3B = High TILs (+2|+3) vs low TILs (0|+1), S3C = High-grade (gr 3) vs medium/low-grade (gr 1|2), S3D = Her2 vs TNBC, S3E = luminal A vs TNBC, S3F = luminal B vs TNBC, S3G = ER^+^ vs ER^-^.

**Additional file 6: Table S3.** Set-wise Results of Differentially Abundance/Expression Analysis. List of differentially expressed intracellular mRNAs and interstitial fluid miRNAs from pairs which were either common across sets (Table S3.1) or unique to sets (Table S3.2).

**Additional file 7: Figure S4.** Module Relationships. The hierarchical clustering and heatmap show how similar the modules are (correlation scale on the side). The color assignment is reported as well on the X and Y axes.

**Additional file 8: Table S4.** Intramodular Connectivity. The table contains results from the intramodular connectivity analysis, including gene name, the module a gene belonged to, and interconnectivity scores; kTotal (whole network), kWithin (within module), kOut = kTotal-kWithin, and kDiff = kWithin-kOut. Additionally, the table includes information about whether a gene was annotated as an oncogene | tumor suppressor gene | dual role gene | driver gene | fusion gene in the COSMIC census gene set, or in the filtered results of CancerMine text-mining. The columns “DE Gene” and “Gene from Network” denote if a gene was found to be differentially expressed in any comparison and whether it was included in one of the miRNA-mRNA network, respectively. The genes are ranked based on interconnectivity within modules.

**Additional file 9: Figure S5.** Intra-tumor mRNA Co-expression Modules. Results of weighed Gene Co-expression Network Analysis. Upper part of plot shows the clustering of the genes co-expressed in the 31 modules from Weighed Gene Co-expression Network Analysis (WGCNA). Grey denotes that the gene was not assigned to any module. Modules are named by their color. Lower part of plot shows the correlation between patient clinical variables and modules.

**Additional file 10: Figure S6.** Overlap of Differentially Expressed mRNAs with Co-expressed mRNAs. Venn diagrams, depicting the overlap between differentially expressed mRNAs from contrasts, with modules, which were correlated with the patient clinical feature of interest.

**Additional file 11: Figure S7.** Heatmaps of DE intra-tumor mRNAs levels. Heatmaps showing the separation of tissue samples, based on the best DE mRNA candidates from Weighed Gene Co-expression Network Analysis (WGCNA). S7.A: luminal (A + B), Her2-enriched vs TNBC, S7.B: TIL scores, S7.C: tumor grade (plus clusters minus genes from the TILs comparison) and S7.D: estrogen receptor status. Color scale denotes expression levels, purple = high expression, and yellow = low expression.

**Additional file 12: Figure S8.** Results of miRNA Co-abundance Network Analysis. WGCNA resulted in three miRNAs co-abundance modules, denoted Module 1 (Blue, S7 A), Module 2 (Turquoise S7 B) and Module 3 (Red, S7 C). Shapes indicate which contrast a given miRNA was differentially abundant within. X-axis = name of miRNA, y-axis = log fold change for miRNA in contrast.

**Additional file 13: Table S5.** Interstitial Fluid miRNA and Intra-Tumor mRNA Targets Supported by Databases. Differentially abundant miRNAs with predicted mRNA target(s) from interaction networks and WCGNA, supported by cancer miRNA databases. Databases were: *(I) CMEP (Circulating MicroRNA Expression Profiling)*http://syslab5.nchu.edu.tw/CMEP/*(II) dbDEMC database of (Differentially Expressed MiRNAs in human Cancers)*http://www.picb.ac.cn/dbDEMC/*and (III) miRCancer (microRNA Cancer Association Database)*http://mircancer.ecu.edu/download.jsp*. Database information on miRNA expression is included*, e.g. *directionality in comparison*, *experimental design.*

**Additional file 14: Table S6.** Oncogenes and Tumor Suppressors in Co-expression Modules. Co-expressed genes from modules that were annotated as oncogenes, tumor suppressors, dual role genes, driver genes or fusion genes by COSMIC and/or CancerMine. As CancerMine is a prediction tool, we imposed a cut-off of minimum of 10 annotations/citations for a gene (cancer other and pan-cancer) and a minimum of 5 annotations/citations for genes specifically associated with breast cancer (See Materials and Methods).

**Additional file 15: Table S7.** Experimental Support for miRNA and Gene Target Interaction. Prediction Differentially abundant tumor interstitial fluid miRNAs and their experimentally validated intracellular gene targets with support from MiRTarBase (Chou et al., 2017), release 7.0.

## Data Availability

The scripts used to reproduce our research and relative information are available in our GitHub repository: https://github.com/ELELAB/miRNA-mRNA_TIF
