# Supplementary figures and images for: Secreted breast tumor interstitial fluid microRNAs and their target genes are associated with triple-negative breast cancer, tumor grade, and immune infiltration
Source: Breast Cancer Res. 2020 Jun 30;22:73. doi: 10.1186/s13058-020-01295-6 (PMC7329449; doi:10.1186/s13058-020-01295-6)

Fig. S1 A

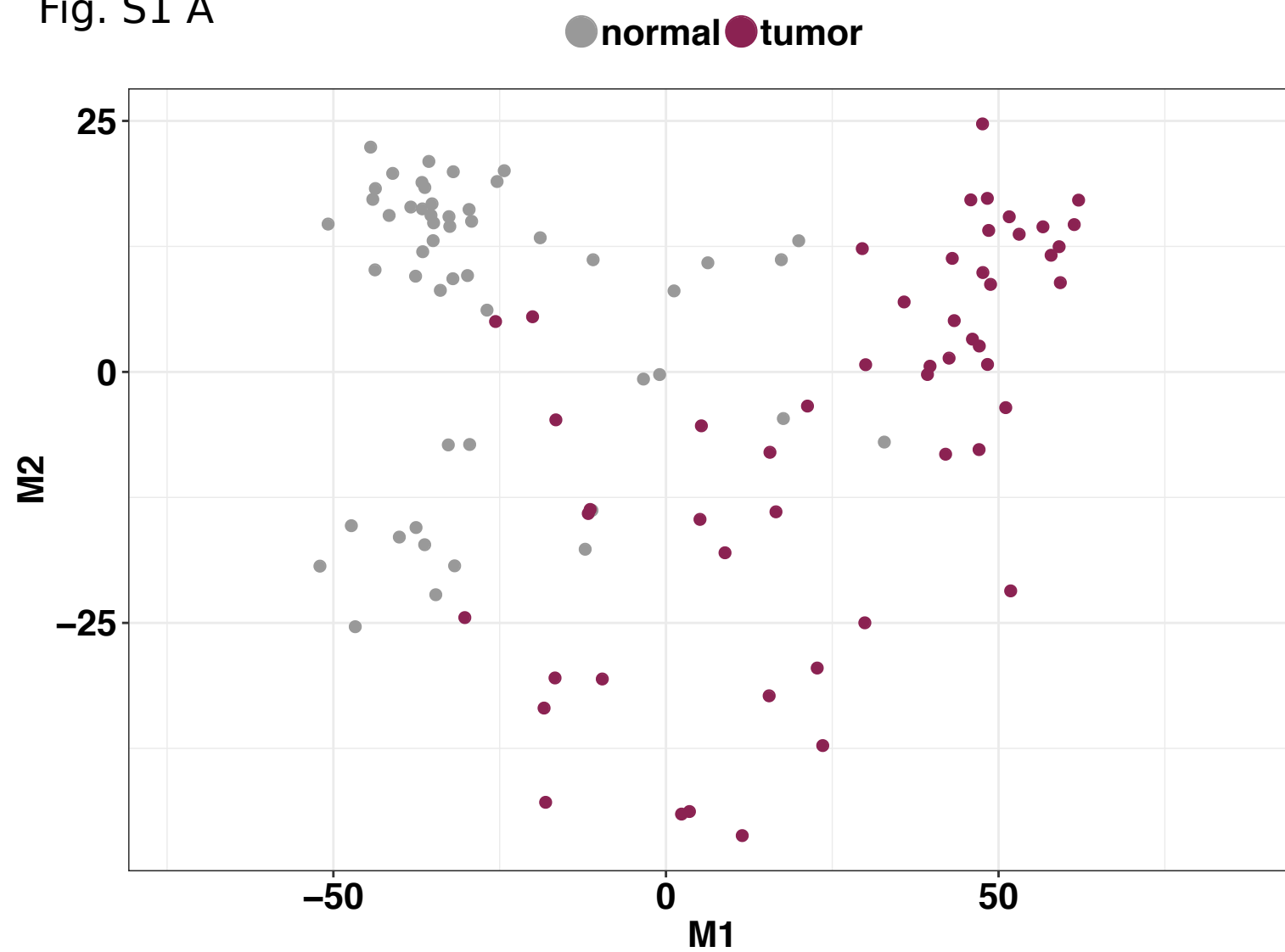

Fig. S1 B

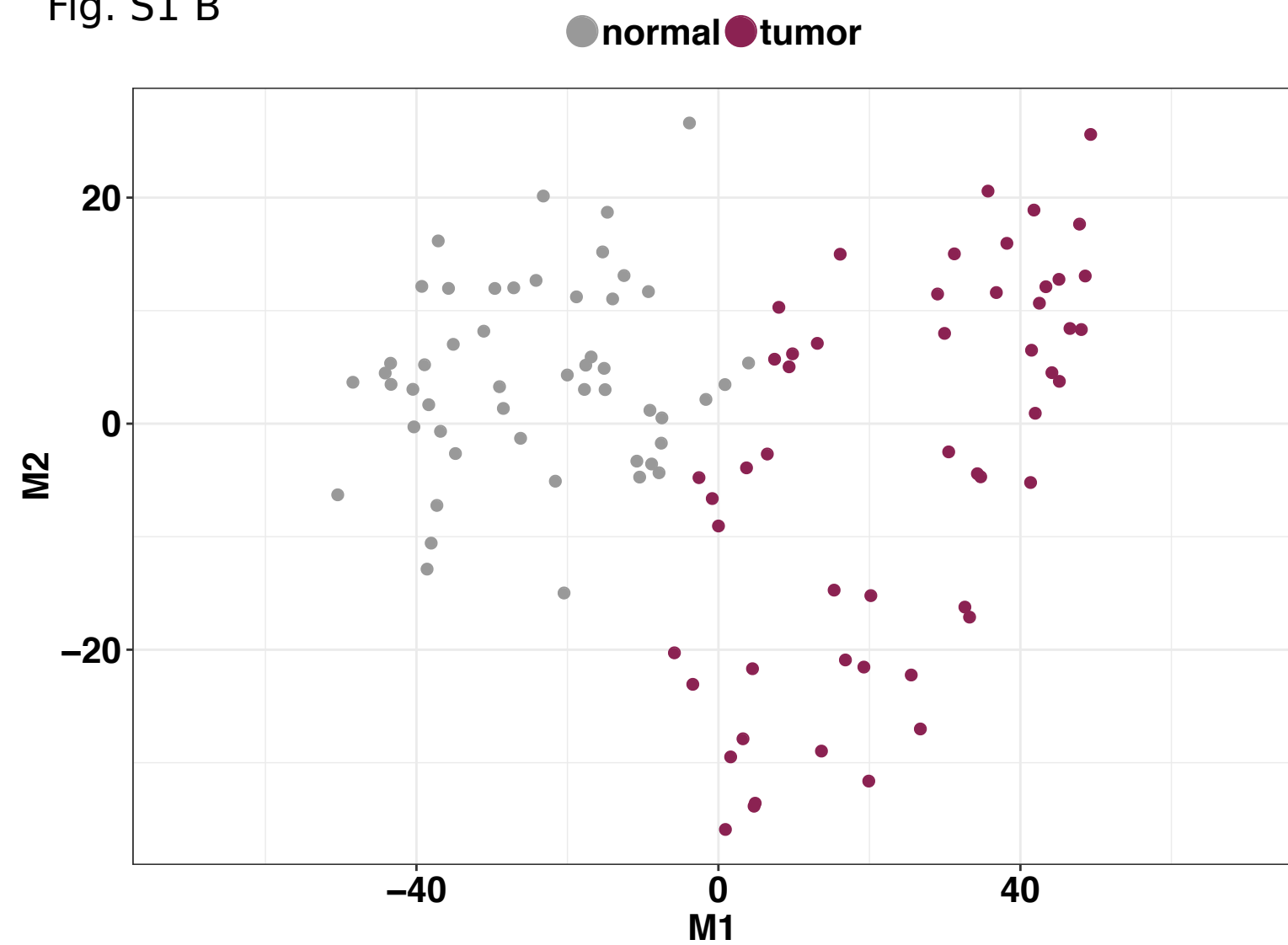

Fig. S1 C

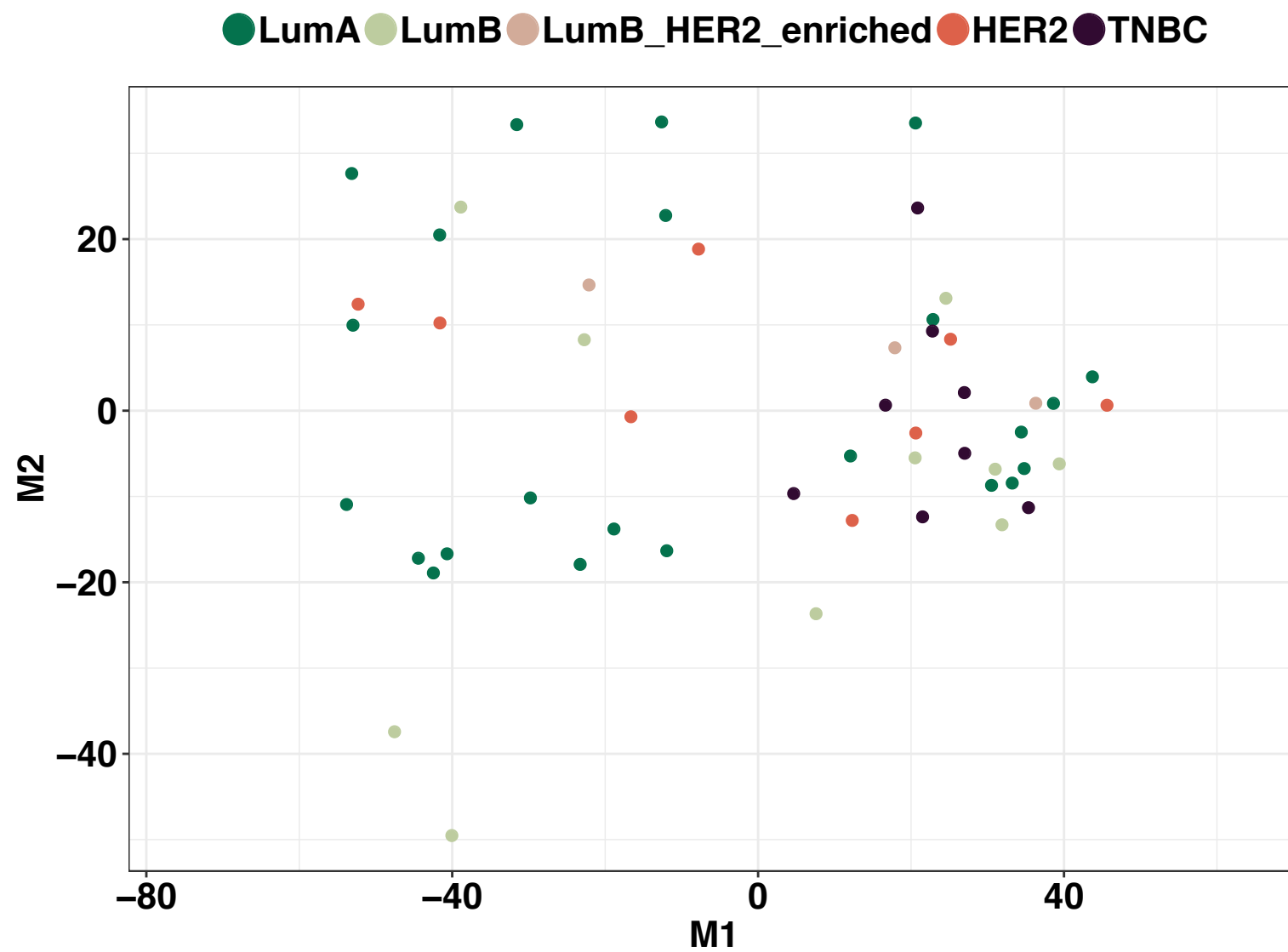

Fig. S1 D

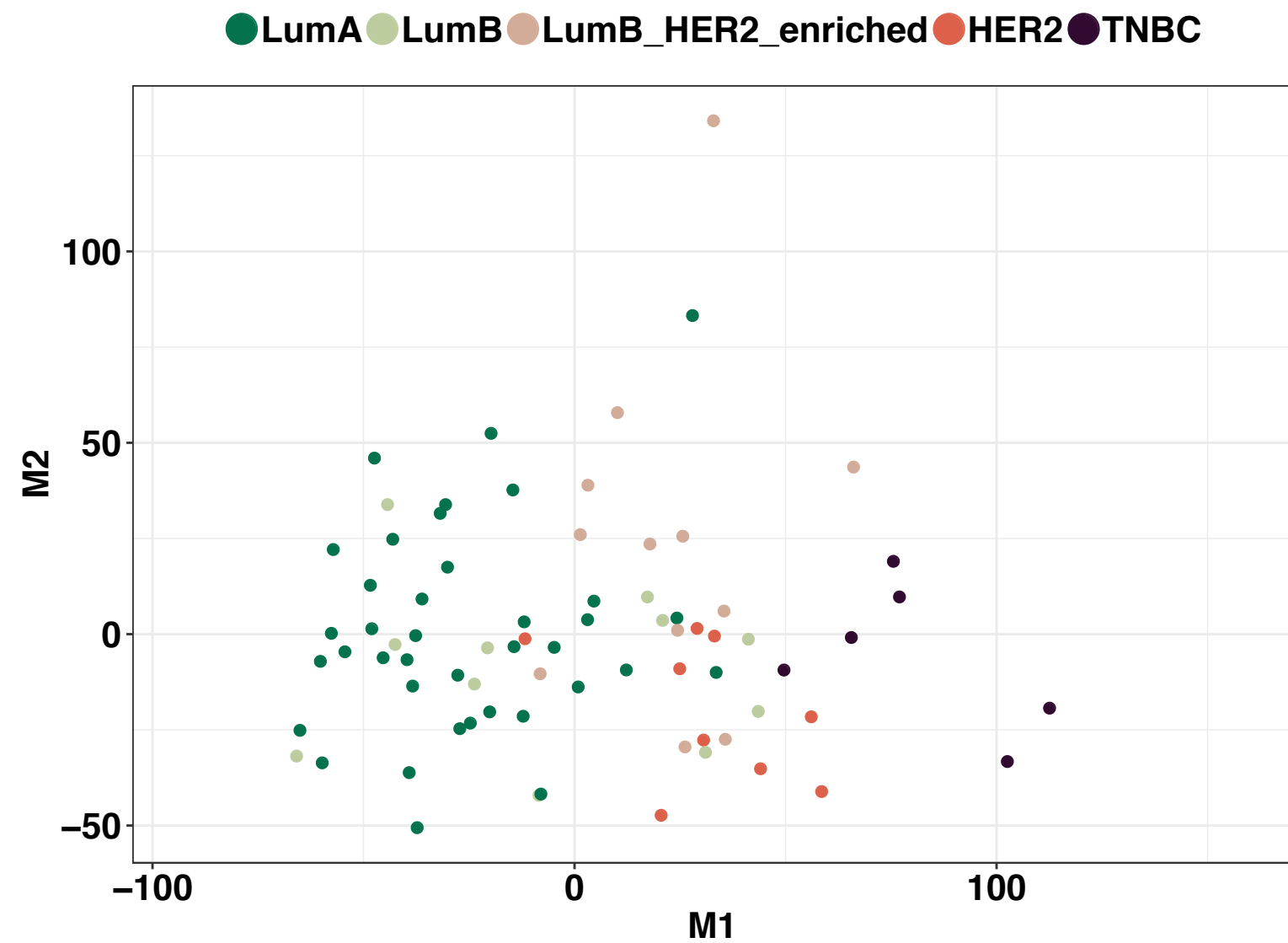

Supplement: Supplementary file 1 — Additional file 1: Figure S1. Multidimensional Scaling Plot. Plot depicts the relationship (squared euclidian distances) between breast cancer samples based on the abundance of interstitial fluid miRNA or expression of intra-tumor mRNA. S1A = clustering based on miRNA abundances in IFs before correction for patient-specific effects. Colors: grey = normal interstitial fluids, red = tumor interstitial fluids. S1B = clustering based on miRNA abundances in IFs after correction for patient specific effects (heterogeneity). Colors: grey = normal interstitial fluids, red = tumor interstitial fluids. S1C = clustering based on miRNA abundances in TIFs. Colors denote BC subtypes: dark green= luminal A, light green = luminal B, pink = luminal B Her2-enriched, orange = Her2-enriched and deep red = TNBC. S1D = clustering based on intra-tumor mRNA expression. Colors denote BC subtypes: dark green= luminal A, light green = luminal B, pink = luminal B Her2-enriched, orange = Her2-enriched and deep red = TNBC. [file 13058_2020_1295_MOESM1_ESM.pdf]

Fig. S2 A

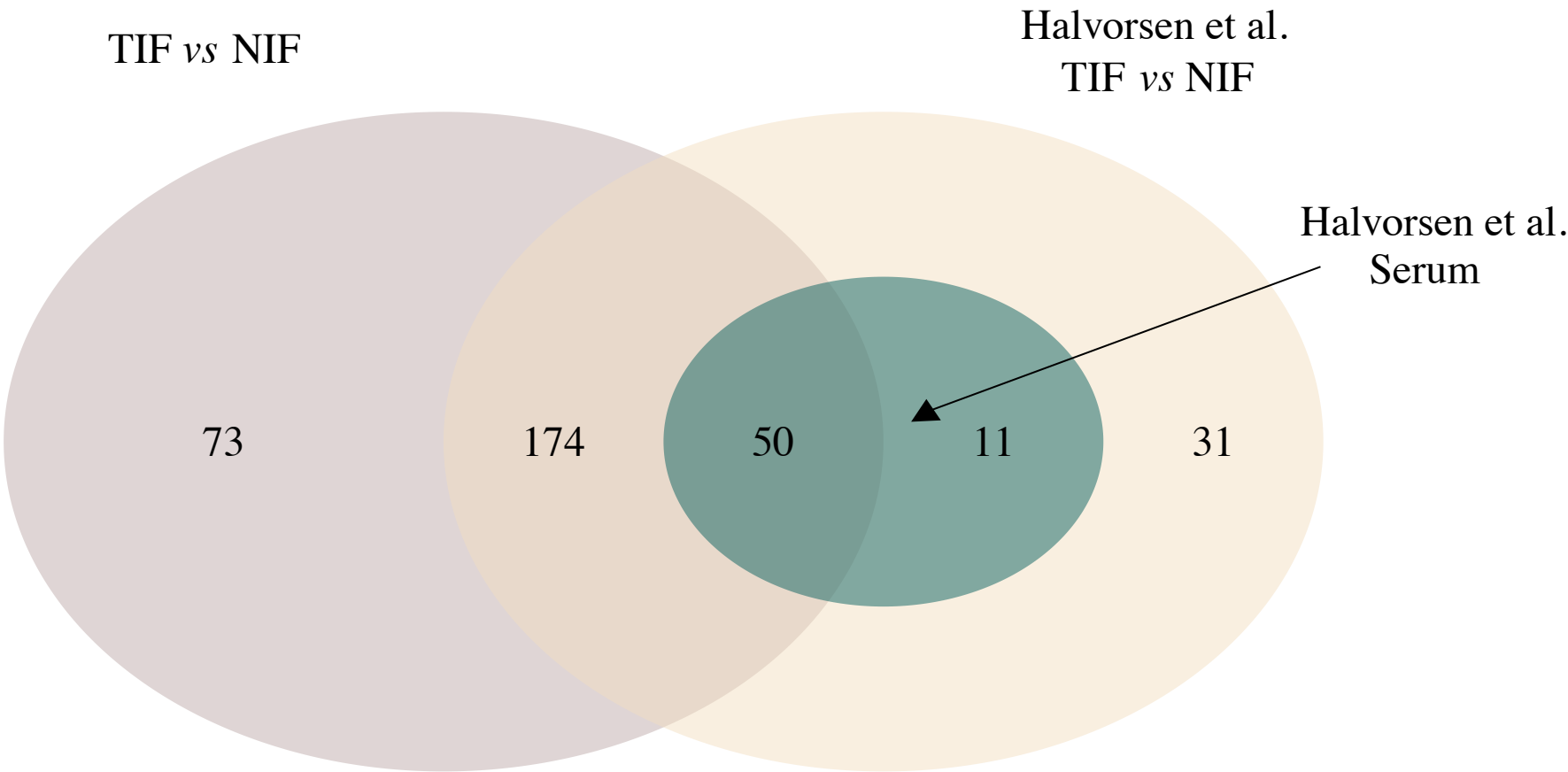

Fig. S2 B

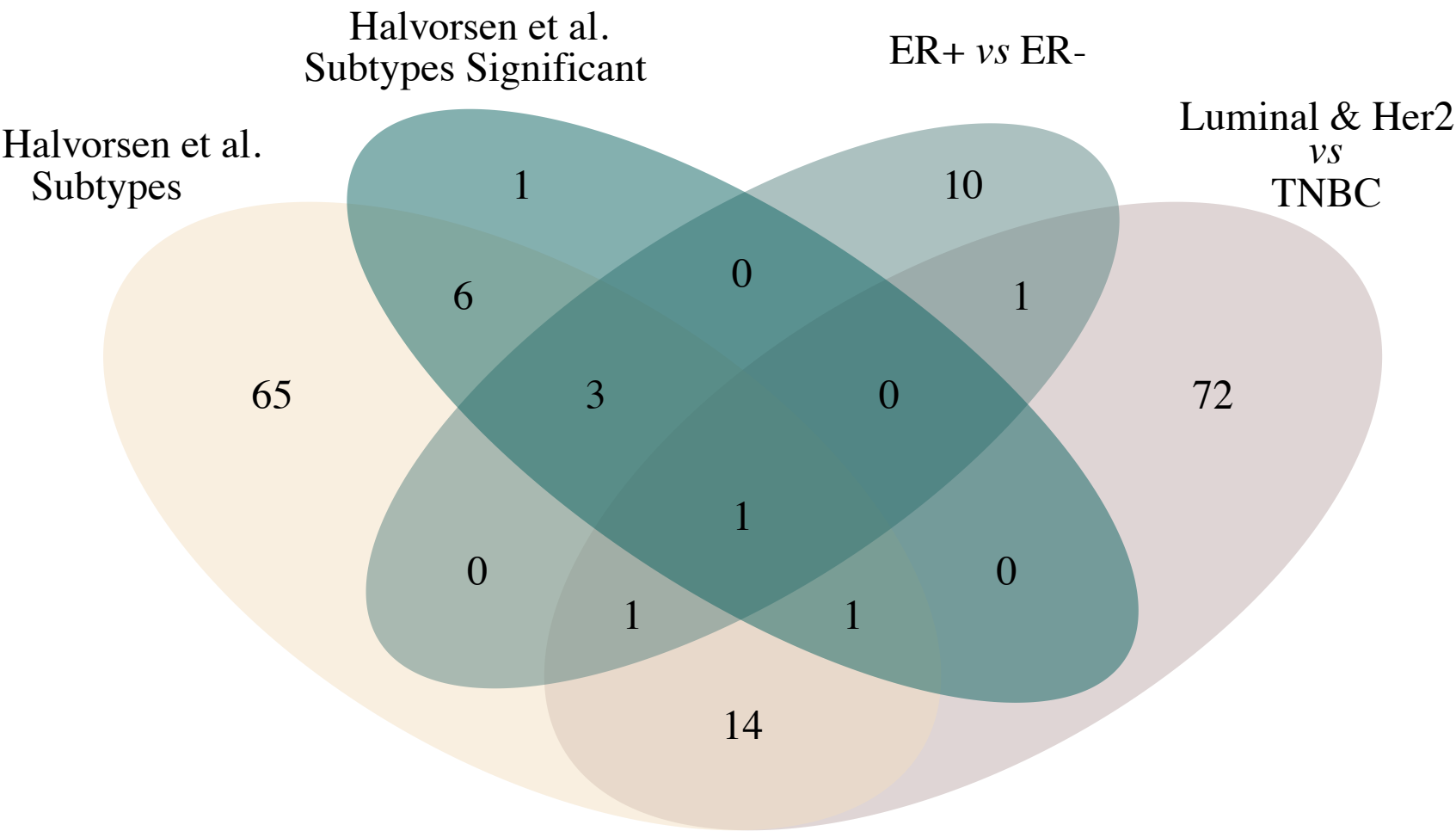

Fig. S2 C

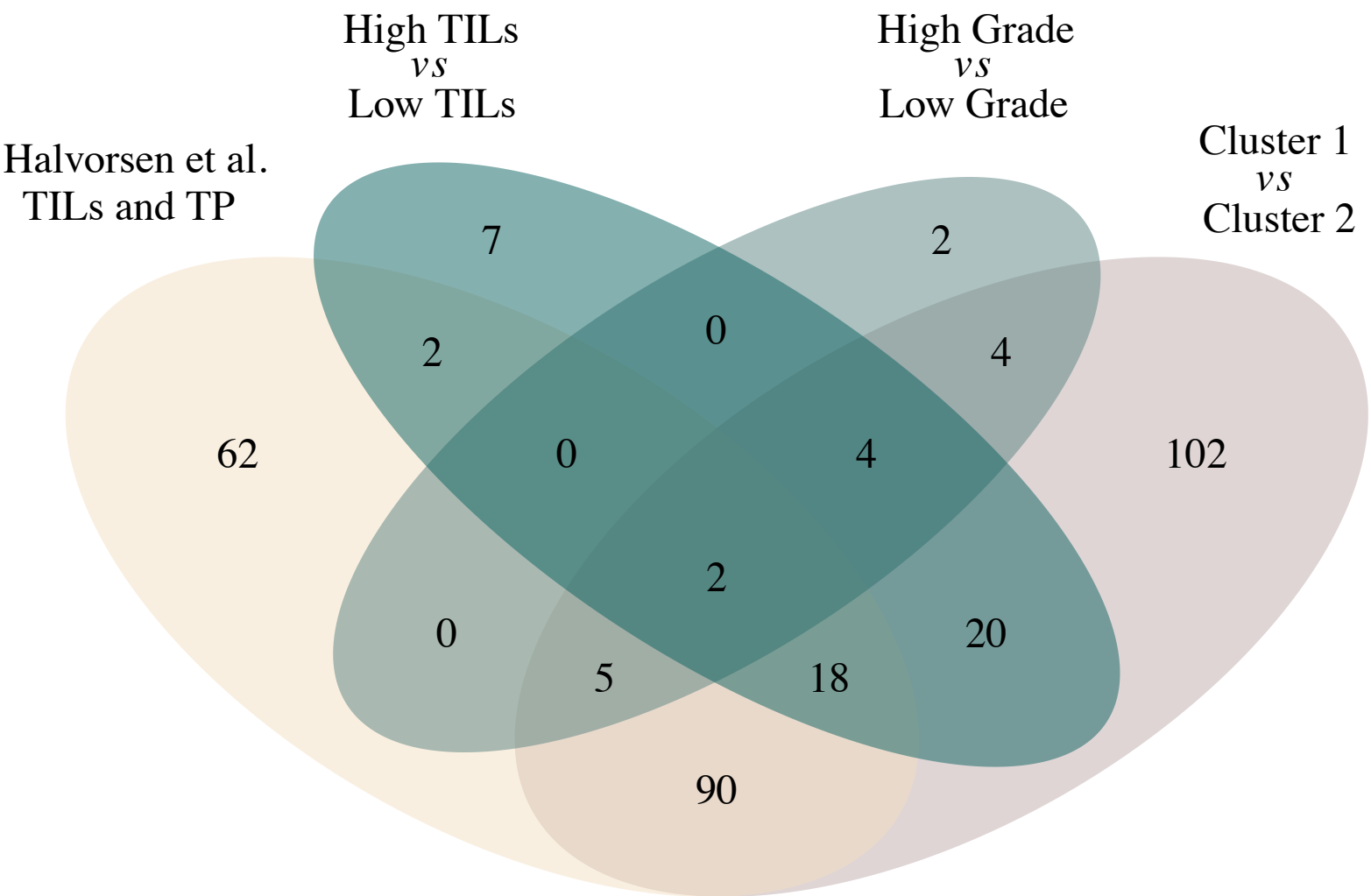

Supplement: Supplementary file 3 — Additional file 3: Figure S2. Comparison of Differentially Abundant miRNAs. Comparison of differentially abundant miRNAs from current analysis with original publication (Halvorsen, et al. 2017). S2A = Comparison of miRNAs DA in TIF vs NIF, and expressed in paired serum, set include (i) miRNAs DA between TIF vs NIF, from Halvorsen, et al. 2017, (ii) miRNAs DA between TIF vs NIF, also in serum, from Halvorsen, et al. 2017, and (iii) miRNAs DA between TIF vs NIF from current analysis. S2B = Comparison of miRNAs DA between BC subtypes. Sets include (i) miRNAs DA between subtypes, from Halvorsen, et al. 2017, (ii) miRNAs DA between subtypes significant after correction for multiple testing, from Halvorsen, et al. 2017, (iii) miRNAs DA between subtypes significant from current analysis and (iv) miRNAs DA between ER+ and ER- from current analysis. S2C = Comparison of miRNAs associated with the degree of tumor infiltrating lymphocytes. Sets include (i) miRNAs associated with TILs and tumor percentage, from Halvorsen, et al. 2017, (ii) miRNAs DA between high (+2|+3) vs low TILs (0/1) from current analysis. (iii) miRNAs DA between high (gr 3) vs low/medium tumor grade (gr 1|2) from current analysis, and (iv) miRNAs DA between TIF Cluster 1 vs Cluster 2 from current analysis. [file 13058_2020_1295_MOESM3_ESM.pdf]

Fig. S4

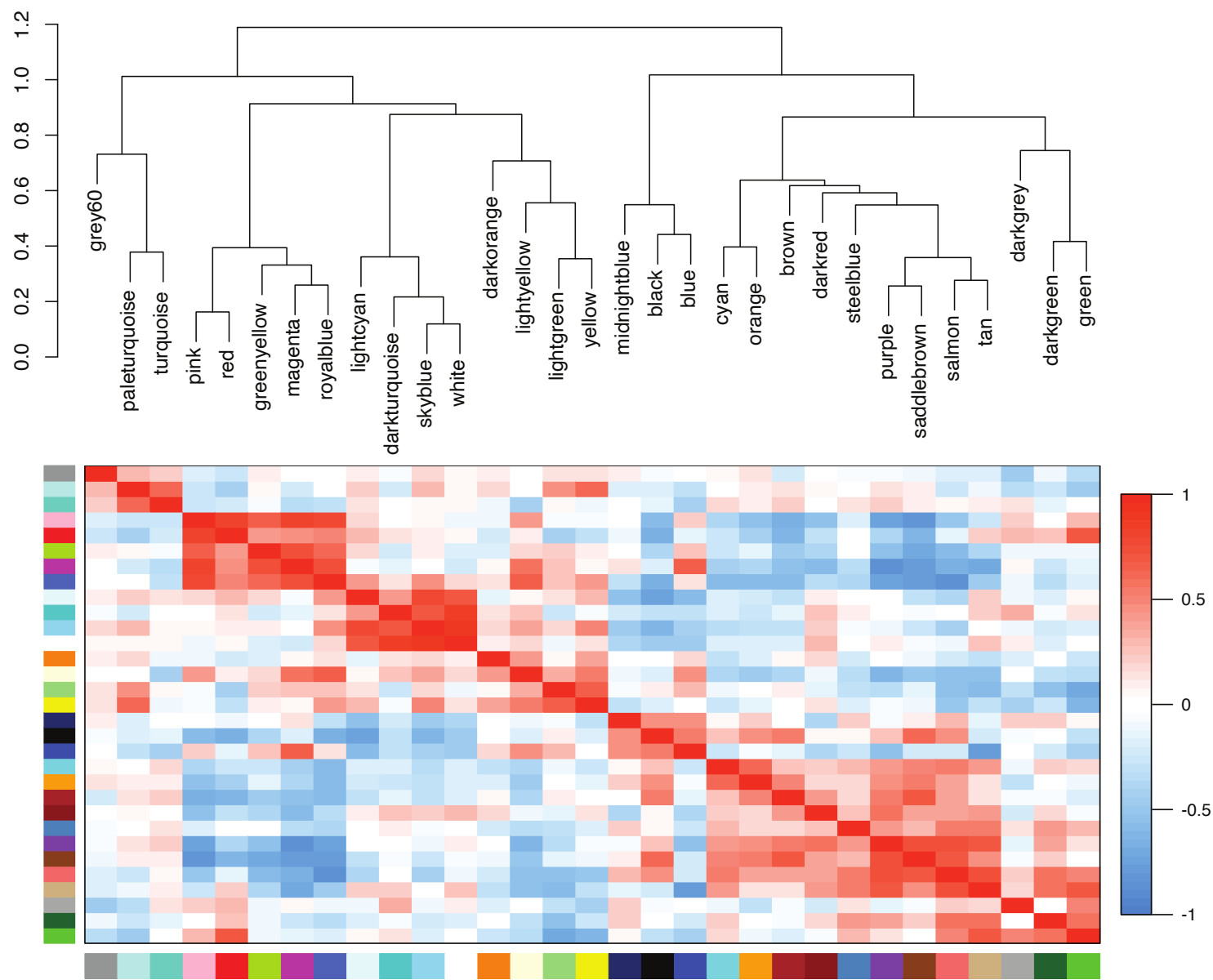

Supplement: Supplementary file 7 — Additional file 7: Figure S4. Module Relationships. The hierarchical clustering and heatmap show how similar the modules are (correlation scale on the side). The color assignment is reported as well on the X and Y axes. [file 13058_2020_1295_MOESM7_ESM.pdf]

**Fig. S5**

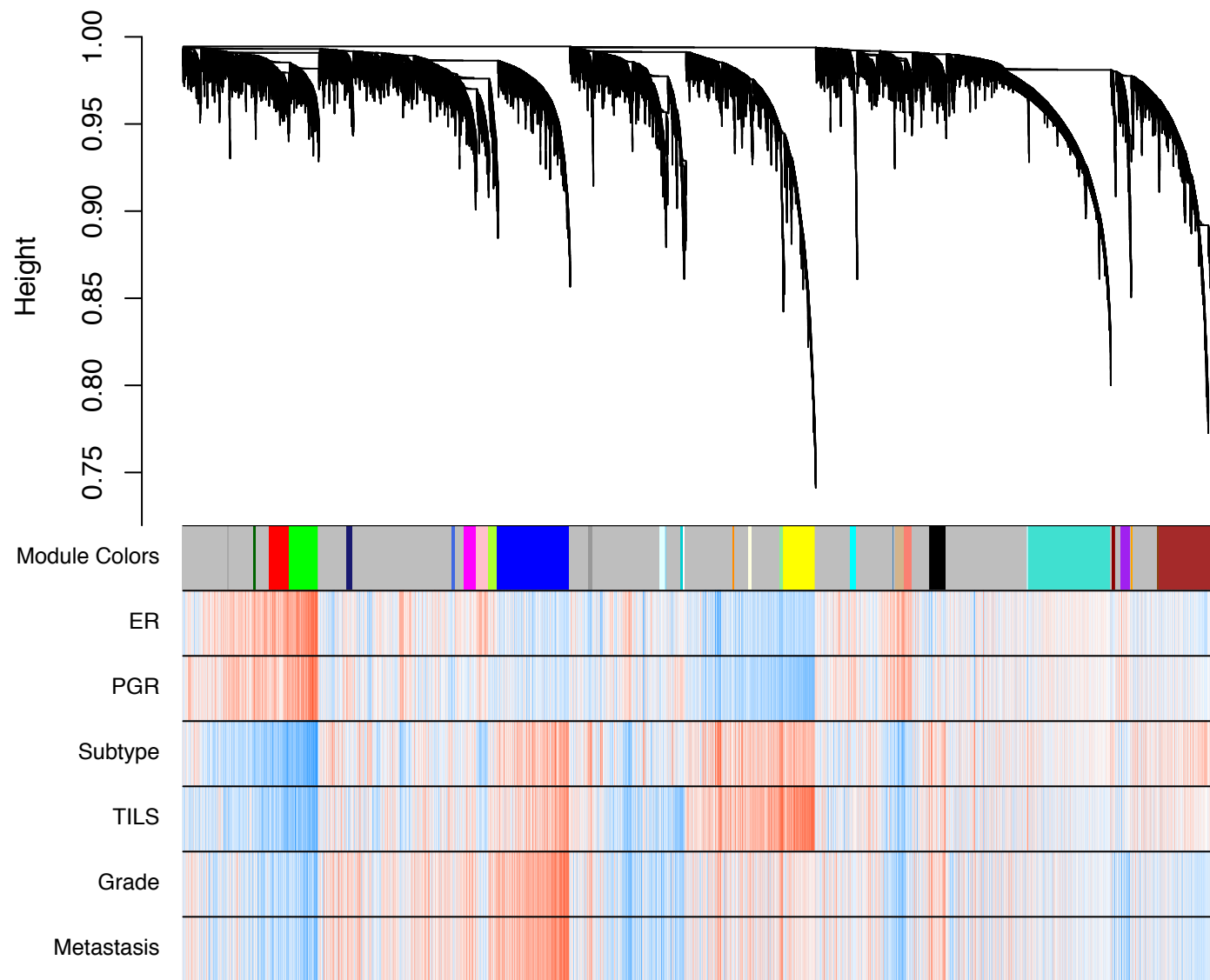

Supplement: Supplementary file 9 — Additional file 9: Figure S5. Intra-tumor mRNA Co-expression Modules. Results of weighed Gene Co-expression Network Analysis. Upper part of plot shows the clustering of the genes co-expressed in the 31 modules from Weighed Gene Co-expression Network Analysis (WGCNA). Grey denotes that the gene was not assigned to any module. Modules are named by their color. Lower part of plot shows the correlation between patient clinical variables and modules. [file 13058_2020_1295_MOESM9_ESM.pdf]

Fig. S6

Fig. S6 A

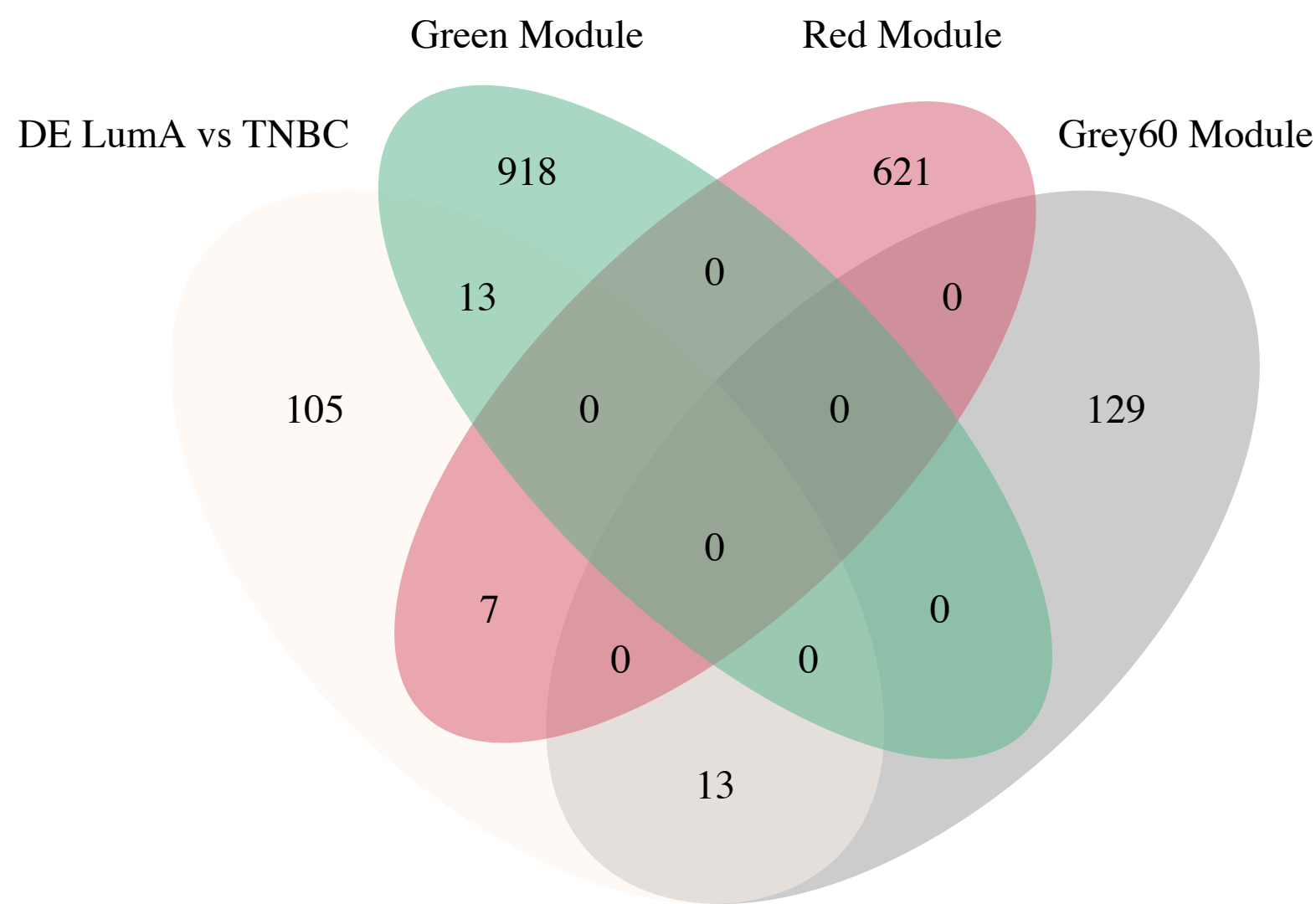

Fig. S6 B

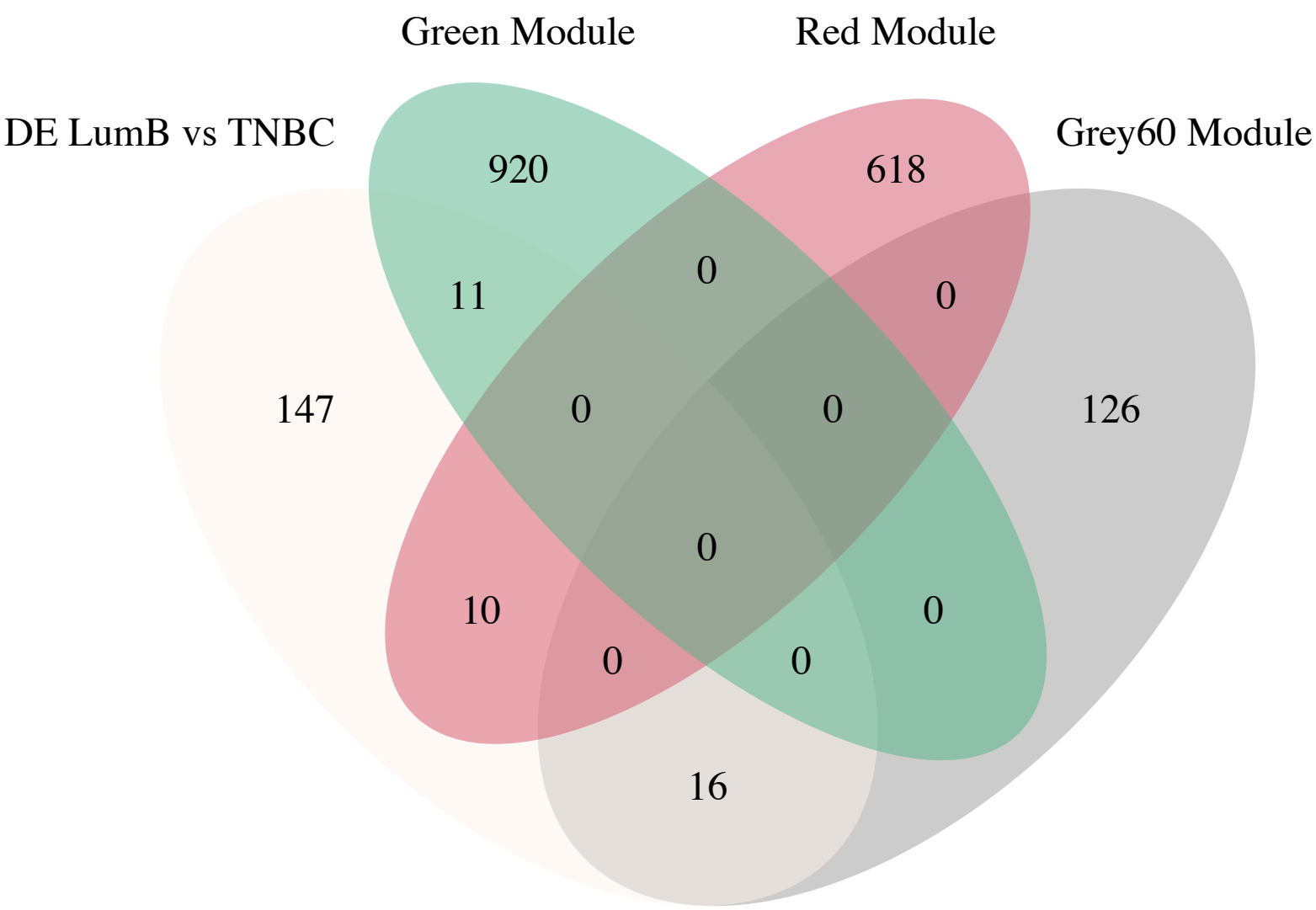

Fig. S6 C

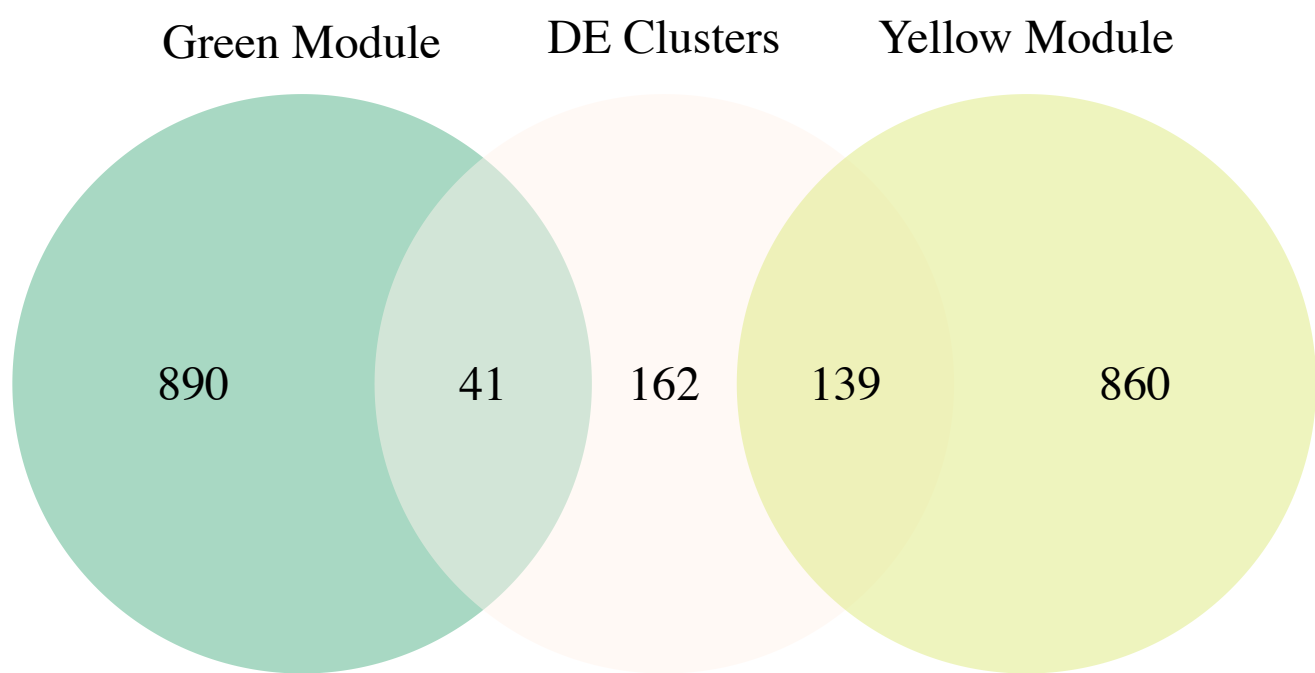

Fig. S6 D

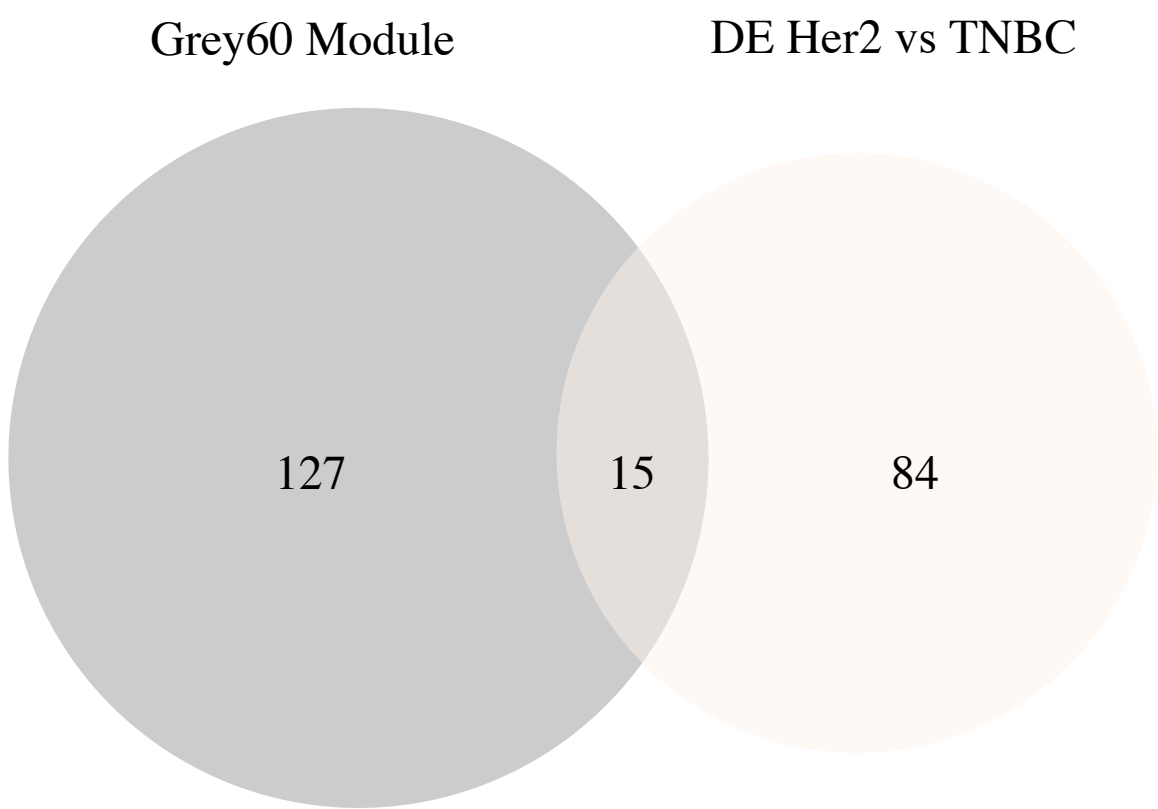

Fig. S6E

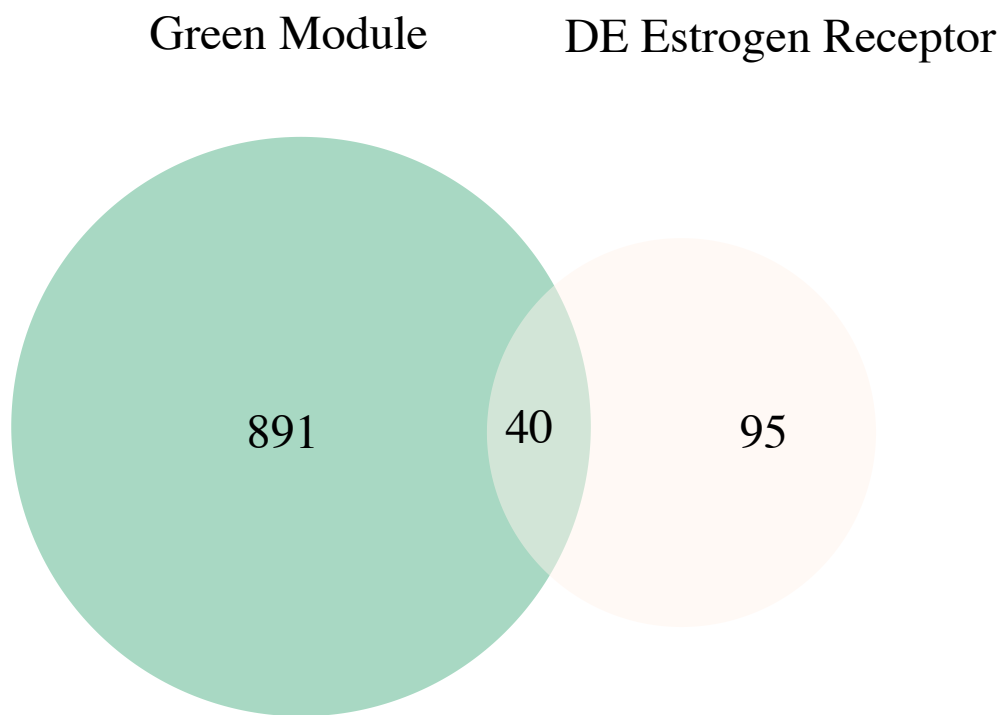

Fig. S6 F

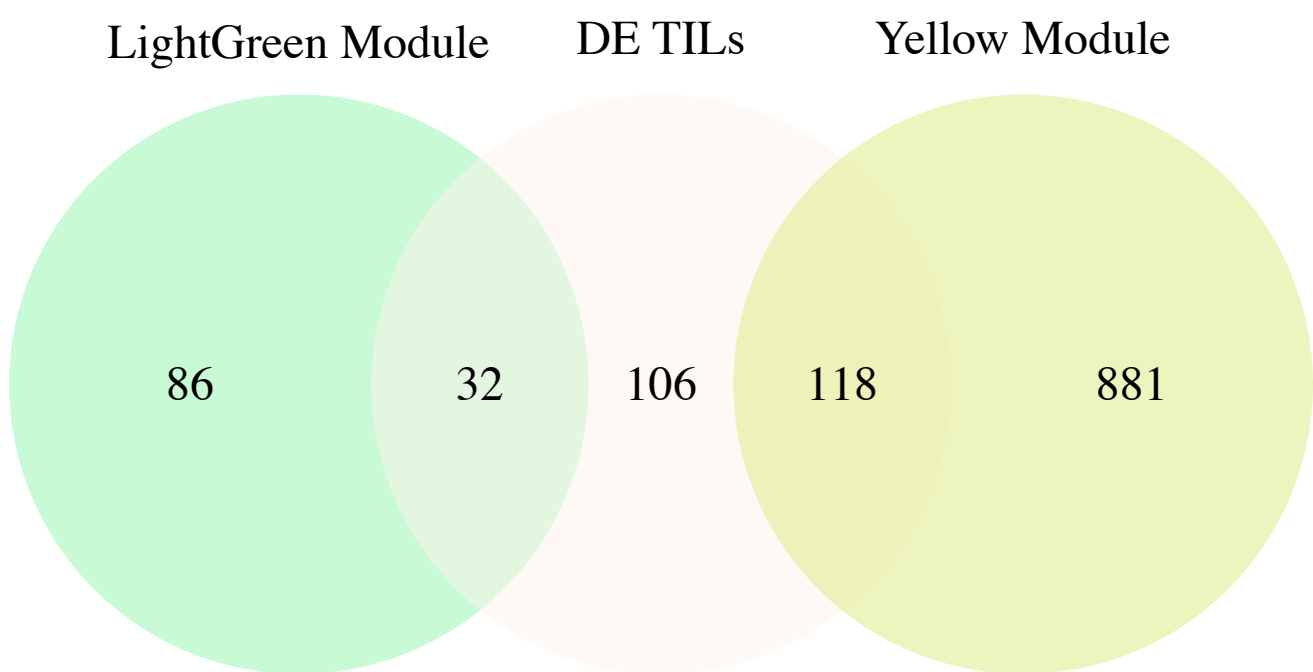

Fig. S6G

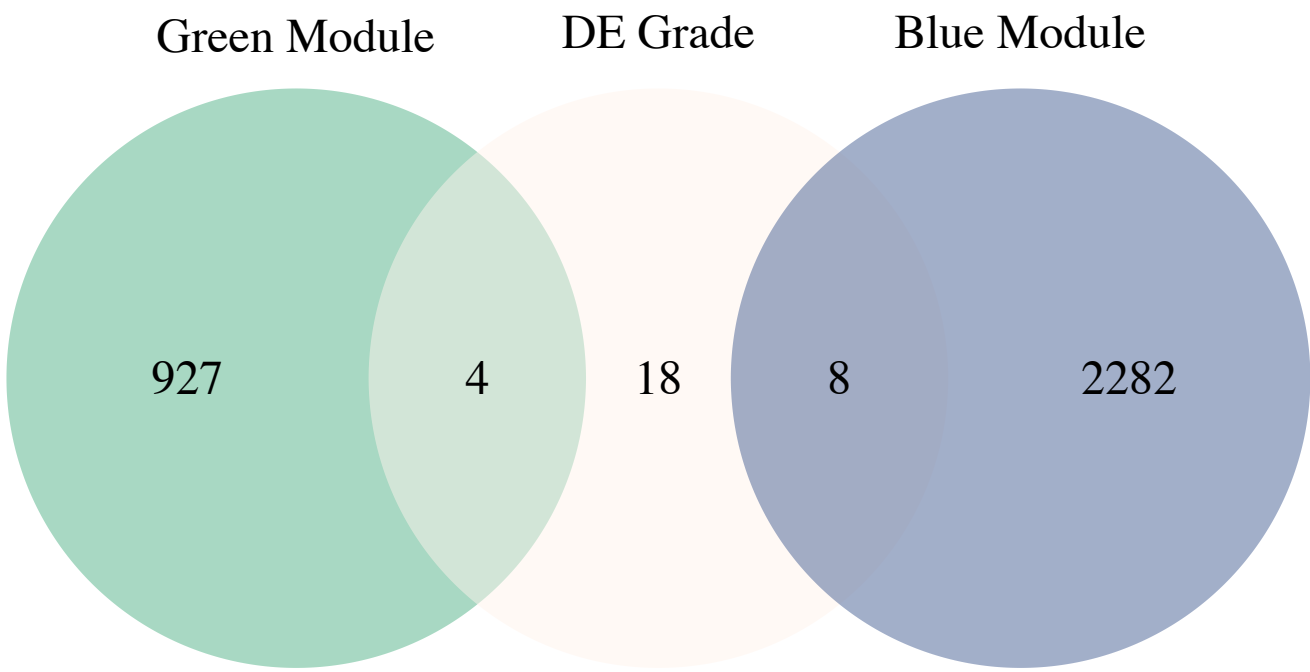

Supplement: Supplementary file 10 — Additional file 10: Figure S6. Overlap of Differentially Expressed mRNAs with Co-expressed mRNAs. Venn diagrams, depicting the overlap between differentially expressed mRNAs from contrasts, with modules, which were correlated with the patient clinical feature of interest. [file 13058_2020_1295_MOESM10_ESM.pdf]

Fig. S7

Fig. S7 A

Subtypes

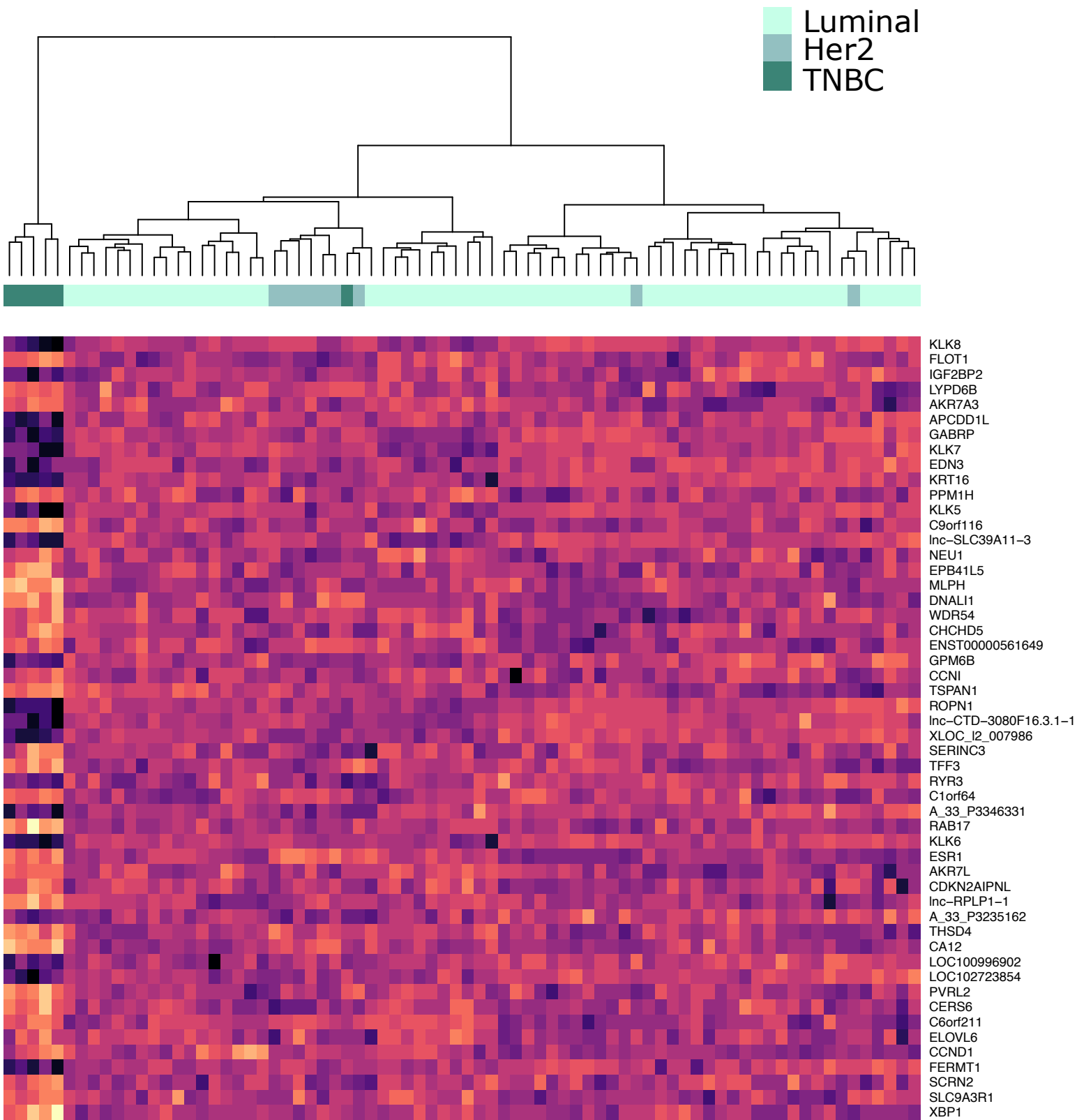

Fig. S7 C

Grade / Clusters

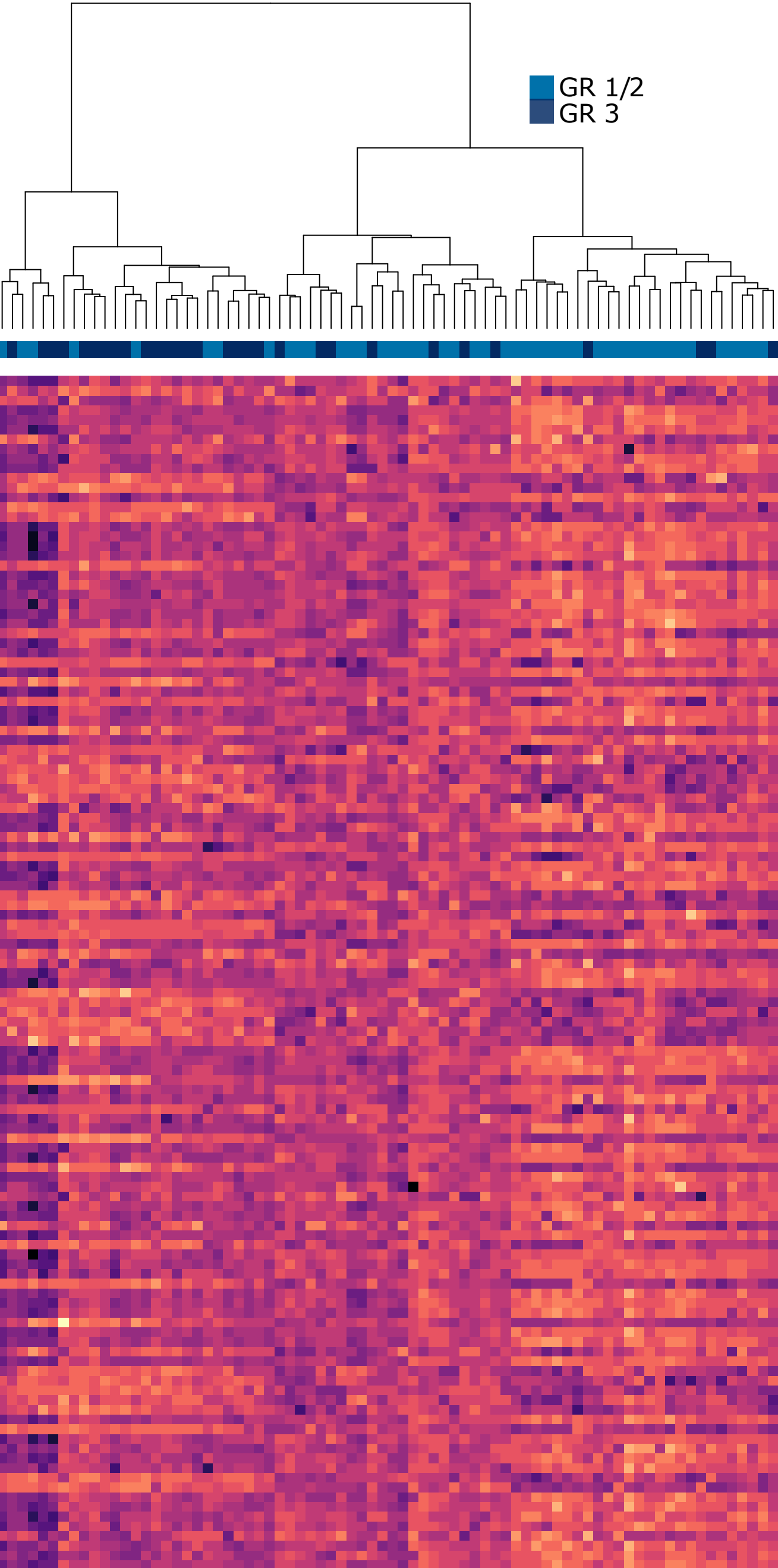

Fig. S7 B

TIL Score

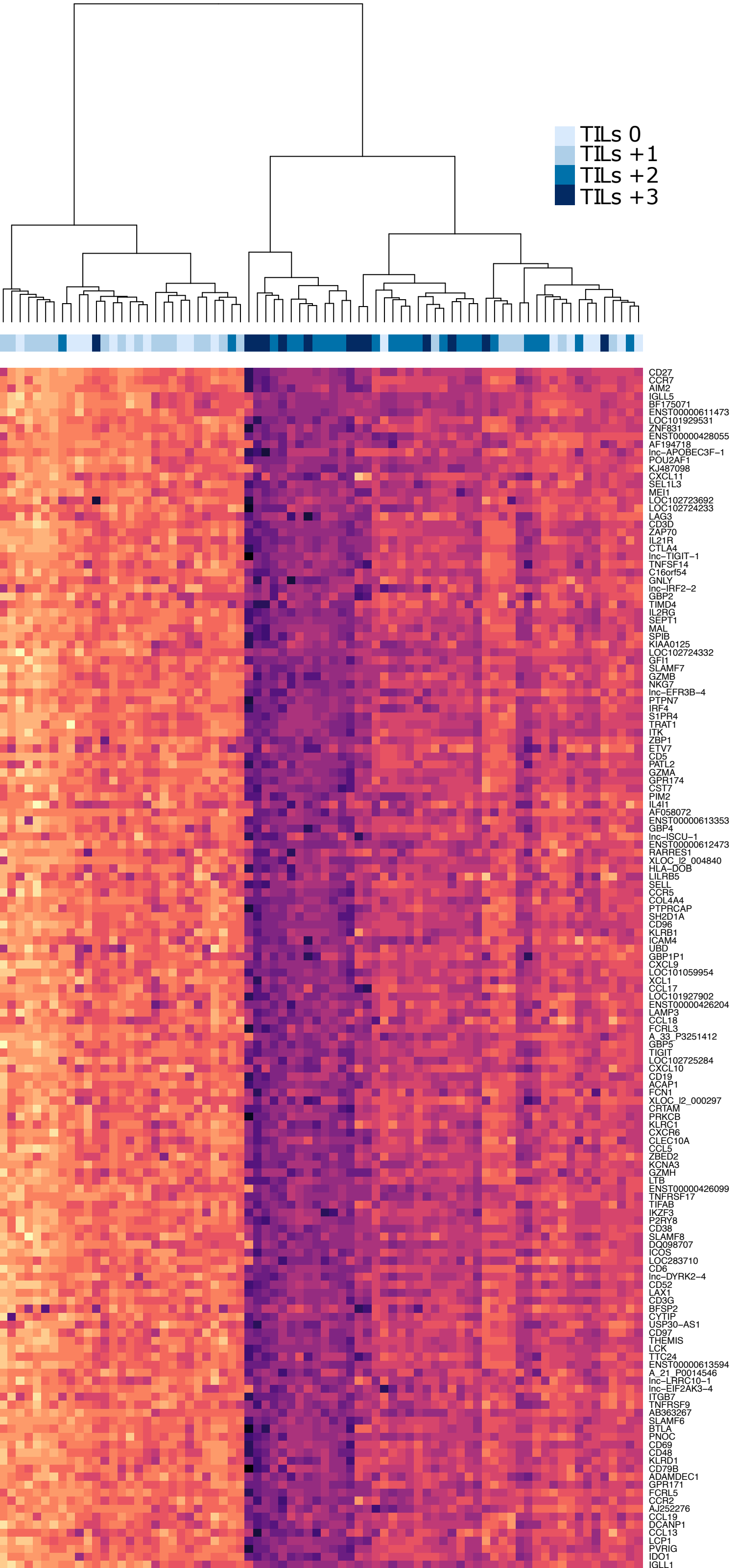

Fig. S7 D

Estrogen Receptor

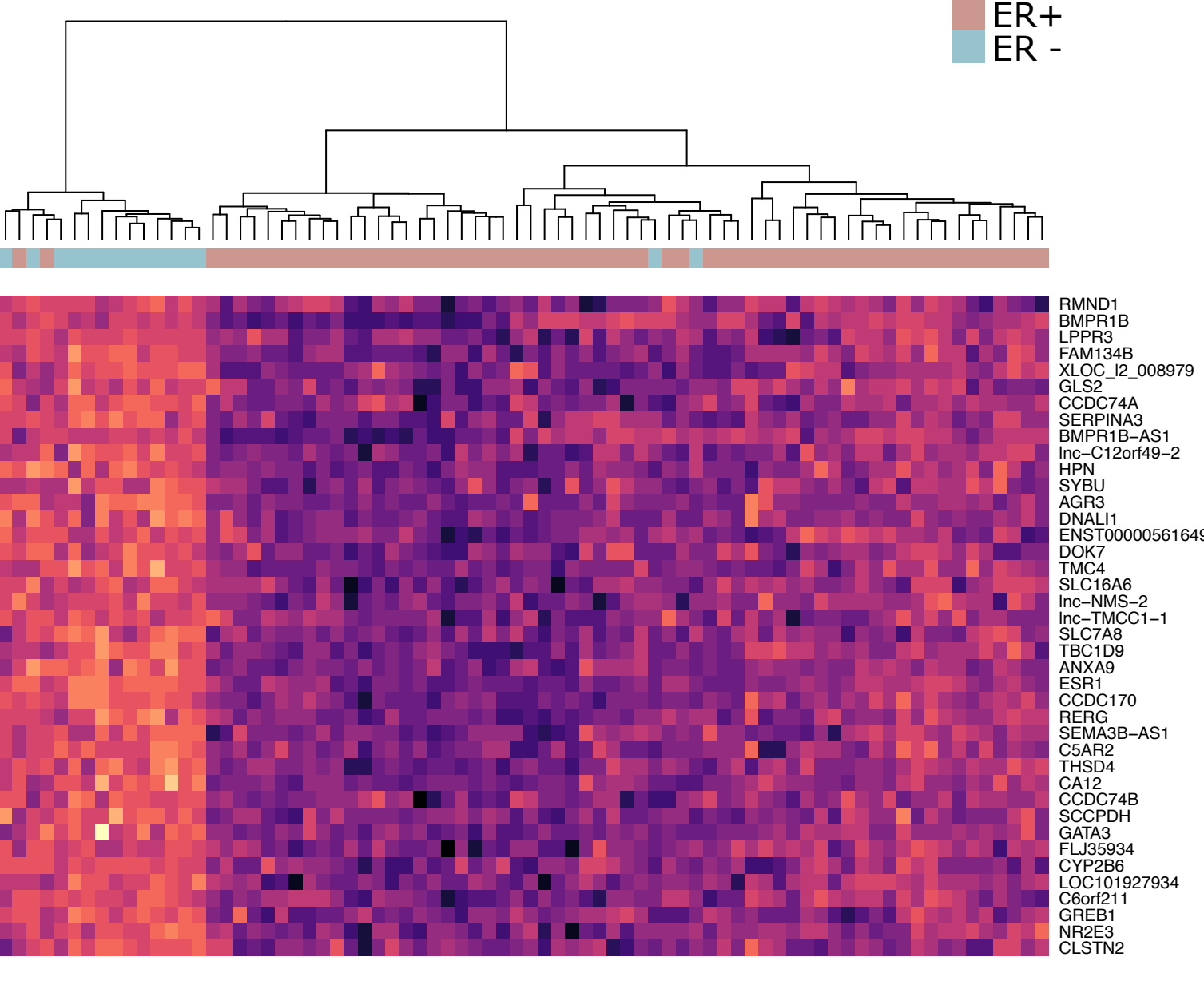

Supplement: Supplementary file 11 — Additional file 11: Figure S7. Heatmaps of DE intra-tumor mRNAs levels. Heatmaps showing the separation of tissue samples, based on the best DE mRNA candidates from Weighed Gene Co-expression Network Analysis (WGCNA). S7.A: luminal (A + B), Her2-enriched vs TNBC, S7.B: TIL scores, S7.C: tumor grade (plus clusters minus genes from the TILs comparison) and S7.D: estrogen receptor status. Color scale denotes expression levels, purple = high expression, and yellow = low expression. [file 13058_2020_1295_MOESM11_ESM.pdf]
