# Supplementary material for: Secreted breast tumor interstitial fluid microRNAs and their target genes are associated with triple-negative breast cancer, tumor grade, and immune infiltration
Source: Breast Cancer Res. 2020 Jun 30;22:73. doi: 10.1186/s13058-020-01295-6 (PMC7329449; doi:10.1186/s13058-020-01295-6)

Fig. S3 A - Cluster 1 vs Cluster 2

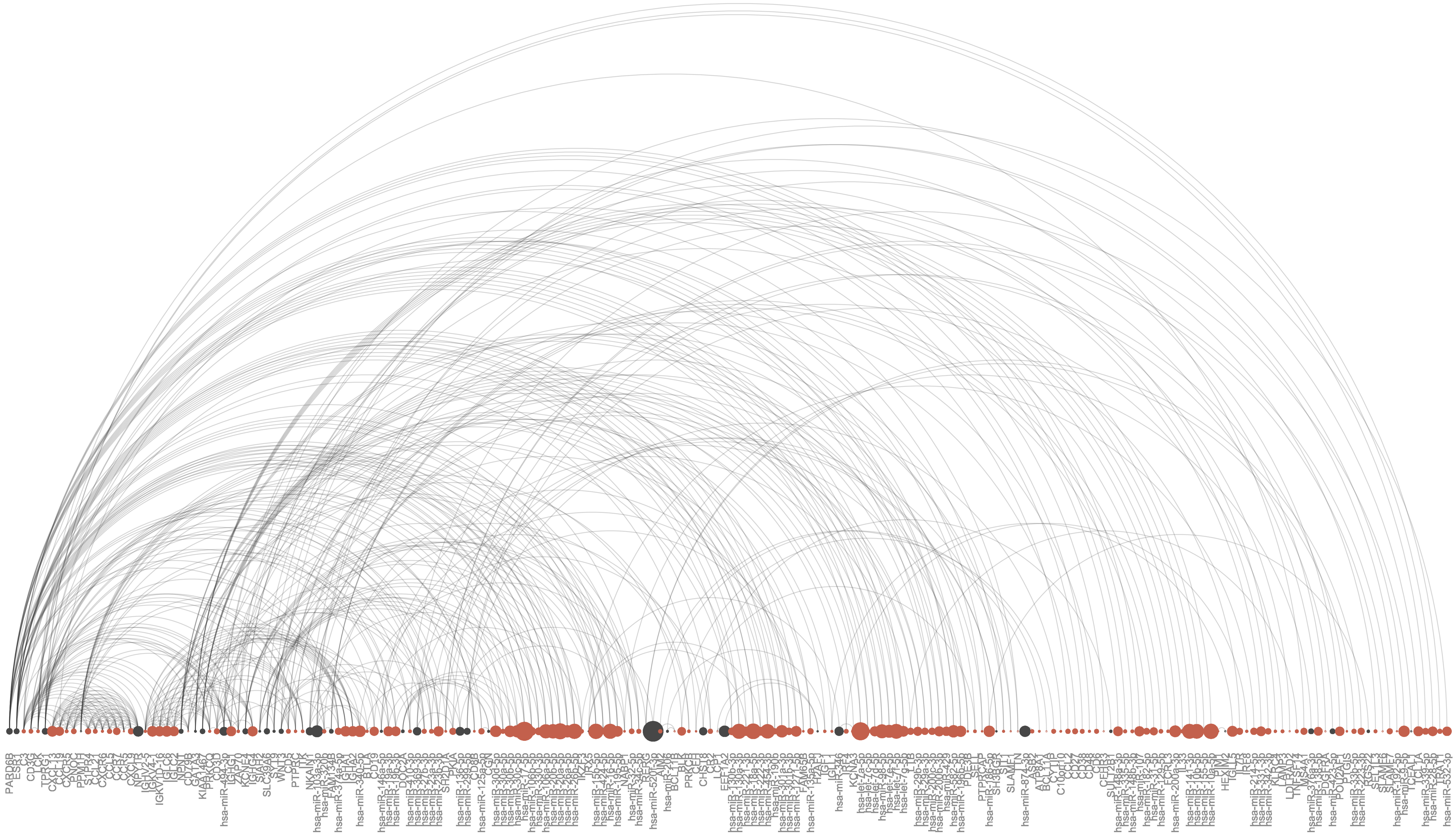

Fig. S3 B - High TILs vs Low TILs

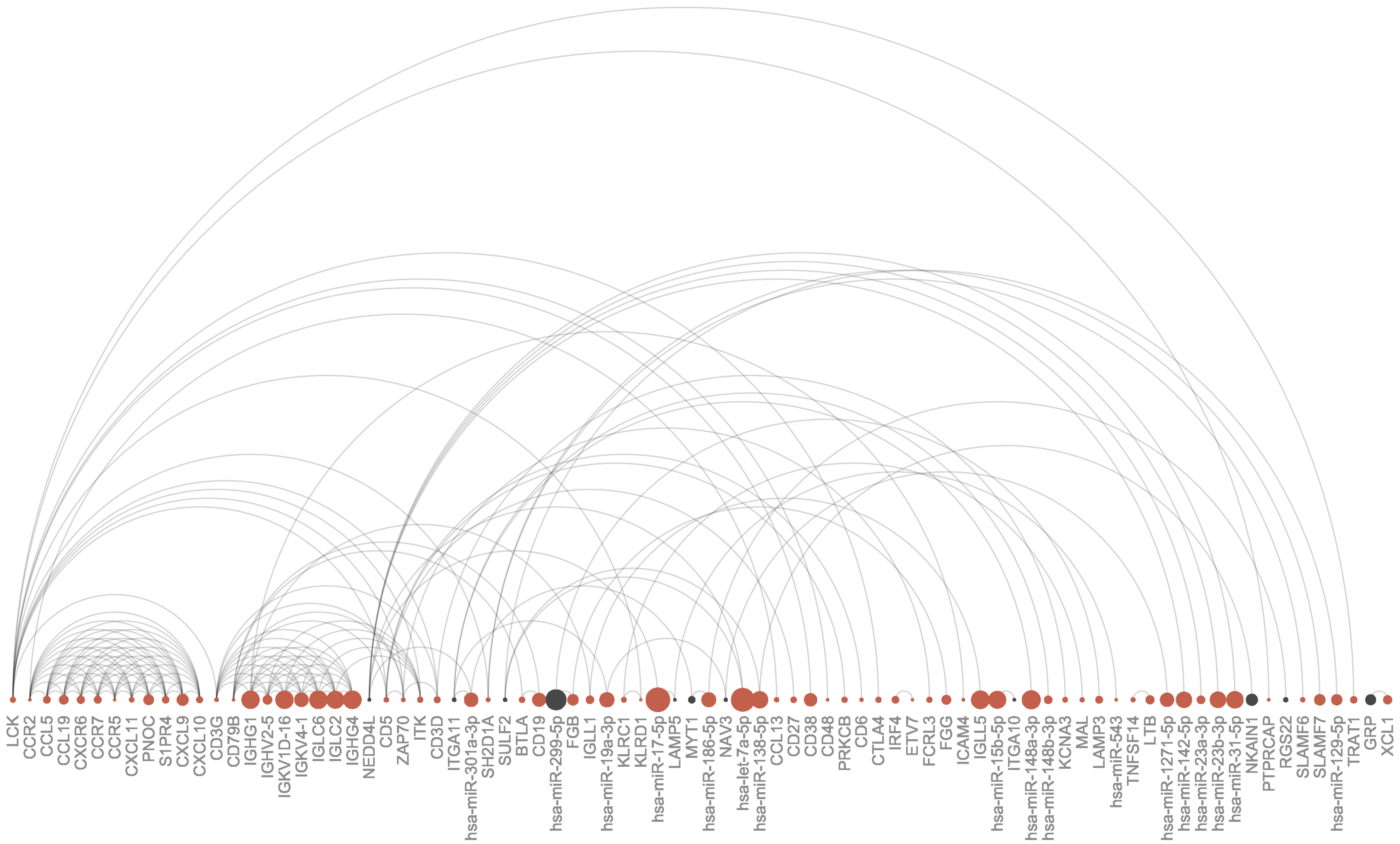

Fig. S3 C - High grade vs Medium/Low Grade

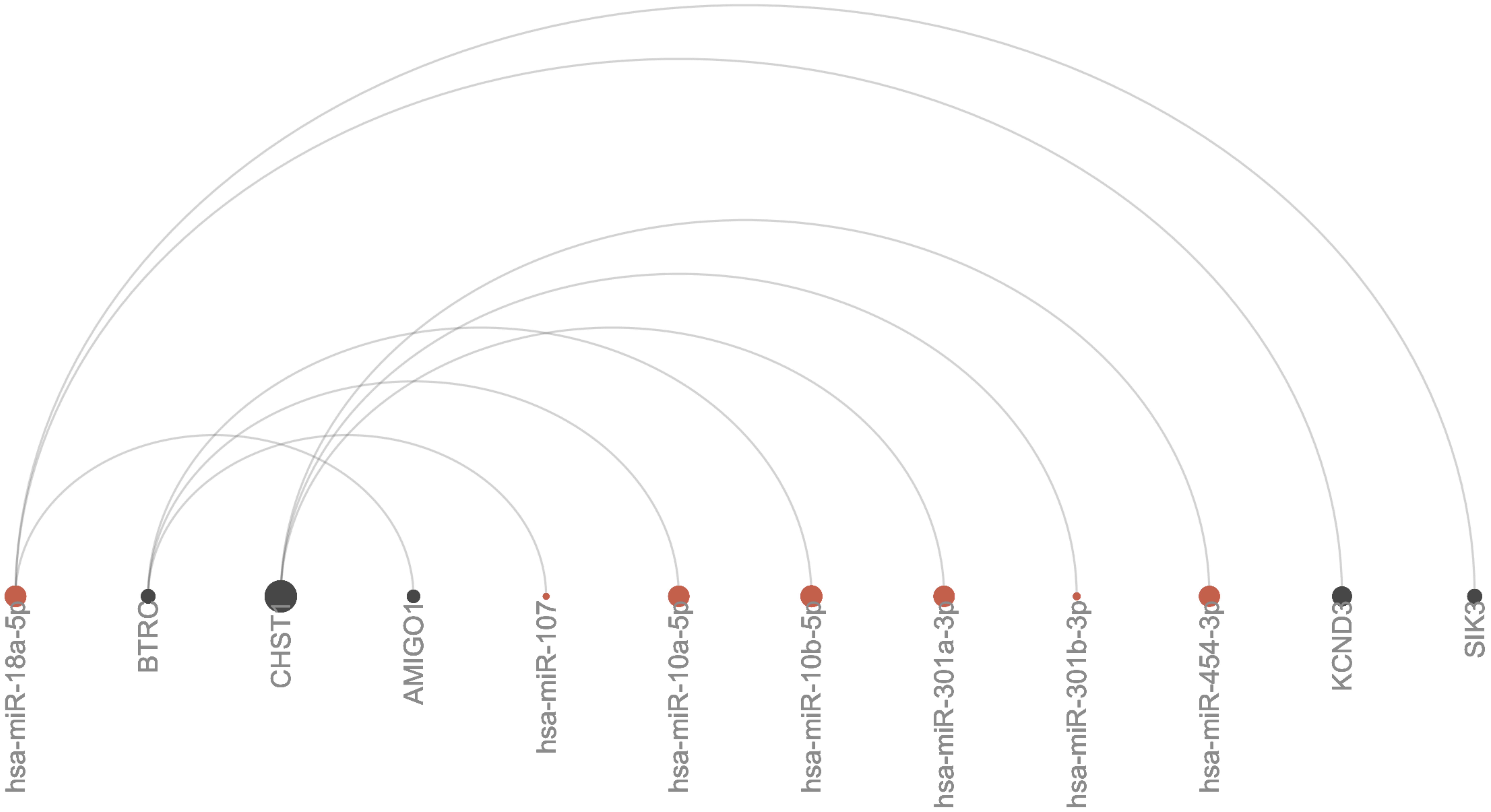

Fig. S3 D - Her2-enriched vs TNBC

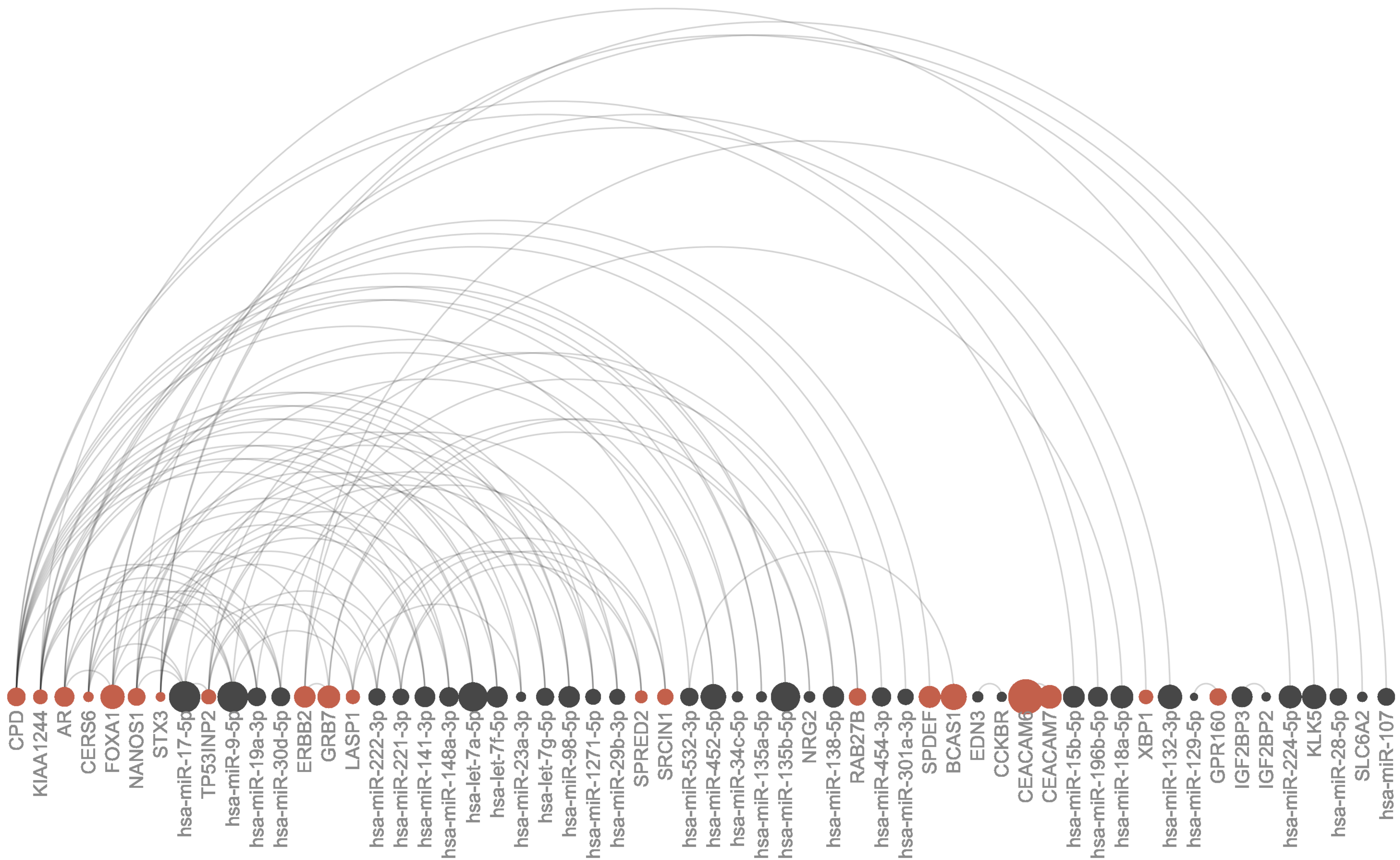

Fig. S3 E - Luminal A vs TNBC

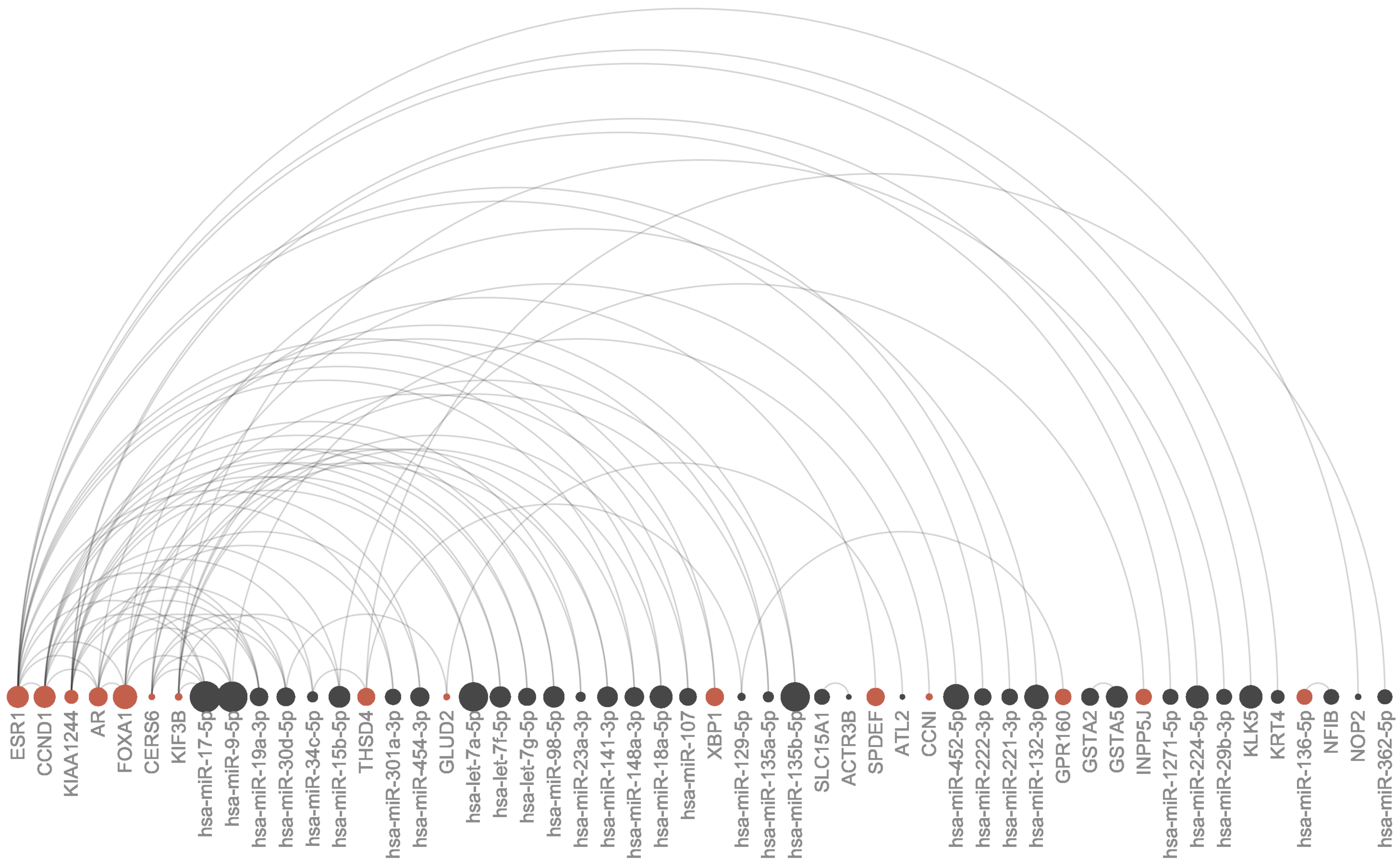

Fig. S3 F - Luminal B vs TNBC

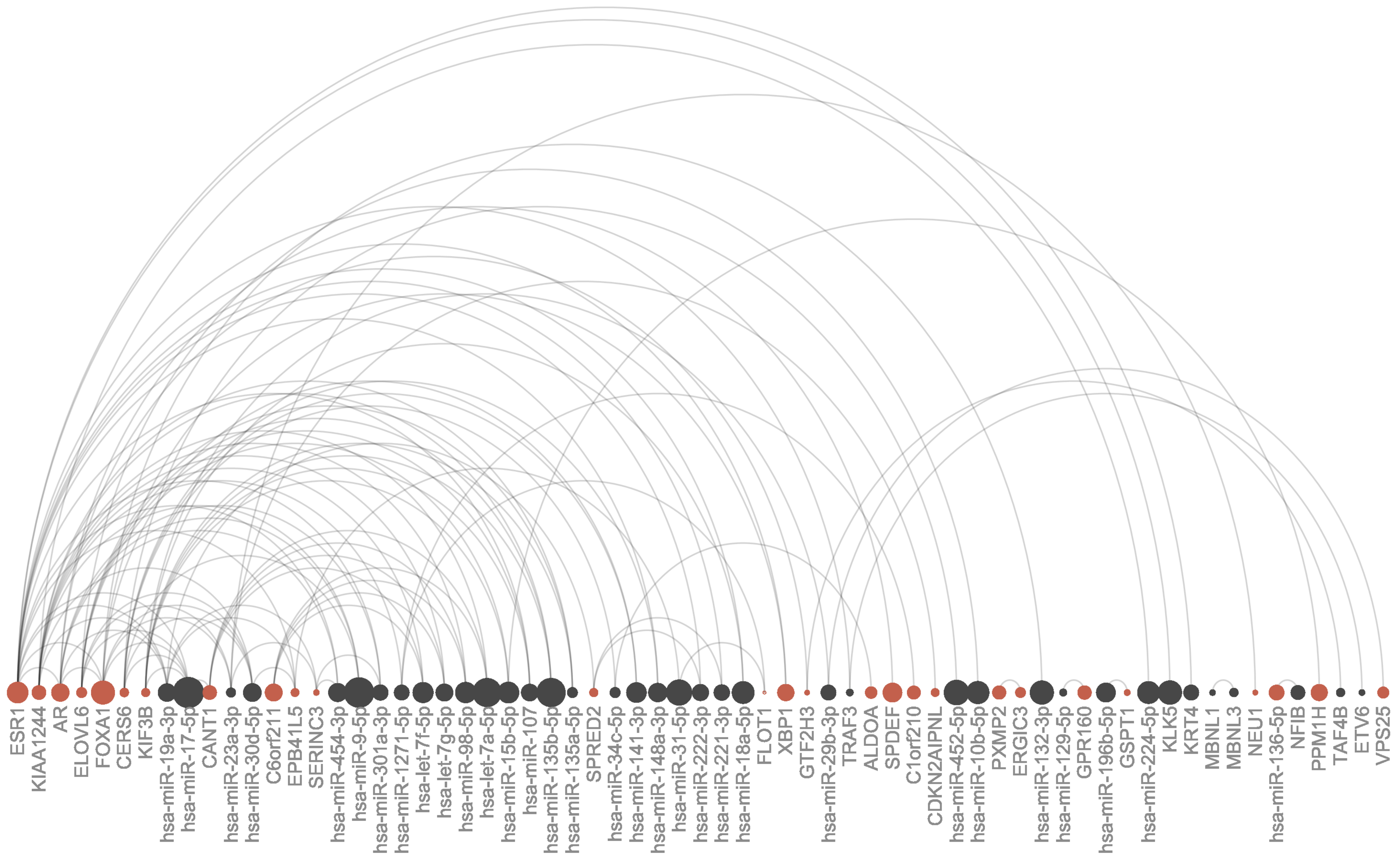

Fig. S2 G - ER+ vs ER-

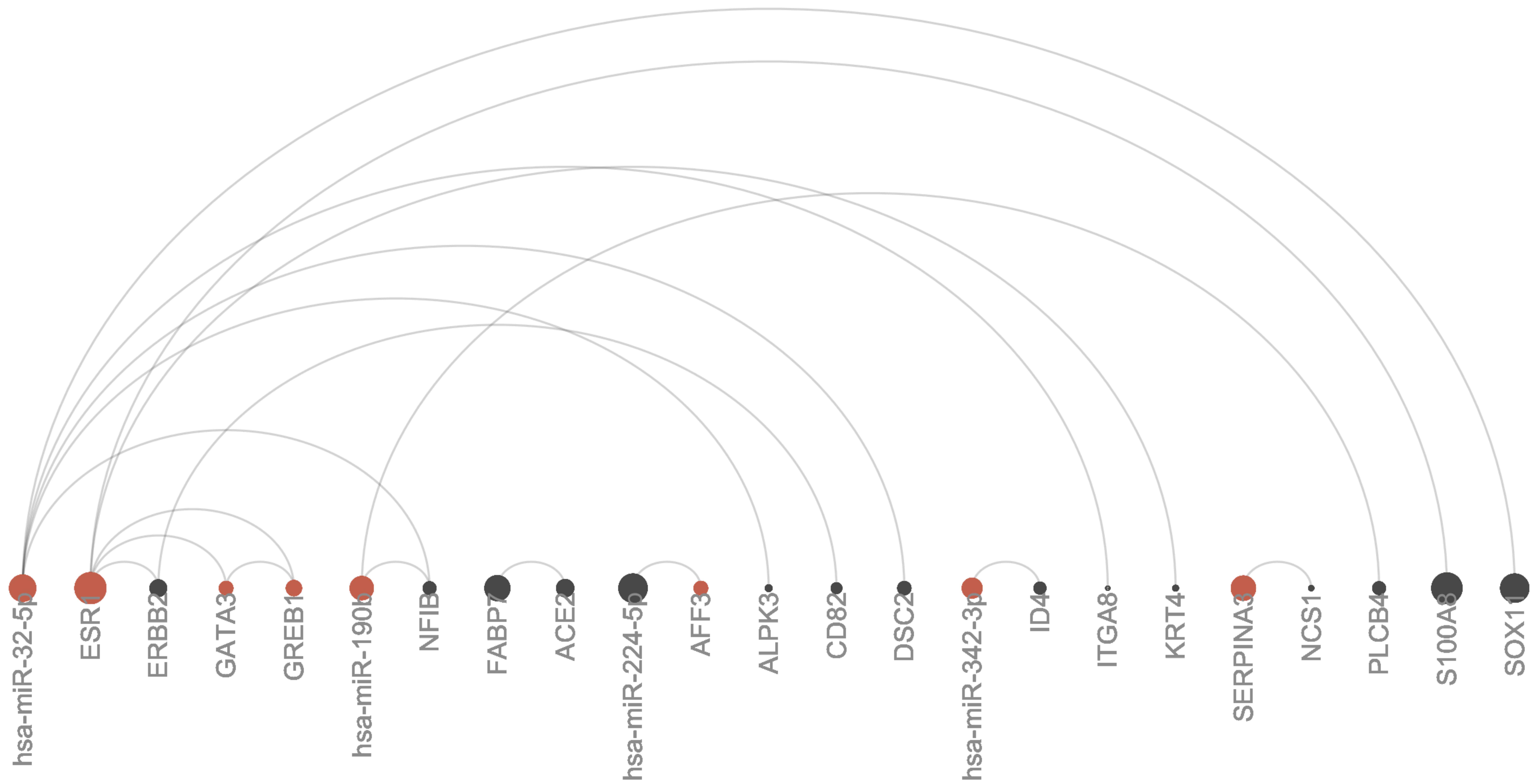

Supplement: Supplementary file 5 — Additional file 5: Figure S3. miRNA-Gene Interaction Networks. Networks of differentially expressed miRNAs and gene targets predicted by TargetScan. Colors refer to expression directionality, red = up-regulated, black = down-regulated. S3A = TIF Cluster 1 vs Cluster 2, S3B = High TILs (+2|+3) vs low TILs (0|+1), S3C = High-grade (gr 3) vs medium/low-grade (gr 1|2), S3D = Her2 vs TNBC, S3E = luminal A vs TNBC, S3F = luminal B vs TNBC, S3G = ER+ vs ER-. [file 13058_2020_1295_MOESM5_ESM.pdf]
