# Supplementary material for: Secreted breast tumor interstitial fluid microRNAs and their target genes are associated with triple-negative breast cancer, tumor grade, and immune infiltration
Source: Breast Cancer Res. 2020 Jun 30;22:73. doi: 10.1186/s13058-020-01295-6 (PMC7329449; doi:10.1186/s13058-020-01295-6)
Supplement: Supplementary file 12 — Additional file 12: Figure S8. Results of miRNA Co-abundance Network Analysis. WGCNA resulted in three miRNAs co-abundance modules, denoted Module 1 (Blue, S7 A), Module 2 (Turquoise S7 B) and Module 3 (Red, S7 C). Shapes indicate which contrast a given miRNA was differentially abundant within. X-axis = name of miRNA, y-axis = log fold change for miRNA in contrast. [file 13058_2020_1295_MOESM12_ESM.pdf]

Fig. S8

Fig. S8 A

Co-abundant miRNAs Module 1 (Blue Module)

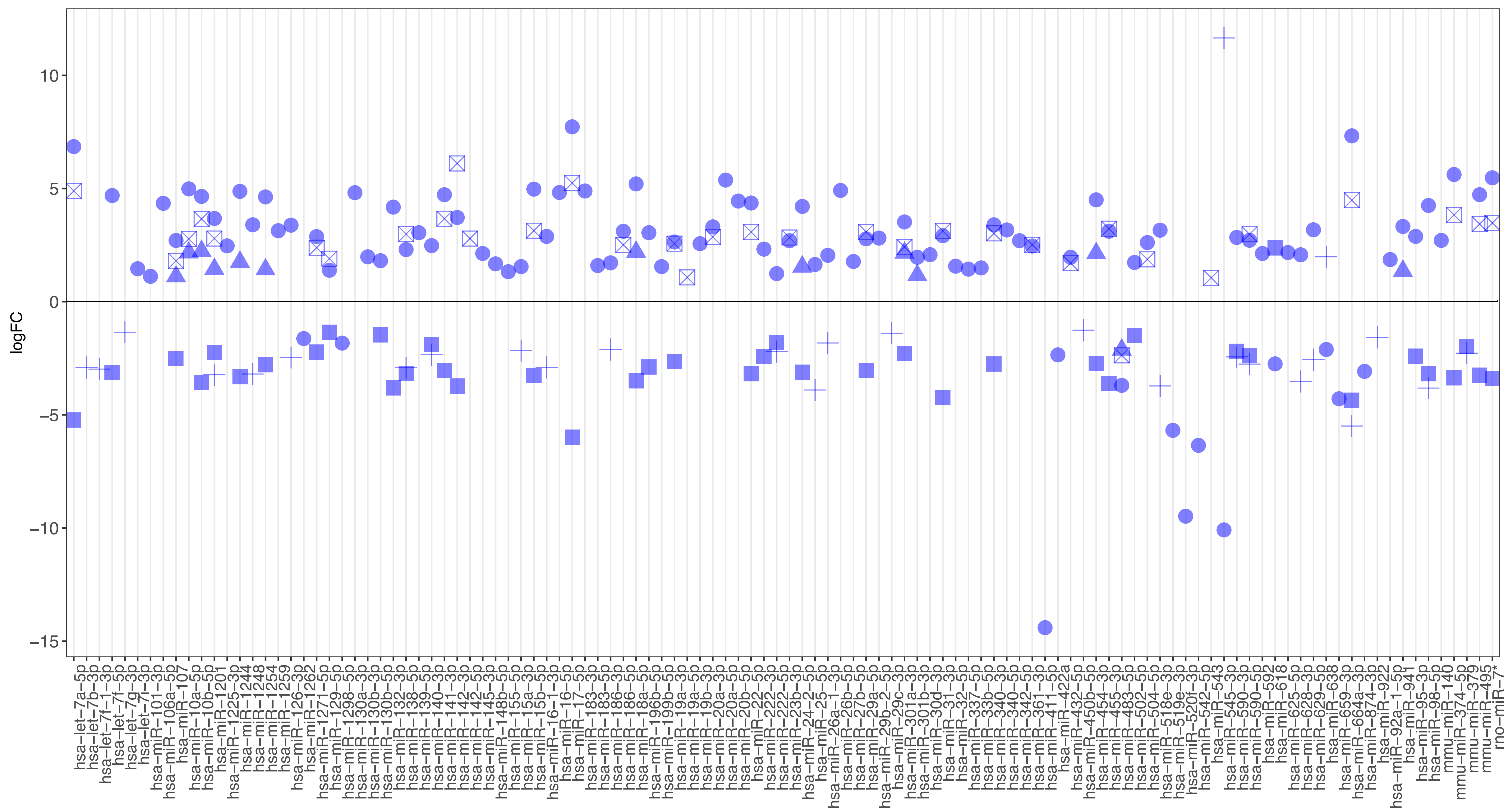

Fig. S8 B

Co-abundant miRNAs Module 2 (Turquoise Module)

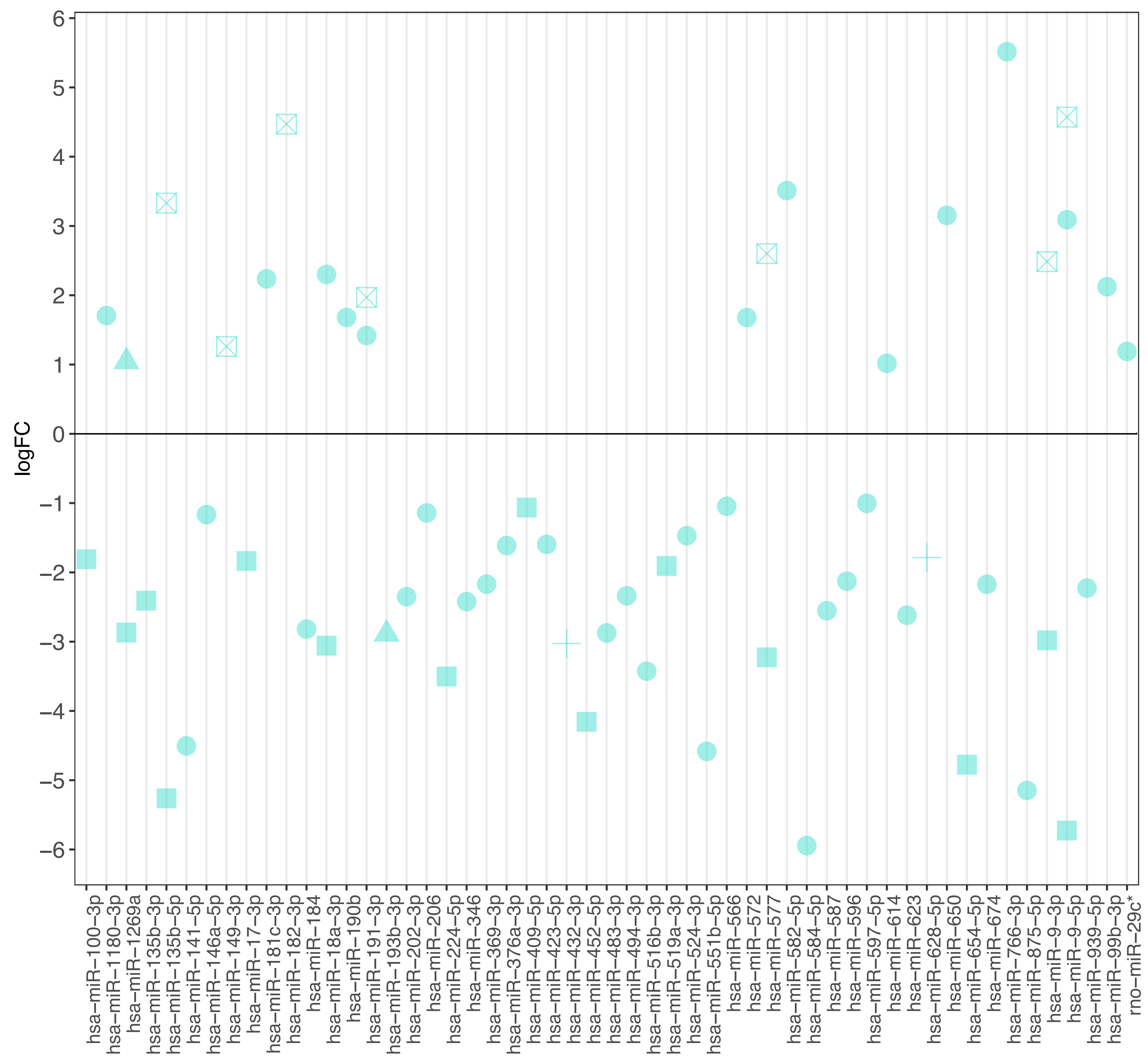

Fig. S8 C

Co-abundant miRNAs Module 3 (Red Module)

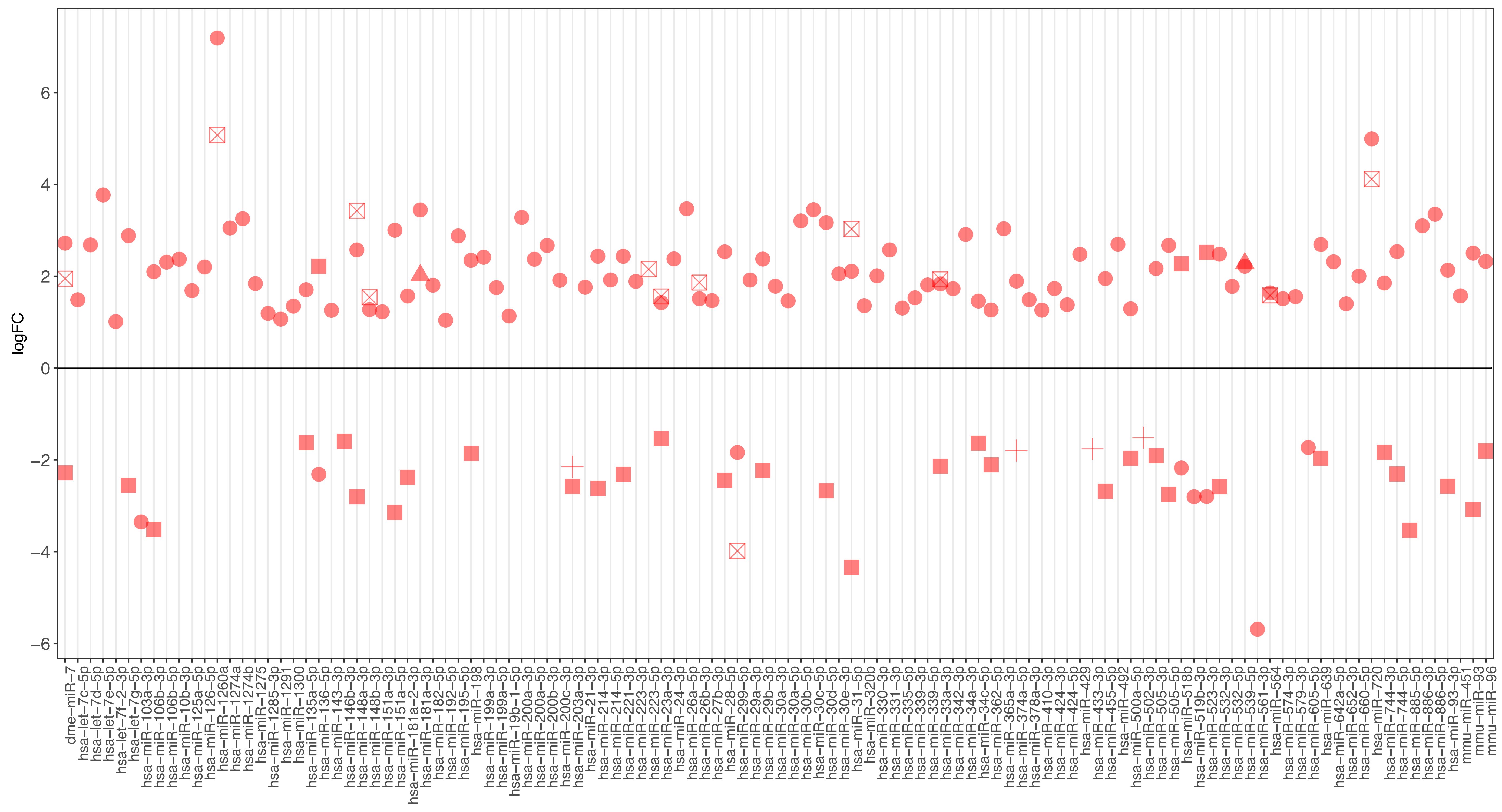

- Cluster 1 vs Cluster 2
- High Grade vs Medium/Low Grade
- Other Subtype vs TNBC
- PgR+ vs PgR-
- High TILs vs Low TILs
